# ^1^H NMR-Based Chemometrics to Gain Insights Into the Bran of Radiation-Induced Colored Wheat Mutant

**DOI:** 10.3389/fnut.2021.806744

**Published:** 2022-01-04

**Authors:** Yun-Seo Kil, Ah-Reum Han, Min-Jeong Hong, Jin-Baek Kim, Pil-Hoon Park, Hyukjae Choi, Joo-Won Nam

**Affiliations:** ^1^College of Pharmacy, Yeungnam University, Gyeongsan-si, South Korea; ^2^Advanced Radiation Technology Institute, Korea Atomic Energy Research Institute, Jeongeup-si, South Korea; ^3^Research Institute of Cell Culture, Yeungnam University, Gyeongsan-si, South Korea

**Keywords:** colored wheat bran, radiation, nuclear magnetic resonance, chemometrics, polar metabolites

## Abstract

Recently, wheat has attracted attention as a functional food, rather than a simple dietary energy source. Accordingly, whole-grain intake increases with an understanding of bioactive phytochemicals in bran. The development of colored wheat has drawn more attention to the value of bran owing to its nutritional quality, as well as the antioxidant properties of the colorant. The present ^1^H NMR-based chemometric study evaluated the compositional improvement of radiation-induced mutants in purple wheat by focusing on the predominant metabolites with high polarity. A total of 33 metabolites, including three choline derivatives, three sugar alcohols, four sugars, 13 amino acids, eight organic acids, and two nucleosides, were identified throughout the ^1^H NMR spectra, and quantification data were obtained for the identified metabolites via peak shape-based quantification. Principal component and hierarchical cluster analyses were conducted for performing multivariate analyses. The colored original wheat was found to exhibit improvements compared to yellow wheat in terms of the contents of primary metabolites, thus highlighting the importance of conducting investigations of polar metabolites. The chemometrics studies further revealed mutant lines with a compositional enhancement for metabolites, including lysine, proline, acetate, and glycerol.

**Graphical Abstract:**
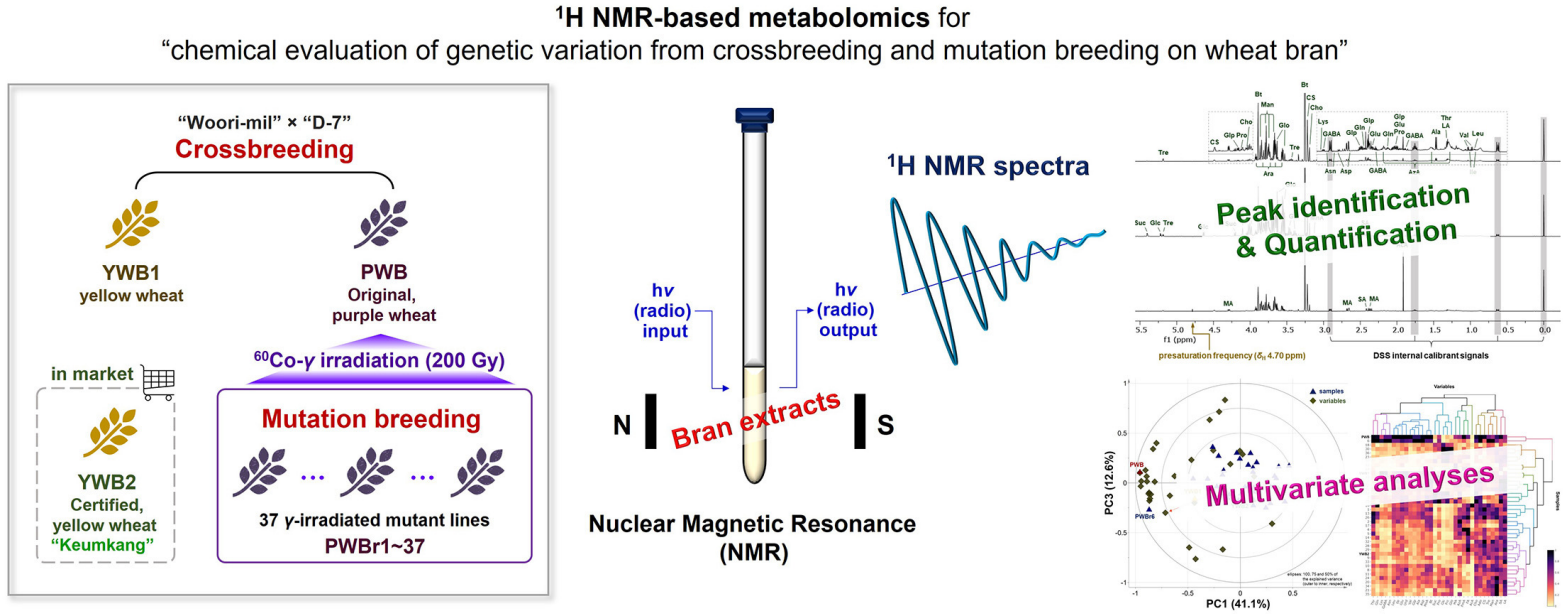


## Introduction

Wheat products such as bread, cereals, cookies, and biscuits are major parts of the human diet, making wheat (*Triticum aestivum* Linn., Poaceae) one of the most consumed ingredients worldwide. As sensory expectations and satisfaction are considered critical aspects of wheat consumption, milling processes have been developed to remove the fiber-rich bran layer and to retain the starch-rich endosperm portion of wheat (1). However, scientific studies conducted on wheat bran (or whole grain) have proven its preventive effects exerted on chronic diseases, including hypertension, obesity, diabetes, cardiovascular disease, and cancer, thus ushering in a new era of wheat consumption (1, 2). The nutritional value of food has gained prominence as a key customer consideration. As a result, the whole grain market has expanded significantly, and this has posed challenges to the agricultural and food processing systems to meet both nutritional and sensory expectations (3). The colored wheat has been developed with the engagement of relentless efforts to increase the biological functionality of wheat. Liu et al. found remarkable antioxidant effects from purple wheat rather than red, yellow, and white wheat, and the outstanding activity was correlated with higher anthocyanin content, such as cyanidin 3-glucoside (4). Since the antioxidant colorant is expressed in the outer layer of wheat grain (4–6), it further encourages the consumption of bran and whole grain.

Nuclear magnetic resonance (NMR) spectroscopy is an essential tool that provides tremendous information for the structure determination of natural products. The recent adoption of NMR in metabolomics studies is due to its intrinsic ability for direct quantification, high reproducibility, and non-destructive nature. Particularly, ^1^H NMR-based metabolomics has proven to be effective and efficient, because ^1^H atoms are ubiquitous in molecules with high isotopic natural abundance (~99%), thereby allowing fast data acquisition for the detection of most metabolites (7, 8). Polar metabolites, such as amino acids and organic acids are difficult to be quantified in conventional LC systems with insufficient resolution between signals. GC-MS spectrometry has been utilized as a common technique in metabolomics of the polar metabolites, including the polar composition of wheat (9, 10). However, ^1^H NMR-based metabolomics has emerged as a revolutionary technique with a remarkable ability of direct quantification (8). Graham et al. performed NMR-based metabolomics on European wheat by identifying 13 polar metabolites, and the integral data were obtained from manual binning for statistical analysis (11). Shewry et al. also performed bucketing of ^1^H NMR spectra for peak integration and reported quantitative data of 26 polar metabolites, which were used in sequential studies to assess genetic variations in wheat samples (12, 13) and to evaluate the impacts of different farming systems (organic and conventional) on wheat growth (14). The present study adopted peak shape-based quantification using the Chenomx software by considering peak splitting patterns (singlet, doublet, triplet, and so on) with coupling constants (*J*) and the number of protons according to compound information from embedded or custom compound libraries. It also considers signal sum of the assigned metabolites overlaid on the experimental spectrum, which enables the achievement of more reliable quantification results, especially for highly overlapped regions. Previously, Coulomb et al. introduced the peak shape-based quantification technique for polar metabolites in wheat grains; however, the study was limited to the analysis of 12 metabolites with detailed 2D NMR spectral data obtained only for azelate and sebacate (15).

Strategic integration of genetic diversity into plant breeding has led to improvements in the nutritional quality and agricultural productivity of crops. Crossbreeding and mutation breeding are considered two conventional strategies adopted in plant breeding for genetic modification (16). Crossbreeding purposes taking advantages of two other parents, and mutation breeding is conducted with an aim to produce genetic varieties from an outstanding cultivar over a short period by increasing the mutation rate from spontaneous generation. The combination of the two classical approaches is also effective for generating genetic variations. The deep purple color wheat (PW) was developed by conducting a crossbreeding of “Woori-mil” × “D-7,” together with a yellow wheat (YW1) (17). The purple wheat cultivar was then subjected to gamma-irradiation technique of mutation breeding (18). Gamma rays are widely used as physical mutagens for mutagenesis to efficiently generate new genotypes (19). The previous investigations on the produced varieties analyzed their anthocyanin content and antioxidant potency, which in turn, were observed to be correlated with each other. Although the previous studies have mainly focused on changes in anthocyanin content with color change, other nutritional improvements in colored wheat have also been reported, including the presence of a higher amount of essential amino acids (20, 21). This motivated the present metabolomics analysis of highly polar, real principal components between the varieties produced via crossbreeding and mutation breeding. The chemometric study adopted gamma-irradiated mutants derived from colored (purple) wheat, together with the original sample (non-irradiated). Two yellow wheat samples were also included for comparative analyses of crossbred varieties. This report describes the development of a reliable qualitative and quantitative platform using a ^1^H NMR-based metabolomics approach, followed by the discovery and obtainment of distinct chemical profiles between the wheat varieties in multivariate analyses.

## Materials and Methods

### General Experimental Procedures

The 1D and 2D NMR spectra were recorded on a 600 MHz Varian NMR spectrometer (VNS-600, Palo Alto, CA, USA), operated using Bruker TopSpin software (Billerica, MA, USA), at the Core Research Support Center for Natural Products and Medical Materials (CRCNM). The acquired data were processed using MestReNova 12.0.3 software (Mestrelab Research SL, Santiago de Compostela, Spain). Analytical HPLC was conducted using a Shimadzu LC-20A system (Kyoto, Japan) equipped with a Shimadzu SPD-M20A photodiode array (PDA) detector (Kyoto, Japan) and an Alltech 3300 evaporative light scattering detector (ELSD, Essen, Germany), with a Phenomenex Luna 5 μm C_18_ column (100 Å, 250 × 4.6 mm, 1 mL/min). Deuterated solvents, including 3-(trimethylsilyl)-1-propanesulfonic acid solution (DSS, an internal calibrant, 1 wt% in D_2_O, 99.9 atom % D) and sodium deuteroxide solution (NaOD, 40 wt% in D_2_O, 99.5 atom % D) for NMR experiments, as well as monopotassium phosphate (KH_2_PO_4_, ≥99.0%), were purchased from Sigma-Aldrich. Azelaic acid (98%), sebacic acid (98%), and glycerophosphocholine (choline alfoscerate, 98%) were obtained from AK Scientific. Choline sulfate (98%) and phosphocholine chloride calcium salt tetrahydrate (>98%) were purchased from Cambridge Isotope Laboratories and TCI Chemicals, respectively. All solvents were of ACS grade or better.

### Wheat Bran Samples

The seeds of a colored cultivar (purple wheat; PW) were subjected to treatment with 200 Gy of gamma (^60^Co) irradiation to generate wheat mutant lines, as per methods previously described (18). The mutants with stable phenotype inheritance for 4 years were selected and cultivated for further studies by Dr. Jin-Beak Kim and Dr. Min-Jeong Hong (Korea Atomic Energy Research Institute); they were named PWr1-37 (radiation-bred, purple wheat). The voucher specimens were deposited at the Radiation Breeding Research Center, Advanced Radiation Technology Institute, Korea Atomic Energy Research Institute. For the present study, seeds of the selected mutants (PWr1–37) were sown, germinated, and grown, together with two yellow wheat seeds (YW1 and YW2), in the same manner as previously reported (22). YW1 and PW were developed from the cross “Woori-mil (Korea RDA accession no. IT172221)” × “D-7” (an inbred line developed by Korea University) (17). YW2 is “Keumkang (or Geumgang),” a certified cultivar in the market in Korea. Dried bran (PWB, PWBr1-37, YWB1, and YWB2; 1 g each) were individually extracted with MeOH (20 mL) by ultrasonication for 1 h. The extracts were evaporated *in vacuo* to dryness and stored at −20°C.

### NMR Sample Preparation

The dried sample extracts were kept in a vacuum oven for 1 day before use. 10.0 mg of each sample was weighed into an Eppendorf tube. A phosphate buffer was prepared by adding 1.0 M NaOD to 90 mM KH_2_PO_4_ in D_2_O containing 0.02 wt % DSS (23). The pH was adjusted to 6.0, under pH meter monitoring. The pH-adjusted solution (700 μL) was added to each sample, and the mixture was ultrasonicated for 5 min. To obtain a clear sample solution for the NMR experiment, each sample was centrifuged at 13500 rpm for 5 min and then filtered through a polytetrafluoroethylene syringe filter (0.45 μm). Next, 600 μL of the filtered sample solution was transferred into 5 mm NMR tubes.

### NMR Data Acquisition

For the ^1^H NMR metabolomics analyses, 1D NOESY presat pulse sequence (noesypr1d, TopSpin, Bruker) was used for water signal suppression with δ_H_ 4.70 ppm of a presaturation frequency. The ^1^H NMR data were acquired for all 40 samples with the following parameter settings: probe temperature at 298 K, spinning off, calibrated 90 degrees pulse (P1), relaxation delay (D1) of 2 s, acquisition time (AQ) of 4 s, spectral width (SW) of 16 ppm (centered at 4.70 ppm), receiver gain (RG) of 64, number of scans (NS) of 64, and number of dummy scans (DS) of 2.

The ^13^C, ^1^H-^1^H COSY, ^1^H-^13^C HSQC, and ^1^H-^13^C HMBC NMR data were obtained for three representative samples PWB, PWBr3, and PWBr19 to get further information supporting the preliminary assignment. All NMR spectra were acquired at probe temperature of 298 K with spinning off. The acquisition parameters for each experiment were as follows: for the ^13^C NMR, D1 2s, AQ 0.9175 s, SW 236 ppm (centered at 100 ppm), NS 50000; for ^1^H-^1^H COSY NMR, data matrix 4,096 (F2) × 256 (F1) points, D1 2s, AQ 0.9175 s (F2) and 0.0328 s (F1), NS 16; for ^1^H-^13^C HSQC NMR, data matrix 2,048 (F2) × 256 (F1) points, D1 2s, AQ 0.0860 s (F2) and 0.0036 s (F1), NS 32; for ^1^H-^13^C HMBC NMR, data matrix 4,096 (F2) × 256 (F1) points, D1 2s, AQ 0.1720 s (F2) and 0.0072 s (F1), and NS 128.

### NMR Data Processing

The obtained ^1^H NMR data were processed using MestReNova. The chemical shifts were referenced to the singlet signal of the DSS methyl groups at δ_H_ 0.00 ppm. Fifth-order polynomial fitting was applied for baseline correction, and manual phasing was carefully conducted. Lorentzian-to-Gaussian apodization (exponential factor of −0.3 and Gaussian factor of 0.05) was used for resolution enhancement.

The processed data were profiled using Chenomx NMR Suit 8.4 software (Edmonton, AB, Canada). The Chenomx 600 MHz library (ver. 10) was utilized for NMR signal identification in the metabolomics analysis. A custom compound library was prepared for the quantification of choline sulfate, and the existing compound library file of choline was modified based on the experimental ^1^H NMR data of choline sulfate, which was acquired as a 2.00 mg/mL D_2_O solution (10.7 mM of choline sulfate of 98% standard sample purity) under the same parameter settings used for the sample data collection.

### Spiking ^1^H NMR Analyses for Identification of Undefined Compounds

Spiking ^1^H NMR analyses were performed for the unambiguous assignment of azelate in the wheat bran samples (24). By considering the quantification values of azelate from the preliminary profiling in Chenomx, a stock solution of the commercial standard was prepared at a concentration of 0.3 mg/mL. The stock solution (25 μL) was added to a PWB NMR sample, which was prepared using 5.0 mg of PWB in the general sample preparation method. The same spiking experiment was also conducted using a commercial sebacate standard.

The primary assignments using the Chenomx library remained an unidentified component of δ_H_ 3.22, 3.75, and 4.99. Spiking ^1^H NMR analyses were conducted to narrow the net to choline sulfate (24). Commercial standards of probable metabolites (phosphocholine, glycerophosphocholine, and choline sulfate) were prepared at a concentration of 20 mg/mL as stock solutions. The spiking experiments were performed by adding stock solutions, as performed for azelate.

### Statistical Analysis

The data matrices preprocessed using Chenomx were imported into SIMCA 15.0.2 (Umetrics, Malmö, Sweden) for the statistical interpretation of the metabolomics analysis. Principal component analysis (PCA) was performed using unit variance scaling. The R^2^ and Q^2^ parameters were indicative of the quality of the models for model fitness and predictive ability, respectively. A heat map with hierarchical clustering was generated using “heatmaply” (25) and “dendextend” (26) packages in R software (version 4.1.0).

## Results and Discussion

### Selection of a Suitable Spectroscopic Technique for Wheat Bran Metabolomics

The present study aimed to develop an efficient qualification and quantification tool for the evaluation of genetic varieties obtained via crossbreeding and mutation breeding. As a preliminary study, the wheat bran extracts were analyzed in the HPLC-PDA-ELSD system, and a huge, merged peak was observed within 4 min of each run (Supplementary Figure 1). Large peaks were detected only on the ELSD detector, whereas no corresponding signals were observed on the PDA chromatograms. On the other hand, peaks observed in PDA detection at UV 254 nm were noted with typical UV/Vis absorption profiles of flavonoids, and the presence of flavonoids and their glycosides in wheat was previously reported in MS/MS-based metabolomics studies (27, 28). However, in our study, the intensity of the signals attributable to flavonoids was markedly weak considering the concentration of the injected crude sample and the intensity of the considerable ELSD signal observed within 4 min of the run. Moreover, no significant signals were observed in the corresponding ELSD chromatograms (the signal intensity was considered to be lower than the detection limit). The overall observation suggested that highly polar metabolites primarily contributed to the chemical composition of the wheat bran extracts, posing challenges in the performance of the conventional LC-based analysis equipped with reversed-phase C_18_ columns. In other words, the resolution between the signals of polar metabolites was insufficient for qualitative or quantitative analysis. To address the issue encountered with the conduction of the conventional C_18_ LC analysis, NMR spectroscopy was introduced into the present qualitative and quantitative study.

To determine an appropriate deuterated solvent for ^1^H NMR data acquisition, DMSO-*d*_6_, CD_3_OD, and D_2_O were selected for the test, considering the solubility of the wheat bran samples. When DMSO-*d*_6_ was used, the observation of the hydroxyl groups of polar metabolites resulted in the complexity of the spectrum. The ^1^H NMR spectra in CD_3_OD and D_2_O showed a substantial difference only in the intensity of the broad signal at δ_H_ 1.2–1.5 ppm, typically corresponding to lipids (more intense in CD_3_OD than D_2_O, Supplementary Figure 1). Thus, D_2_O was selected for the present NMR-based metabolomics study focused on the investigation of major, highly polar components of wheat bran. It also presented with the advantages of utilizing the embedded libraries in Chemonx software and the reference 1D and 2D NMR spectra available online for free from the Biological Magnetic Resonance Bank (BMRD) (29).

### Identification of Metabolites in ^1^H NMR Spectra

^1^H NMR spectra were acquired for 40 wheat bran extracts in phosphate buffer (pH 6.0, in D_2_O) containing 0.02 wt% DSS (23). The intense water signal was suppressed in the ^1^H NMR spectrum by using “1D NOESY presat” pulse sequence (presaturation frequency at δ_H_ 4.70 ppm) (30). The 40 spectral data obtained (Supplementary Figure 2) shared the same fixtures with signal condensation at δ_H_ 3.5–4.0 ppm. Three distinctive singlet signals were observed at δ_H_ 3.25, 3.22, and 3.19 ppm, which were attributed to the presence of three other choline derivatives (11, 12). This is in accordance with the finding reported in previous studies, indicating that betaine and choline are the predominant metabolites of wheat.

**Figure 1:**
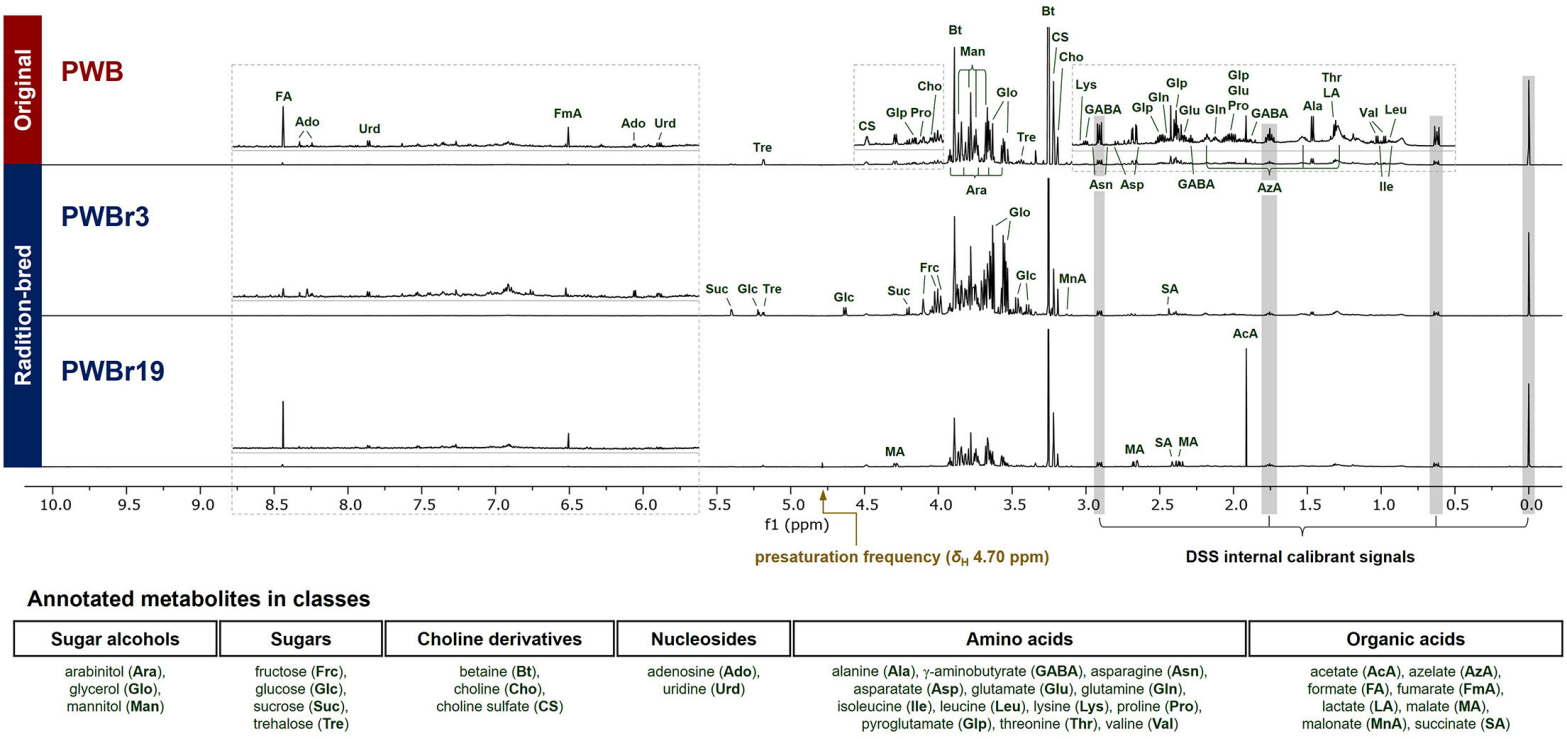
Annotated ^1^H NMR spectra (600 MHz, D_2_O) of three representative purple wheat bran samples (top to bottom, PWB, original; PWBr3 and 19, radiation-bred). The “1D NOESY presat” pulse sequence was used for data acquisition with δ_H_ 4.70 ppm of a presaturation frequency. PWB, purple wheat bran; PWBr, radiation-bred mutant of purple wheat bran.

Analysis of the ^1^H NMR spectra in Chenomx software using the embedded library enabled the primary assignment of 32 metabolites, including the major components betaine and choline. Among the 40 samples investigated, PWB (original, purple wheat bran), PWBr3, and PWBr19 (radiation-bred, purple wheat bran) were analyzed as three representatives, as shown in Figure 1. A mutant line, PWBr3, showed more intense signals in the range of δ_H_ 4.0–5.5 ppm, compared to the original PWB, indicating the presence of greater quantities of sugars (glucose, sucrose, trehalose, among others). Two doublets of glycerol methylene at δ_H_ 3.64 and 3.55 ppm were observed to be more apparent in some of the samples including PWBr3. In the case of another mutant line PWBr19, the ^1^H NMR singlet signal of the acetate methyl group (δ_H_ 1.91 ppm) was observed to be higher than that of the original PWB. The malate ^1^H NMR resonances were found to be remarkable in the spectrum at δ_H_ 4.29, 2.67, and 2.38 ppm. The ^13^C, ^1^H-^1^H COSY, ^1^H-^13^C HSQC, and ^1^H-^13^C HMBC NMR spectra of the three representative samples were acquired (Supplementary Figures 17–20 for PWB, Supplementary Figures 21–24 for PWBr3, Supplementary Figures 25–28 for PWBr19) to provide further information confirming the preliminary assignment (Supplementary Table 1). Detailed NMR data analyses were performed using the reference 1D and 2D NMR data available in BMRD, the free online database (BMRD codes of each metabolite have been listed in Supplementary Table 2) (29). The identification of pyroglutamate in the wheat bran extracts was further validated upon comparison of the 1D and 2D NMR data with reported values (31).

The significant peaks in the thick area (δ_H_ 3.5–4.0) were annotated as sugar alcohols and sugars (Figure 1), which contributed to the density by the presence of oxygenated methylene and methine groups in their structures (29). Glycerol, arabinitol, and mannitol are the sugar alcohols identified in the samples, and they have different chain lengths in the general formula: HOCH_2_(CHOH)_n_CH_2_OH (glycerol, *n* = 1, three-carbon chain; arabinitol, *n* = 3, five-carbon chain; mannitol, *n* = 4, six-carbon chain). Due to the difference in the chain length, they could be distinguished from each other in ^1^H NMR spectrum with marked resonances including δ_H_ 3.55 (dd, *J* = 11.6, 6.5 Hz, H-1b and H-3b of glycerol), 3.79 (d, *J* = 8.5 Hz, H-3 and H-4 of mannitol), and 3.92 (ddd, *J* = 7.4, 5.5, 2.2 Hz, H-4 of arabinitol) (Supplementary Table 1). A thorough interpretation of the 2D NMR correlations of the three sugar alcohols further supported the assignments (Supplementary Figures 3–5).

The ^1^H NMR signals, predominant in PWBr3 in a range of δ_H_ 4.0–5.5 ppm (Figure 1), were readily assigned to four sugars of two monosaccharides (fructose and glucose) and two disaccharides (sucrose and trehalose). The anomeric protons of glucose, sucrose, and trehalose are distinctive at δ_H_ 4.63 (d, *J* = 8.0 Hz, H-1 of β-glucose), 5.22 (d, *J* = 3.8 Hz, H-1 of α-glucose), 5.40 (d, *J* = 3.9 Hz, H-1 of sucrose), and 5.18 (d, *J* = 3.8 Hz, H-1 and H-1′ of trehalose), respectively (Supplementary Table 1). The β-pyranose mutarotamer of fructose showed distinguishable ^1^H NMR resonances at δ_H_ 3.98 (dt, *J* = 3.5, 1.3 Hz, H-5) and 4.01 (dd, *J* = 12.7, 1.3 Hz, H-6a). The dynamic isomerism of monosaccharide fructose and glucose in solution results in the complexity of the NMR spectra, revealing the presence of mutarotameric isomers by tautomerization. Previous experimental studies have shown that glucose exists at ~63% of β-glucopyranose and ~37% of α-glucopyranose (32), and fructose exists in three predominant isomers: β-fructopyranose (~70%), β-fructofuranose (~21%), and α-fructofuranose (~6%), in equilibrium status (in neutral pH water and at room temperature) (33). Chenomx software counts the properties to yield quantity information. The ^13^C NMR spectrum of PWBr3 also showed signals for each mutarotameric isomer of glucose and fructose (Supplementary Figure 6). Moreover, peak integrations of the ^13^C NMR signals provided additional experimental observations supporting the reported populations of the predominant isomers.

Organic acids are a class of compounds that are difficult to be quantified using LC-based analysis (7, 8). Here, eight organic acids were identified. Since organic acids possess carboxylic acid functional groups, the co-existence of neutral acid and anion forms causes inconsistency in chemical shifts (24, 34), particular attention is required while assigning NMR signals. Among the identified organic acids, acetate and malate showed substantial variations in chemical shifts between samples, while some of them showed ^1^H NMR resonances out of the range suggested in the Chenomx-embedded library. In the case of malate, some of the mutant lines (PWBr4, 5, 12, 14, 16, 19, 26–29, and 33) showed ^1^H NMR signals of H-3b out of the reliable range for identification suggested in the Chenomx library (Supplementary Figure 7). From the detailed 2D NMR data analyses of malate in PWB, the ^1^H-^1^H COSY correlation of H-2/H-3b was observed as one of the key 2D correlation data for structure identification (Supplementary Figure 8). To verify the identification of malate in the mutant lines described above, the key ^1^H-^1^H COSY correlation was additionally analyzed in the ^1^H-^1^H COSY spectra of PWBr14 and PWBr19 (Supplementary Figure 9). Additional analyses further supported the assignment of malate, which suggested a wider range of the H-3b chemical shift, reliable for identification, as δ_H_ 2.3620–2.4140 ppm of cluster center (δ_H_ 2.3732–2.4140 ppm in the Chenomx-embedded library, Supplementary Figure 7).

The identified metabolites include azelate, a dicarboxylic acid compound, whose presence in wheat has been reported in previous MS-based metabolomics studies (9, 10), while one study involving the use of NMR-based metabolomics has suggested that azelate exists together with sebacate and presents with two sets of triplet signals (15). The ^1^H NMR spectra in the present study also showed two overlapping triplets at δ_H_ 2.18 ppm. However, the correlation data from ^1^H-^1^H COSY and ^1^H-^13^C HMBC spectra could not provide insights for the annotation of the overlapping signals as specific dicarboxylic acid compounds, including azelate and sebacate. To clarify this issue, spiking experiments were performed using commercial standards of azelate and sebacate (24). The assignment of azelate was supported by the spiking study performed in the ^1^H NMR experiments, when the other triplet was expected to be derived from another dicarboxylic acid compound and not sebacate (Supplementary Figure 10). In the present study, only azelate, the identification of which was confirmed, was quantified in wheat bran samples.

**Figure 2:**
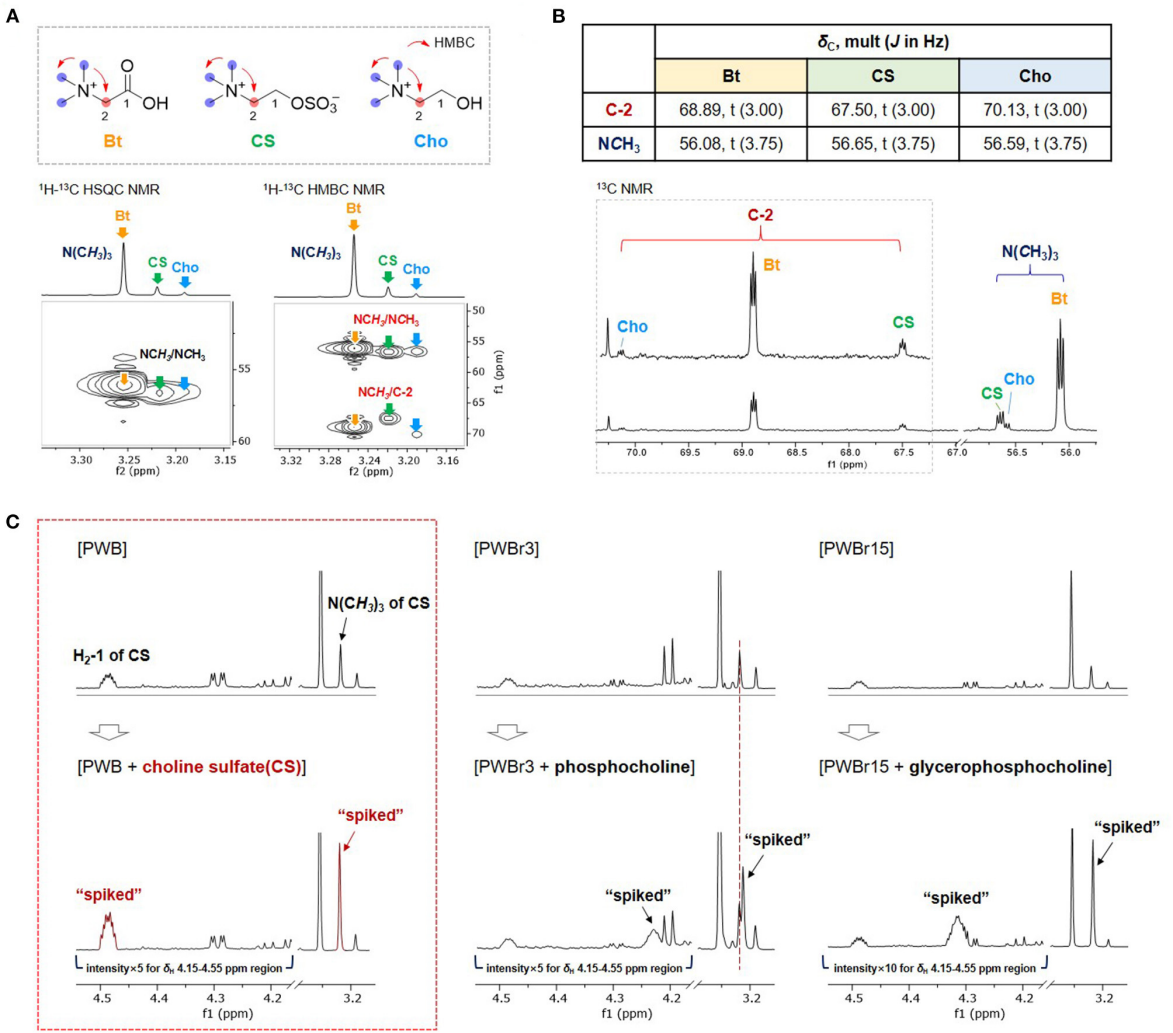
Comparison of **(A)** ^1^H-^13^C HSQC and HMBC correlations and **(B)** ^13^C NMR signals of C-2 and N*C*H_3_ of betaine (Bt), choline sulfate (CS), and choline (Cho) in PWB. **(C)** Spiking ^1^H NMR experiments with commercial standards of CS, phosphocholine, and glycerophosphocholine.

Betaine and choline were identified from two of the singlet proton signals at δ_H_ 3.19, and 3.25, respectively, as assigned previously (11, 12, 15). However, the other singlet at δ_H_ 3.22 and the correlated signals (δ_H_ 4.49, 3.75) remained a puzzle in the primary assignment; although phosphocholine and glycerophosphocholine were suggested as strong candidates based on the similarity on their respective ^1^H NMR signals, the ^1^H NMR signal δ_H_ 4.49 ppm didn't match with any of the reference data in the Chenomx-embedded libraries and the previous reports on them (phoshocholine, δ_H_ 4.18 ppm; glycerophosphocholine, δ_H_ 4.32 ppm) (35). Small-Molecule Accurate Recognition Technology (SMART) is a newly developed web-based platform for proposing structural hypotheses based on ^1^H-^13^C-HSQC correlation data (36). SMART was also used to puzzle out the piece by using correlation data extracted from the ^1^H-^13^C HSQC NMR spectrum of PWB (δ_H_/δ_C_ 3.22/56.6, 3.75/67.5, and 4.49/64.7), which resulted in the achievement of phosphocholine again with high cosine scores of 0.95. To verify the result, spiking ^1^H NMR experiments were further conducted using commercial standards of phosphocholine and glycerophosphocholine; none of them showed a match with the unidentified entity (Figure 2C). However, the similar pattern of the 2D NMR correlations, including ^1^H-^13^C HMBC correlations of NC*H*_3_/N*C*H_3_ and C-2, supported the presence of another choline derivative (Figure 2A and Supplementary Figures 11–13). The unidentified component also shared an additional distinct feature with choline and betaine in the ^13^C NMR spectrum (Figure 2B). The ^13^C NMR signals attributable to C-2 and N*C*H_3_ were observed as triplets [δ_C_ 56.08 (t, *J* = 3.75 Hz, N(*C*H_3_)_3_ of betaine), 56.59 (t, *J* = 3.75 Hz, N(*C*H_3_)_3_ of choline), 56.65 (t, *J* = 3.75 Hz, N(*C*H_3_)_3_ of the unidentified component), 67.50 (t, *J* = 3.00 Hz, C-2 of the unidentified component), 68.89 (t, *J* = 3.00 Hz, C-2 of betaine), 70.13 (t, *J* = 3.00 Hz, C-2 of choline)]. This phenomenon has been reported for choline derivatives as a consequence of the one-bond ^13^C-^14^N coupling (^1^*J*_CN_) (37– 39). The highly symmetric chemical environment of the choline nitrogen nucleus induces considerably slower relaxation, resulting in the observation of substantial ^13^C-^14^N coupling. The nucleus ^14^N has a spin quantum number of 1 as same as ^2^H (deuterium), which allows the occurrence of triplets with a spin state number of 3. The observation of distinct ^13^C-^14^N coupling triplets further supported the presence of another choline derivative. After thorough investigation, we suggested the possibility of the presence of sulfate units instead of phosphate, inspired by reports stating that sulfur deficiency in the soil could lead to the accumulation of sulfate-containing metabolites in the plants as a sulfur resource (40, 41). Comparison of the ^1^H NMR signals with the reported values helped to suggest choline sulfate for the unidentified entity (42). A spiking ^1^H NMR experiment was conducted using a commercial standard to confirm the identification of choline sulfate (Figure 2C) (24).

Quantification of choline sulfate on Chenomx was performed by introducing a custom compound library prepared based on the experimental data of the commercial standard (Supplementary Figure 14). Choline sulfate is not a species-specific metabolite and its occurrence has been reported from various natural sources of plants (40), fungi (43), and bacteria (44). Despite the broad spectrum of its occurrence, choline sulfate has not been included in the accessible database such as Chenomx, BMRD, and HMDB (the Human Metabolome Database; experimental or predicted NMR data have not been provided with the given code “HMDB0250194”). A sequential study reported by a Chinese institute identified choline sulfate in the ^1^H NMR-based metabolomics of crab paste, *Pyropia haitanensis*, and pickled wax gourd previously (45); however, there was a lack of description on the coupling effects from highly symmetric ^14^N nucleus. The present study provides custom compound library of choline sulfate, together with detailed 1D and 2D NMR data acquired in D_2_O (Supplementary Table 1 and Supplementary Figures 29–33). Other than the substantial one-bond ^13^C-^14^N couplings (^1^*J*_CN_), two- or three-bond ^1^H-^14^N couplings (^2^*J*_HN_ or ^3^*J*_HN_) are also resolvable in the ^1^H NMR data of choline derivatives, which have been utilized for the development of 2D ^1^H-^14^N NMR techniques for quantitative analyses of choline-containing samples (46). However, the presence of ^1^H-^14^N coupling along with the non-first order coupling adds complexity to the ^1^H NMR data. Due to the complex ^1^H NMR signals, a custom compound library file of choline sulfate could not be completely generated with the “Spin Simulator” module in Chenomx suit (in other words, the peak shapes of the spin states generated in the “Spin Simulator” module did not match well with those obtained in the experimental ^1^H NMR data). Therefore, a custom library of choline sulfate was prepared by modifying the existing compound library file of choline to match the peak shape with the experimental ^1^H NMR spectrum of standard choline sulfate (Supplementary Figure 14).

In addition to the metabolites described above, 13 amino acids and two nucleosides were assigned to the ^1^H NMR spectra of the wheat bran samples (Figure 1). These assignments were further supported by the 2D NMR data analysis. Homonuclear 2D NMR experiments, such as ^1^H-^1^H COSY and TOCSY, are deemed indispensable for providing key coupling information for ^1^H NMR-based metabolomics (8, 23). The correlation data from the ^1^H-^1^H COSY NMR experiments were also informative for metabolite annotation in the present study. The methyl group at C-3 of lactate showed complete signal overlapping at δ_H_ 1.32 ppm with the methyl group at C-4 of threonine. In the analysis of the ^1^H-^1^H COSY NMR spectrum of PWB, the overlapped methyl group resonances showed two cross-peaks with signals at δ_H_ 4.10 (H-2 of lactate) and 4.24 ppm (H-3 of threonine), respectively (Supplementary Figure 15A). The correlations supported the co-existence of lactate and threonine in the samples, and quantification was performed in a thorough comparison of the signal sum of the identified metabolites with the experimental spectrum for all the proton signals corresponding to the two metabolites. In addition, the methyl group signals of valine, leucine, and isoleucine, were identified from each other by analysis of the ^1^H-^1^H COSY correlations [valine, δ_H_ 0.98 and 1.03 (H_3_-4 and H_3_-5)/2.27 (H-3); leucine, δ_H_ 0.95 (H_3_-5 and H_3_-6)/1.71 (H-4); isoleucine, δ_H_ 1.00 (H_3_-6)/1.97 (H-3)] (Supplementary Figure 15B). Analysis of amino acids in LC-based systems has been developed using various chemical derivatization methods with advanced techniques for detection (e.g., MS^*n*^ fragmentation); however, the isomeric amino acids such as leucine and isoleucine continue to complicate LC-based analysis (47). Therefore, the discriminative ability of NMR can be suggested as one of the specialties of NMR-based metabolomics.

### Multivariate Analyses

The concentrations of the identified polar metabolites in wheat bran were determined and expressed in “mM” using the Chenomx software and were then converted into “mg/g dried extract” by considering the molecular weights of each metabolite (Supplementary Table 3). Multivariate analyses were performed with quantification data using SIMCA-P and R software to generate plots in principal component analysis (PCA) and a heat map in hierarchical cluster analysis (HCA), respectively. The abbreviations of the identified metabolites were used in the following description and are listed in Figure 1.

**Figure 3:**
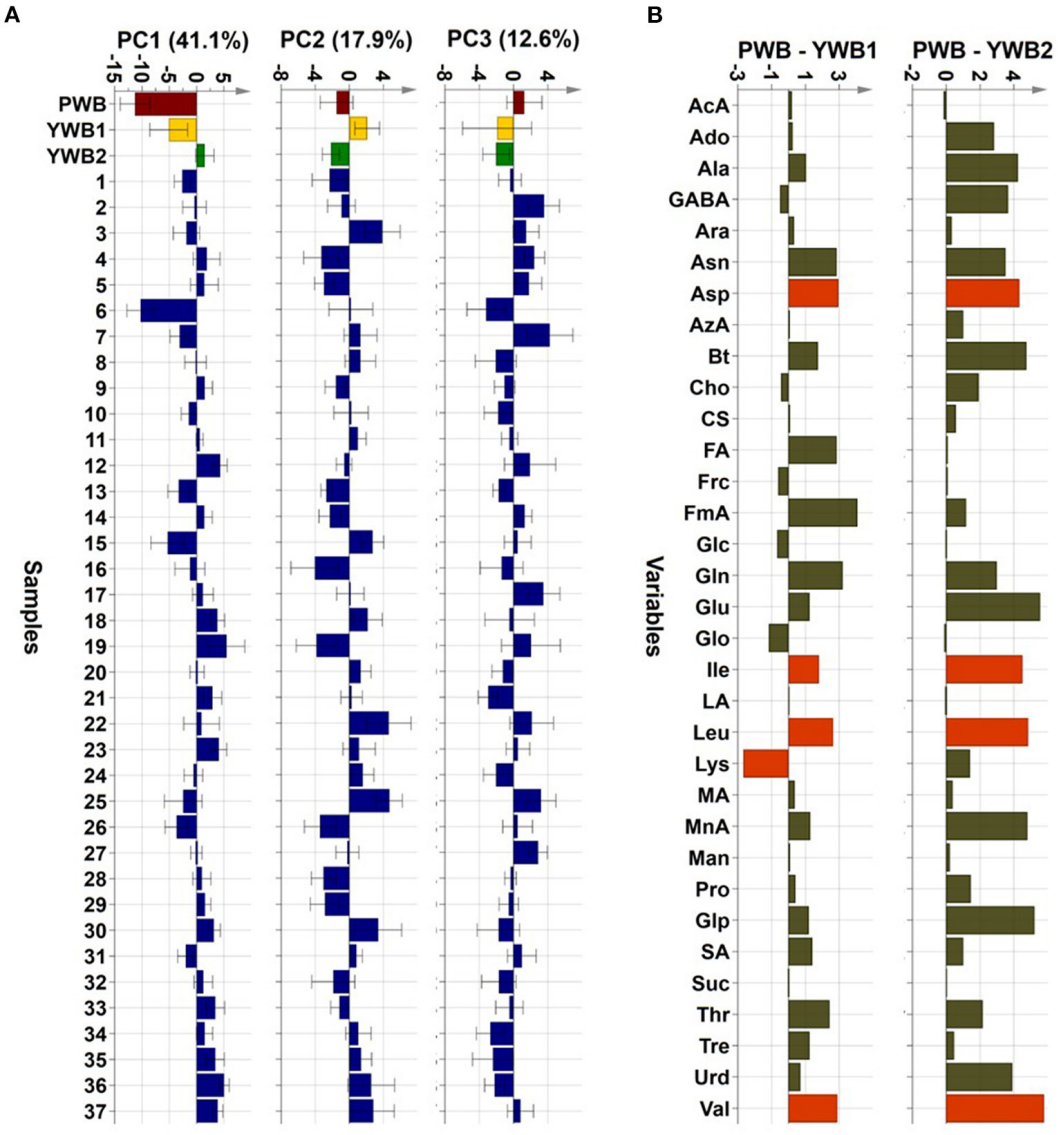
**(A)** PCA scores on the first three components (PC1, PC2, and PC3, respectively, left to right). **(B)** Contribution scores plots from YWB1 to PWB (left) and from YWB2 to PWB (right) (p1 loading was used as a single weight; variables outside three-fold standard deviation ranges are highlighted in orange).

**Figure 4:**
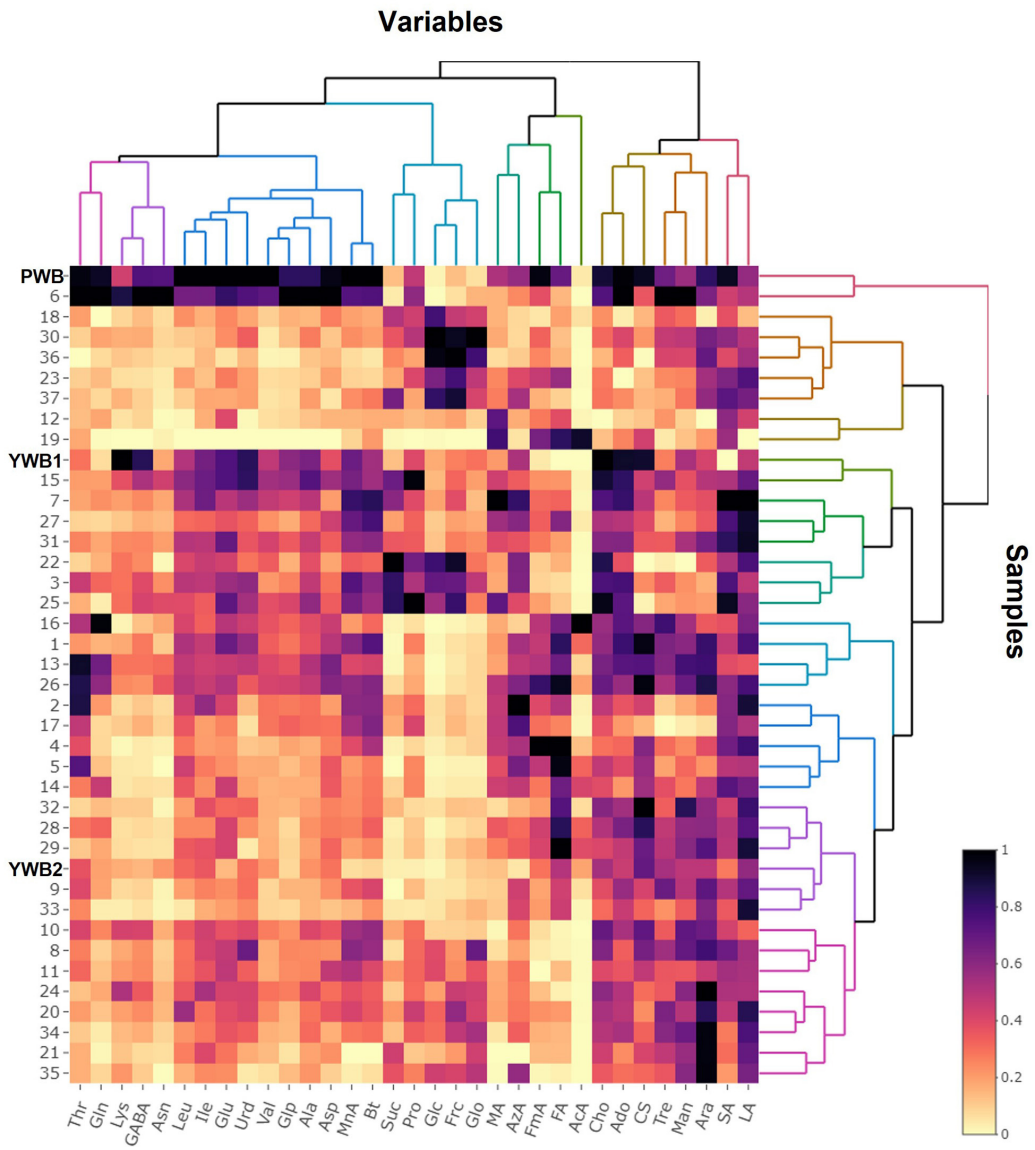
Heat map visualization of the normalized quantification data.

Uploading of the quantification data of 40 samples to SIMCA-P revealed five principle components (PCs, PC1 41.1, PC2 17.9, PC3 12.6, PC4 5.83, and PC5 4.61%). The degree of fitting of the generated PCA model to the dataset was indicated by 0.821 of R^2^ (82.1% of the total variation) and the predictive power was presented as 0.561 of Q^2^, which satisfied Q^2^ > 0.5 and R^2^ > Q^2^, a criteria for a good model (48). The bran sample of the original purple wheat PWB, was plotted on column plots of the first three components with substantially different score values from YWB1, the bran sample of the yellow wheat that was developed together with PWB from the cross cultivation (Figure 3A). PWB was also inversely correlated with YWB2, the bran sample of certified yellow wheat in the market (“Keumkang”) in components one and three. The distinct distribution of PWB from YWB1, and YWB2 was also observed in the data matrix of the heat map (Figure 4). The contribution scores plots of the PCA model were introduced to determine how the variables affected this distribution (Figure 3B). Most of the variables, including 11 amino acids (Ala, Asn, Asp, Glu, Gln, Ile, Leu, Pro, Glp, Thr, and Val) showed a positive contribution. The examination of antioxidant capacity and phenolic content has been of great importance for evaluation in the development of colored grains (4). However, in the present analysis, the colored wheat was found to be an improved variety compared with the yellow wheat in terms of the primary metabolites, including the essential amino acids, which indicated that the investigation of the actual major primary metabolites is also important for evaluation in the development of colored grains. PWB was found to be different from YWB1 and YWB2, especially for Asn, Asp, Bt, Gln, Ile, Leu, and Val, which presented with dominant bars on the positive side. Lys was also attributed to the discrimination of PWB from YWB1, although a negative score indicated a relatively low content in PWB.

**Figure 5:**
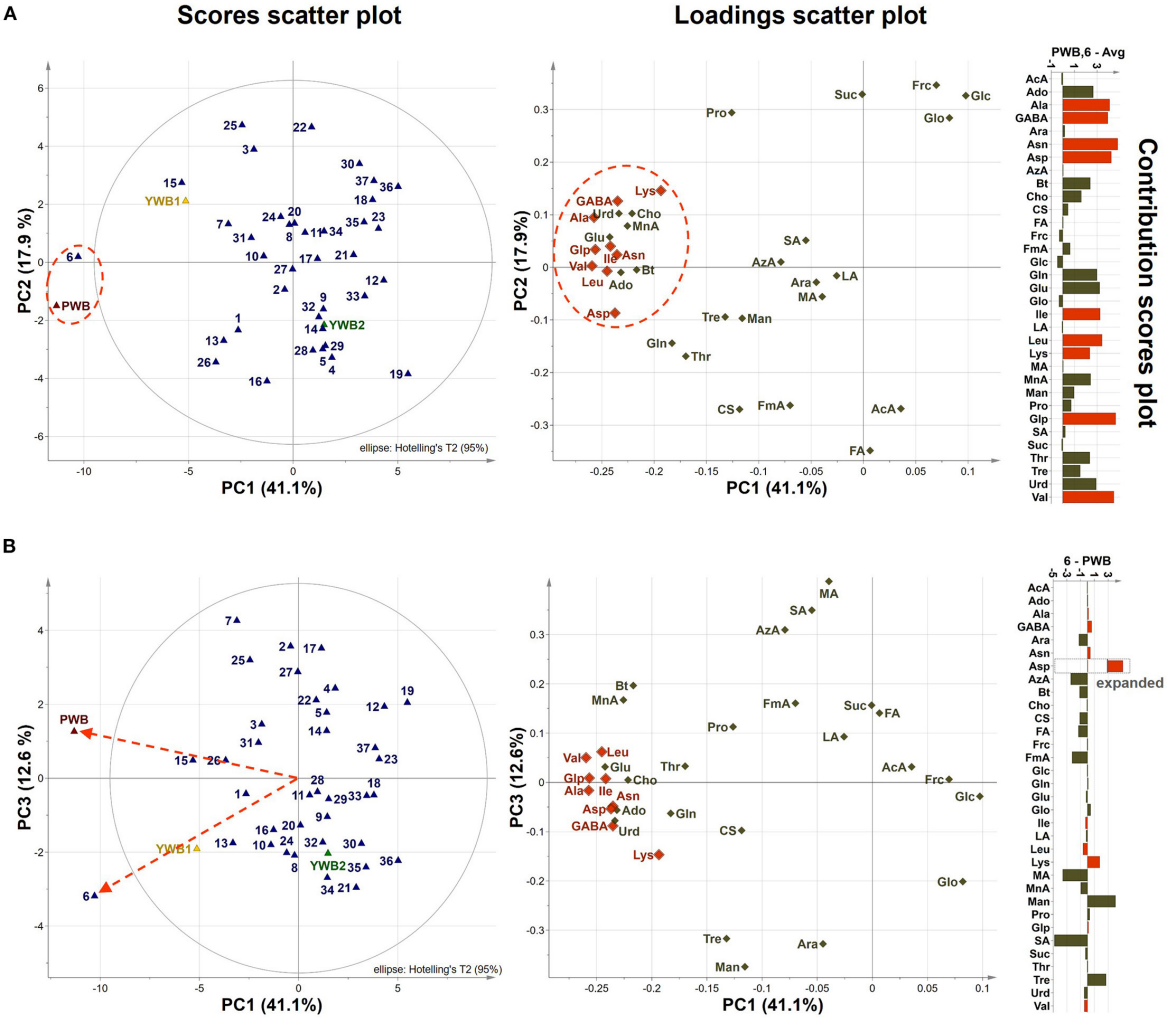
**(A)** PCA scores and loadings scatter plots of PC1 against PC2 (41.1 and 17.9% variation, respectively), contribution scores plot from average to (PWB, PWBr6) (p1 and p2 loadings were used as weights). **(B)** PCA scores and loadings scatter plots of PC1 against PC3 (41.1 and 12.6% variation, respectively), and contribution scores plot from PWBr6 to PWB (p1 and p3 loadings were used as weights). Variables outside three-fold standard deviation ranges are highlighted in orange.

**Figure 6:**
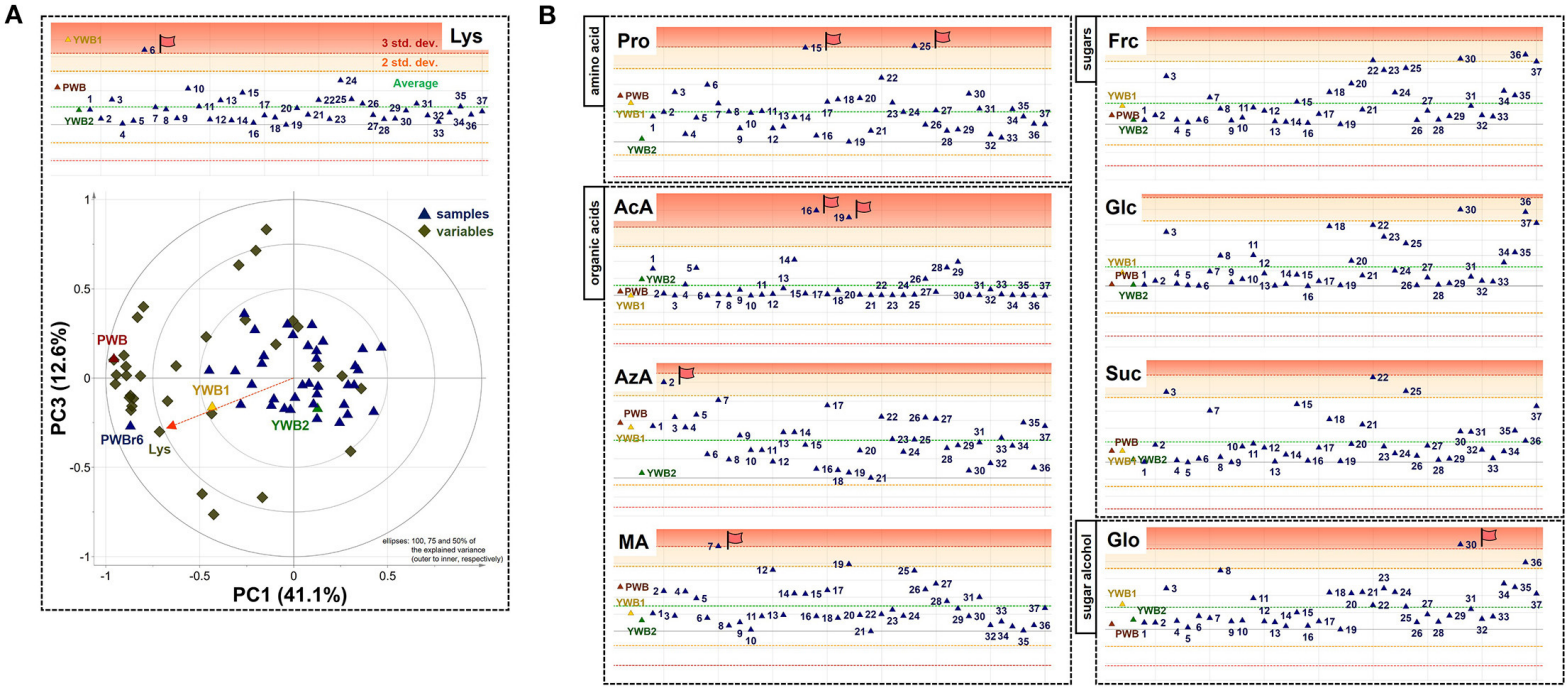
**(A)** Xvar plot of lysine (Lys) (top) and biplot of PC1 against PC3 (bottom). **(B)** Xvar plots of proline (Pro), acetate (AcA), azelate (AzA), malate (MA) (left, top to bottom), fructose (Frc), glucose (Glc), sucrose (Suc), and glycerol (Glo) (right, top to bottom).

On the scores scatter plots, PWB and one of the mutant lines, PWBr6, were detected as outliers at 95% confidence because of the substantial differences in PC1 scores (Figure 5). The heat map visualization helped to confirm the similarity between PWB and PWBr6 out of the 40 samples (Figure 4). Most of the variables were negatively weighted in component one toward the same side as PWB and PWBr6 plotted on the PCA scatter plots (Figure 5). This trend implies that PWB and PWBr6 are quantitatively stronger for the variables presented in the same part of the matrix. The outlying mutant line PWBr6 was further analyzed with a focus on compositional improvement over the original PWB. The scores scatter plot of PC1 against PC3 showed a relatively distinct distribution between PWB and PWBr6 (Figure 5B). PWBr6 exhibited a higher content of 13 metabolites (Ado, Ala, GABA, Asn, Asp, Gln, Glo, Lys, Man, Pro, Glp, Thr, and Tre) than PWB, of which 10 metabolites (Ado, Ala, GABA, Asn, Asp, Gln, Man, Glp, Thr, and Tre) showed the highest content in PWBr6 out of 40 samples (Supplementary Table 3). This observation suggested that PWBr6 was an improved culture line compared to the original PWB for the 13 metabolites. In particular, PWBr6 contained significantly more Lys than PWB, compensating for the low Lys content in the original purple wheat sample (Figure 6A). Lys is an essential amino acid, the adequate intake of which, is important for efficient calcium metabolism in the body (49). The Lys content in PWBr6 was similar to that in YWB1, which was the highest among the 40 samples. The compositional improvement observed in PWB and PWBr6 was more pronounced when compared with the market sample YWB2 (Supplementary Figure 16).

The other 36 mutant lines were projected onto the plots within the 95% confidence interval. This revealed that radiation treatment of the original purple wheat induced various mutations in terms of chemical composition. The XVar plots were generated and interpreted for each metabolite to identify the mutant lines that showed compositional improvement for specific metabolites (Figure 6B). The analyses led to the achievement of significant findings for the following eight metabolites: one amino acid (Pro), three organic acids (AcA, AzA, and MA), three sugars (Frc, Glc, and Suc), and one sugar alcohol (Glo). Two mutant lines, PWBr15 and PWBr25, were obtained with a substantial enhancement of Pro content compared to the original PWB. Pro constitutes a significant portion of the collagen proteins and is involved in the physical stabilization of connective tissues and molecular signaling (50–52). AcA was found to be highly abundant in PWBr16, as well as PWBr19, which was presented as one of the three representatives in Figure 1. AcA is the primary ingredient in vinegar. It has been attracting attention as a food ingredient because of its preventive and therapeutic effects on metabolic syndromes such as obesity, diabetes, and hypertension (53–55). The content of another organic acid, AzA, was higher in PWBr2, which suggested that this mutant line might be used in the development of anti-acne natural cosmetics due to the antibacterial effect of AzA (56). PWBr7 is another resource for natural remedies because MA, a rich component of PWBr7, has been proven to be beneficial in the treatment of dry mouth (57). The content of low-molecular-weight sugars, including Frc, Glc, and Suc, has been reported as an important factor in the manufacturing control of bread quality, as sugars are the main substrates for yeast fermentation (58). As described in Figure 1, some of the mutant lines, including PWBr3, showed more intense ^1^H NMR signals of the sugars. This phenomenon was also observed in the Xvar plots of Frc, Glc, and Suc (Figure 6B), indicating that radiation treatment also induced favorable mutations in the bakery industry. A higher content of Glo was found in the mutant line PWBr30, and this might highlight the mutant line as a good candidate for application as a natural cosmetic resource because Glo has been used for skin repair as it enhances the epidermal barrier function (59). The results of the NMR-based metabolomics analysis demonstrated the significant radiation-induced mutagenic effects as well as the methodological efficacy of the multivariate analysis, followed by the Chenomx-aided identification and quantification of the polar metabolites for the development of nutritionally improved mutant lines.

## Conclusion

The introduction of NMR spectroscopy addressed the pre-existing issue of the conventional C_18_ LC system in the analysis of polar compounds, enabling efficient identification and quantification of polar metabolites in bran. Although conventional phytochemical studies have mainly focused on secondary metabolites with a certain degree of polarity, polar primary metabolites are worth studying in terms of their content predominance and functional values. The peak shape-based assignments through all the distinguishable ^1^H NMR signals corresponding to each metabolite were proven to be efficient and reliable for the NMR-based metabolomics analysis in the present study, to provide the annotation of 33 metabolites: three choline derivatives (betaine, choline, and choline sulfate), three sugar alcohols (arabinitol, glycerol, and mannitol), four sugars (fructose, glucose, sucrose, and trehalose), 13 amino acids (alanine, γ-aminobutyrate, asparagine, asparatate, glutamate, glutamine, isoleucine, leucine, lysine, proline, pyroglutamate, threonine, and valine), eight organic acids (acetate, azelate, formate, fumarate, lactate, malate, malonate, and succinate), and two nucleosides (adenosine and uridine). PCA and HCA statistical studies revealed differences within samples. The colored wheat (original, non-irradiated) was found to be an improved variety compared to yellow wheat in terms of primary metabolites including essential amino acids, emphasizing the importance of investigating the actual major primary metabolites in the development of colored grains. The XVar plot of each metabolite was shown to be an effective statistical tool that was utilized to determine significant compositional improvement in the radiation-induced mutant samples compared to the original. The following nine metabolites were observed to present with higher content in the mutant lines: two amino acids (lysine and proline), three organic acids (aceate, azelate, and malate), three sugars (fructose, glucose, and sucrose), and one sugar alcohol (glycerol). The analysis platform developed in the present study is expected to be used in the evaluation of additional radiation-induced mutations in the future.

## Data Availability Statement

The raw data supporting the conclusions of this article will be made available by the authors, without undue reservation.

## Author Contributions

Y-SK carried out the data acquisition and analysis and drafted the manuscript. A-RH, M-JH, and J-BK participated in material culture. J-WN participated in conceptualization and supervision of the study. P-HP and HC helped to draft the manuscript. All authors read and approved the final manuscript.

## Funding

This research was supported by Brain Pool Program (NRF-2019H1D3A1A01102673) and the Radiation Technology R&D Program (NRF-2017M2A2A6A05018541) funded by the Ministry of Science and ICT through the National Research Foundation of Korea.

## Conflict of Interest

The authors declare that the research was conducted in the absence of any commercial or financial relationships that could be construed as a potential conflict of interest.

## Publisher's Note

All claims expressed in this article are solely those of the authors and do not necessarily represent those of their affiliated organizations, or those of the publisher, the editors and the reviewers. Any product that may be evaluated in this article, or claim that may be made by its manufacturer, is not guaranteed or endorsed by the publisher.
