# Corrigendum: ^1^H NMR-Based Chemometrics to Gain Insights Into the Bran of Radiation-Induced Colored Wheat Mutant

**DOI:** 10.3389/fnut.2022.950505

**Published:** 2022-06-23

**Authors:** Yun-Seo Kil, Ah-Reum Han, Min-Jeong Hong, Jin-Baek Kim, Pil-Hoon Park, Hyukjae Choi, Joo-Won Nam

**Affiliations:** ^1^College of Pharmacy, Yeungnam University, Gyeongsan-si, South Korea; ^2^Advanced Radiation Technology Institute, Korea Atomic Energy Research Institute, Jeongeup-si, South Korea; ^3^Research Institute of Cell Culture, Yeungnam University, Gyeongsan-si, South Korea

**Keywords:** colored wheat bran, radiation, nuclear magnetic resonance, chemometrics, polar metabolites

In the original article, there was a mistake in Figure 2 as published. The peak annotations in Figure 2C should be “H_2_-1 of CS” and “N(C*H*_3_)_3_ of CS” instead of “H-2 of CS” and “NC*H*_3_ of CS”, respectively. The corrected Figure 2 appears below.

**Figure 2:**
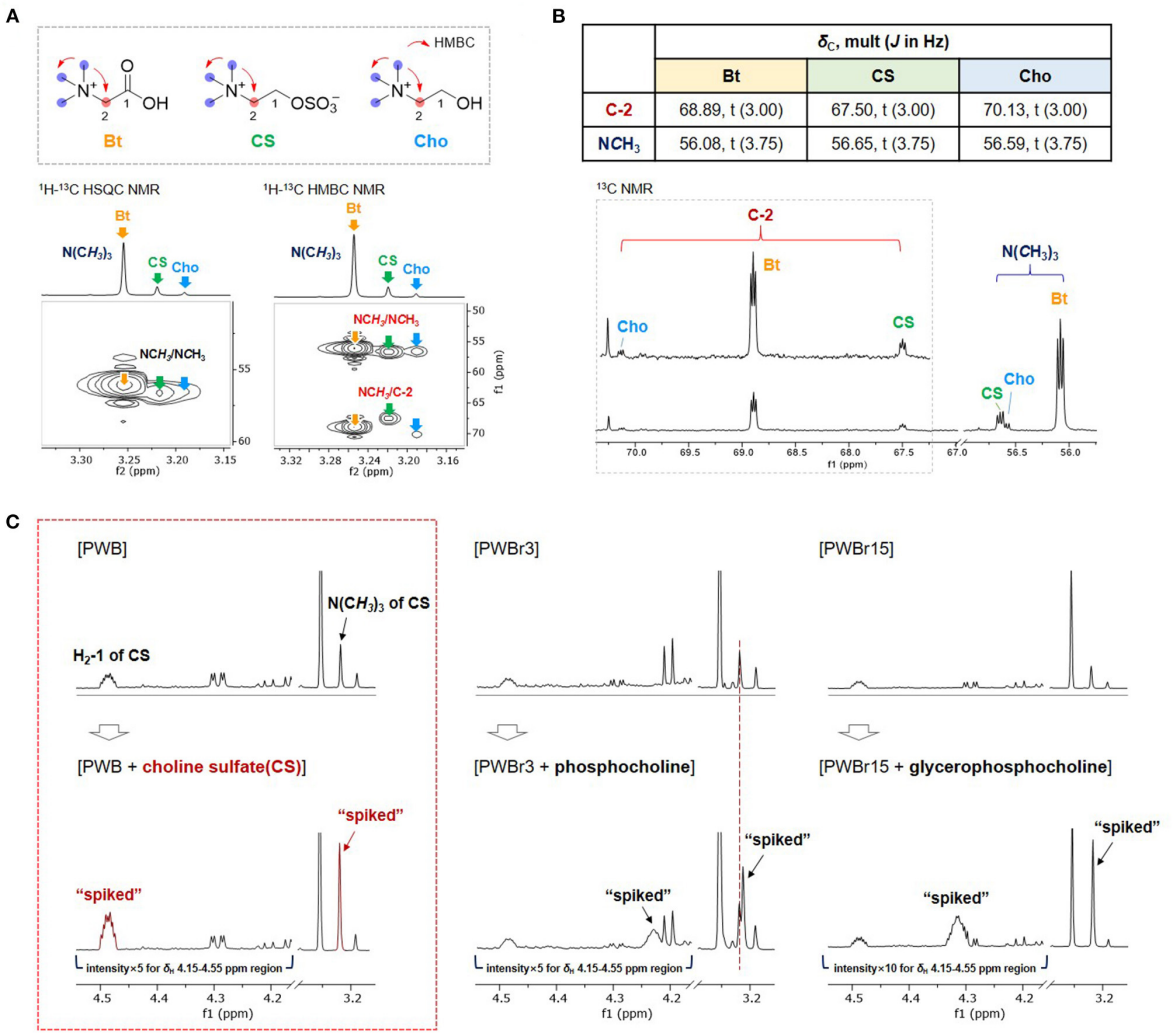
Comparison of **(A)** ^1^H-^13^C HSQC and HMBC correlations and **(B)** ^13^C NMR signals of C-2 and N*C*H_3_ of betaine (Bt), choline sulfate (CS), and choline (Cho) in PWB. **(C)** Spiking ^1^H NMR experiments with commercial standards of CS, phosphocholine, and glycerophosphocholine.

The authors apologize for this error and state that this does not change the scientific conclusions of the article in any way. The original article has been updated.

## Publisher's Note

All claims expressed in this article are solely those of the authors and do not necessarily represent those of their affiliated organizations, or those of the publisher, the editors and the reviewers. Any product that may be evaluated in this article, or claim that may be made by its manufacturer, is not guaranteed or endorsed by the publisher.

