# Supplementary material for: 1H NMR-Based Chemometrics to Gain Insights Into the Bran of Radiation-Induced Colored Wheat Mutant
Source: Front Nutr. 2022 Jan 4;8:806744. doi: 10.3389/fnut.2021.806744 (PMC8764193; doi:10.3389/fnut.2021.806744)
Supplement: Supplementary file 1 [file Data_Sheet_1.pdf]

frontiers

# *Supplementary material*

# $^{1}$H NMR-Based Chemometrics to Gain Insights into the Bran of Radiation-Induced Colored Wheat Mutant

***Yun-Seo Kil[1], Ah-Reum Han[2], Min-Jeong Hong[2], Jin-Baek Kim[2], Pil-Hoon Park[1,3], Hyukjae Choi[1,3], Joo-Won Nam[1*]***

*[1]College of Pharmacy, Yeungnam University, Gyeongsan-si, Gyeongsangbuk-do, South Korea, [2]Advanced Radiation Technology Institute, Korea Atomic Energy Research Institute, Jeongeup-si, Jeollabuk-do, South Korea, [3]Research Institute of Cell Culture, Yeungnam University, Gyeongsan-si, Gyeongsangbuk-do, South Korea*

***Correspondence:** *Joo-Won Nam*
*jwnam@yu.ac.kr*

# Table of Contents


| | | |
|---|---|---|
| **Figure 1.** | Preliminary data of **PWBr18**: (A) comparison of HPLC analysis data under detection of ELSD and PDA (254 nm), and (B) comparison of $^1$H NMR spectra in $D_2O$ and $CD_3OD$ | S3 |
| **Figure 2.** | $^1$H NMR ($D_2O$) spectra of 40 wheat bran samples | S4 |
| **Table 1.** | $^1$H and $^{13}$C NMR data of the identified metabolites with observed $^1$H-$^1$H COSY and $^1$H-$^{13}$C HMBC correlations in three representatives, **PWB**, **PWBr3**, or **PWBr19** | S5 |
| **Table 2.** | BMRD codes of the identified metabolites | S15 |
| **Figure 3.** | Detailed 2D NMR ($D_2O$) data analysis of arabinitol (Ara) in **PWB** | S16 |
| **Figure 4.** | Detailed 2D NMR ($D_2O$) data analysis of glycerol (Glo) in **PWB** | S17 |
| **Figure 5.** | Detailed 2D NMR ($D_2O$) data analysis of mannitol (Man) in **PWB** | S18 |
| **Figure 6.** | $^{13}$C NMR data ($D_2O$) of **PWBr3** with BMRD reference data of fructose and glucose | S19 |
| **Figure 7.** | Stacked $^1$H NMR ($D_2O$) spectra in an expansion for malate (MA) signals | S20 |
| **Figure 8.** | Detailed 2D NMR ($D_2O$) data analysis of malate (MA) in **PWB** | S21 |
| **Figure 9.** | Key $^1$H-$^1$H COSY ($D_2O$) correlations of malate in **PWB**, **PWBr14**, and **PWBr19** | S22 |
| **Figure 10.** | Spiking $^1$H NMR experiments ($D_2O$) with commercial standards of azelate and sebacate | S23 |
| **Figure 11.** | Detailed 2D NMR ($D_2O$) data analysis of betaine (Bt) in **PWB** | S24 |
| **Figure 12.** | Detailed 2D NMR ($D_2O$) data analysis of choline sulfate (CS) in **PWB** | S25 |
| **Figure 13.** | Detailed 2D NMR ($D_2O$) data analysis of choline (Cho) in **PWB** | S26 |
| **Figure 14.** | Preparation of the custom compound library for choline sulfate | S27 |
| **Figure 15.** | Key $^1$H-$^1$H COSY ($D_2O$) correlations for identification of (A) lactate (LA) and threonine (Thr), and (B) valine (Val), leucine (Leu), and isoleucine (Ile) in **PWB** | S28 |
| **Table 3.** | Concentrations (mg/g dried extract) of the identified metabolites of the wheat bran samples | S29 |
| **Figure 16.** | Subtraction plots of (A) **PWB** with **YWB1** or **YWB2**, and (B) **PWBr6** with **PWB**, **YWB1**, or **YWB2** | S30 |


**Spectroscopic Data**


| | | |
|---|---|---|
| **Figures 17-20.** | 1D and 2D NMR data ($D_2O$) of **PWB** | S31 |
| **Figures 21-24.** | 1D and 2D NMR data ($D_2O$) of **PWBr3** | S35 |
| **Figures 25-28.** | 1D and 2D NMR data ($D_2O$) of **PWBr19** | S39 |
| **Figures 29-33.** | 1D and 2D NMR data ($D_2O$) of choline sulfate (CS) | S43 |

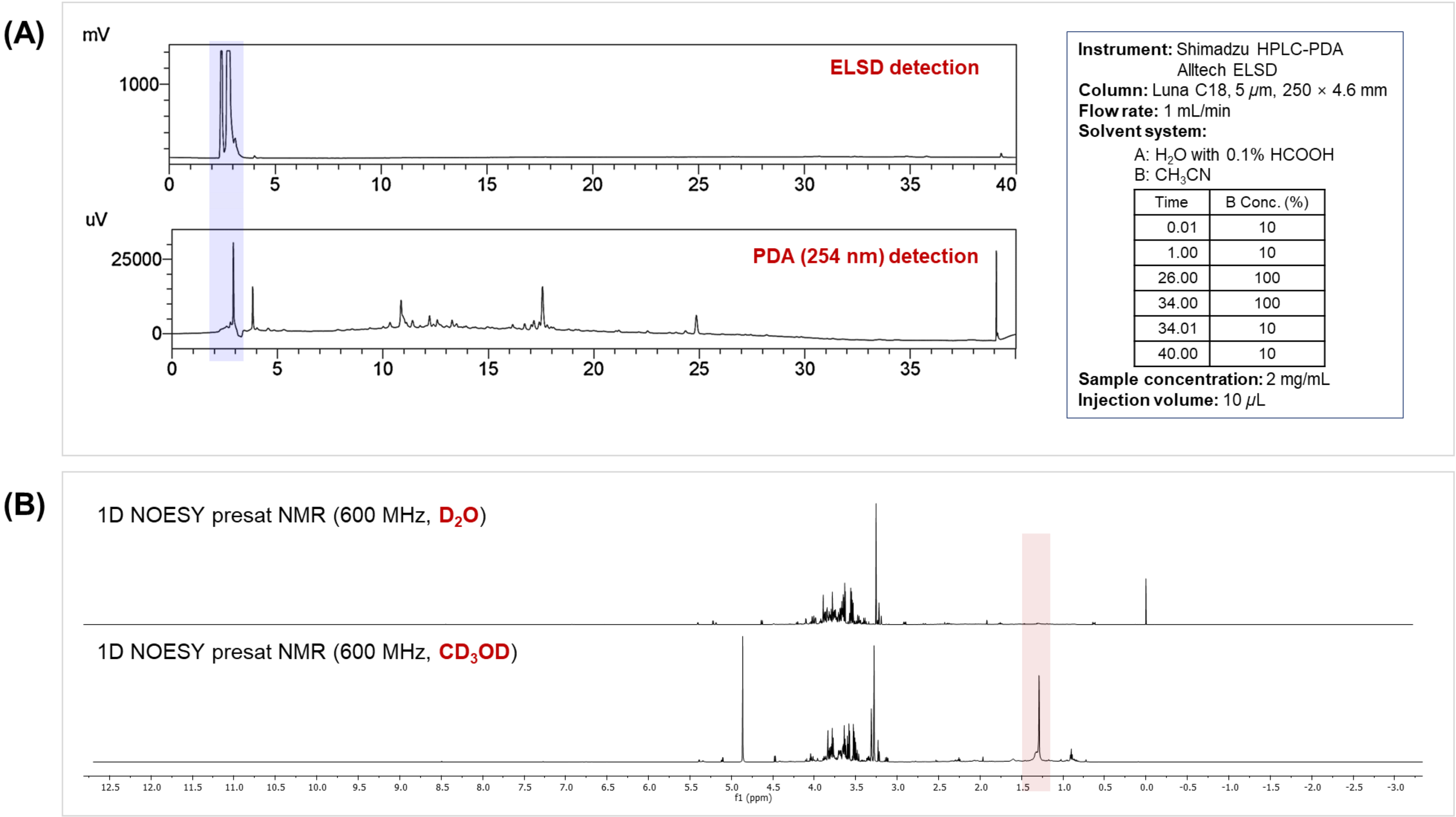


**Supplementary Figure 1.** Preliminary data of **PWBr18**: (A) comparison of HPLC analysis data under detection of ELSD and PDA (254 nm), and (B) comparison of $^{1}H$ NMR (600 MHz) spectra in $D_2O$ and $CD_3OD$.

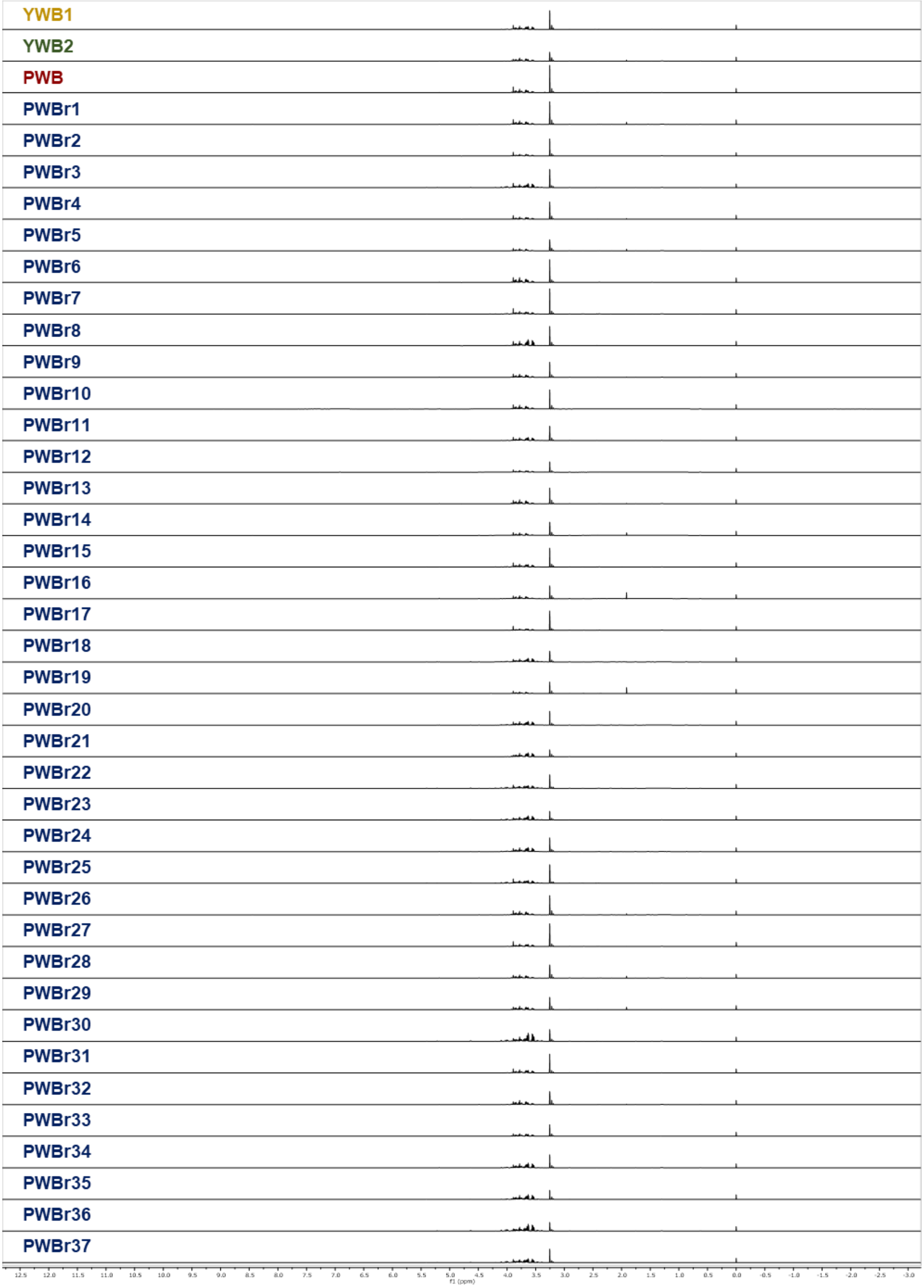


**Supplementary Figure 2.** $^1H$ NMR (600 MHz, $D_2O$) spectra of 40 wheat bran samples (1D NOESY presat pulse sequence with $\delta_H$ 4.70 of a presaturation frequency for water suppression)

**Supplementary Table 1.** $^{1}$H (600 MHz) and $^{13}$C NMR (150 MHz) data of the identified metabolites with $^{1}$H-$^{1}$H COSY (**blue bold**) and $^{1}$H-$^{13}$C HMBC (→) correlations in three representatives, **PWB**, **PWBr3**, or **PWBr19**.

**(NOTE)** This table includes the assignments of $^{1}$H and $^{13}$C NMR resonances supported by 2D NMR correlations, with aids of the BMRD NMR data base. The $^{1}$H NMR peak assignments, performed only by the peak shape-based assessment using compound information from the Chenomx-embedded library (i.e. no sufficient evidences from 2D NMR correlation), were labeled "LB" in a meaning of "library based". The $^{13}$C NMR assignment was still given when the $^{13}$C NMR signal was significantly detectable in the $^{13}$C NMR data of the extract samples and unambiguous assignment was allowed in comparison with the BMRD reference or previously reported data. "ND" (not determined) indicates the case where the $^{13}$C NMR signal was not determined due to low signal intensity or signal overlap. The 2D NMR correlations with substantial signal overlap, were not included due to uncertainty in the assignment.

| metabolite (ID) | position | $\delta_H$, mult (*J* in Hz) | $\delta_C$ | COSY (**blue bold**) | HMBC (H → C) | Reference sample code | MW (Da) |
|---|---|---|---|---|---|---|---|
| **Sugar Alcohols** | | | | | | | |
| 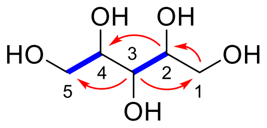  arabinitol (**Ara**) | 1 | 3.83, dd (12.0, 3.0), Ha<br>3.65$^{a}$, Hb | 65.7 | Ha/Hb | 2 | **PWB** | 152.15 |
| | 2 | 3.74$^{a}$ | 73.6 | 3 | 1, 3, 4 | | |
| | 3 | 3.56, dd (8.4, 2.2) | 73.2 | 2 | 1, 2, 4, 5 | | |
| | 4 | 3.92, ddd (7.4, 5.5, 2.2) | 73.0 | 5 | 5 | | |
| | 5 | 3.66$^{a}$ | 65.8 | 4 | | | |
| 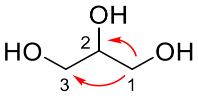  glycerol (**Glo**) | 1 | 3.64, dd (11.6, 4.7), Ha<br>3.55, dd (11.6, 6.5), Hb | 65.2 | Ha/Hb | 2, 3 | **PWB** | 104.17 |
| | 2 | 3.77$^{a}$ | 74.8 | | | | |
| | 3 | 3.64, dd (11.6, 4.7), Ha<br>3.55, dd (11.6, 6.5), Hb | 65.2 | Ha/Hb | 1, 2 | | |
| 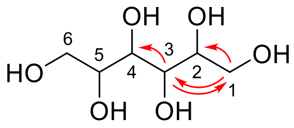  mannitol (**Man**) | 1 | 3.86, dd (11.8, 2.8), Ha<br>3.66, dd (11.8, 6.2), Hb | 65.9 | Ha/Hb | 2, 3 | **PWB** | 182.17 |
| | 2 | 3.75, ddd (8.5, 6.2, 2.8) | 73.5 | | 1, 4 | | |
| | 3 | 3.79, d (8.5) | 72.0 | | 1, 4, 5 | | |
| | 4 | 3.79, d (8.5) | 72.0 | | 2, 3, 6 | | |
| | 5 | 3.75, ddd (8.5, 6.2, 2.8) | 73.5 | | 3, 6 | | |
| | 6 | 3.86, dd (11.8, 2.8), Ha<br>3.66, dd (11.8, 6.2), Hb | 65.9 | Ha/Hb | 4, 5 | | |

$^{a}$Multiplicity was not determined due to signal overlap.

(**Supplementary Table 1.** continued)

| metabolite (ID) | position | $\delta_H$, mult (*J* in Hz) | $\delta_C$ | COSY (**blue bold**) | HMBC (H → C) | Reference sample code | MW (Da) |
|---|---|---|---|---|---|---|---|
| **Sugars** | | | | | | | |
| fructose (**Frc**) | | | | | | | |
| 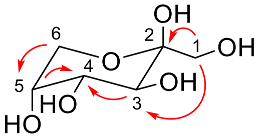 | 1 | 3.70, d (11.8), Ha | 66.6 | Ha/Hb | 2, 3 | | |
| | | 3.55, d (11.8), Hb | | | | | |
| | 2 | | 100.8 | | | | |
| | 3 | 3.78, d (10.0) | 70.3 | | | | |
| | 4 | 3.88, dd (10.0, 3.5) | 72.4 | | 3 | | |
| | 5 | 3.98, dt (3.5, 1.3) | 71.9 | | 3, 4, 6 | | |
| *β*-fructopyranose | 6 | 4.01, dd (12.7, 1.3), Ha | 66.1 | Ha/Hb | 2, 5 | | |
| | | 3.69, dd (12.7, 1.3), Hb | | | | **PWBr3** | 180.16 |
| 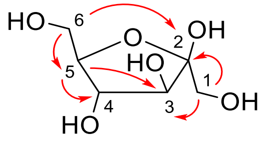 | 1 | 3.58, d (12.1), Ha | 65.4 | | 2, 3 | | |
| | | 3.54[a], Hb | | | | | |
| | 2 | | 104.2 | | | | |
| | 3 | 4.10[a] | 78.1 | | 4 | | |
| | 4 | 4.10[a] | 77.2 | | 3 | | |
| | 5 | 3.82[a] | 83.4 | | 4 | | |
| *β*-fructofuranose | 6 | 3.80[a], Ha | 65.1 | | 5 (only from Hb) | | |
| | | 3.66[a], Hb | | | | | |
| | **Glc** | | | | | | |
| | 1 | 5.40, d (3.9) | 94.9 | 2 | 3, 5, 2' | | |
| | 2 | 3.55[a] | 73.8 | 1 | | | |
| 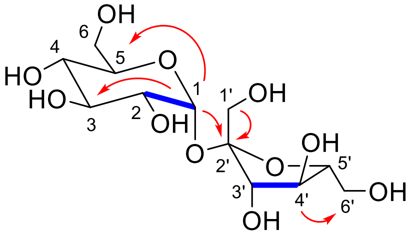 | 3 | LB | 75.3 | | | | |
| | 4 | LB | ND | | | | |
| | 5 | LB | 75.1 | | | | |
| | 6 | LB | 62.8 | | | | |
| | **Frc** | | | | | **PWBr3** | 342.30 |
| | 1' | 3.67[a] | 64.0 | | 2' | | |
| | 2' | | 106.4 | | | | |
| | 3' | 4.21, d (8.8) | 79.1 | 4' | 4' | | |
| sucrose (**Suc**) | 4' | 4.04, t (8.6) | 76.7 | 3' | 3', 6' | | |
| | 5' | 3.88[a] | 84.1 | | | | |
| | 6' | LB | 65.1 | | | | |

[a]Multiplicity was not determined due to signal overlap.

(**Supplementary Table 1.** continued)

| metabolite (ID) | position | $\delta_H$, mult (*J* in Hz) | $\delta_C$ | COSY (**blue bold**) | HMBC (H → C) | Reference sample code | MW (Da) |
|---|---|---|---|---|---|---|---|
| **Sugars** | | | | | | | |
| glucose (**Glc**) 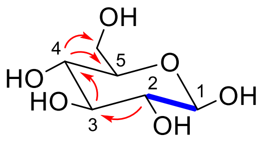 *β*-glucopyranose | 1 | 4.63, d (8.0) | 98.6 | 2 | | **PWBr3** | 180.16 |
| | 2 | 3.23, dd (9.4, 8.0) | 76.8 | 1 | 1, 3 | | |
| | 3 | 3.48, t (9.4) | 78.5 | | 2, 4 | | |
| | 4 | 3.39, t (9.4) | 72.3 | | 5, 6 | | |
| | 5 | LB | 78.7 | | | | |
| | 6 | LB | 63.5 | | | | |
| 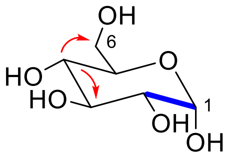 *α*-glucopyranose | 1 | 5.22, d (3.8) | 94.8 | 2 | | | |
| | 2 | 3.52[a] | 74.2 | 1 | | | |
| | 3 | LB | 75.5 | | | | |
| | 4 | 3.40, t (9.6) | 72.4 | | 3, 6 | | |
| | 5 | LB | 74.2 | | | | |
| | 6 | LB | 63.3 | | | | |
| 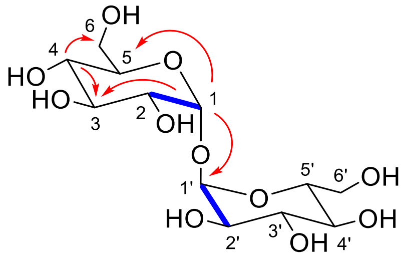 trehalose (**Tre**) | 1 | 5.18, d (3.8) | 95.9 | 2 | 3, 5, 1' | **PWB** | 342.30 |
| | 2 | 3.64[a] | 73.8 | 1 | | | |
| | 3 | LB | 75.2 | | | | |
| | 4 | 3.44, t (9.7) | 72.4 | | 3, 6 | | |
| | 5 | LB | 74.9 | | | | |
| | 6 | 3.84, 3.75[a] | 63.2 | | | | |
| | 1' | 5.18, d (3.8) | 95.9 | 2' | 1, 3', 5' | | |
| | 2' | 3.64[a] | 73.8 | 1' | | | |
| | 3' | LB | 75.2 | | | | |
| | 4' | 3.44, t (9.7) | 72.4 | | 3', 6' | | |
| | 5' | LB | 74.9 | | | | |
| | 6' | 3.84, 3.75[a] | 63.2 | | | | |

[a]Multiplicity was not determined due to signal overlap.

(**Supplementary Table 1.** continued)

| metabolite (ID) | position | $\delta_H$, mult ($J$ in Hz) | $\delta_C$, mult ($J$ in Hz) | COSY (**blue bold**) | HMBC (H → C) | Reference sample code | MW (Da) |
|---|---|---|---|---|---|---|---|
| **Choline derivatives** | | | | | | | |
| 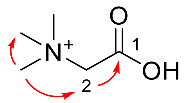 betaine (**Bt**) | 1 | - | 172.0 | | | **PWB** | 117.15 |
| | 2 | 3.89, s | 68.89, t (3.00) | | 1, NCH$_3$ | | |
| | N(CH$_3$)$_3$ | 3.25, s | 56.08, t (3.75) | | 2, NCH$_3$ | | |
| 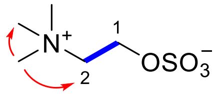 choline sulfate (**CS**) | 1 | 4.49, m | 64.7 | 2 | | **PWB** | 183.23 |
| | 2 | 3.75[a] | 67.50, t (3.00) | 1 | NCH$_3$ | | |
| | N(CH$_3$)$_3$ | 3.22, s | 56.65, t (3.75) | | 2, NCH$_3$ | | |
| 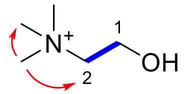 choline (**Cho**) | 1 | 4.05[a] | 58.3 | 2 | | **PWB** | 104.17 |
| | 2 | 3.51, m | 70.13, t (3.00) | 1 | NCH$_3$ | | |
| | N(CH$_3$)$_3$ | 3.19, s | 56.59, t (3.75) | | 2, NCH$_3$ | | |

[a]Multiplicity was not determined due to signal overlap.

(**Supplementary Table 1.** continued)

| metabolite (ID) | position | $\delta_H$, mult (*J* in Hz) | $\delta_C$ | COSY (**blue bold**) | HMBC (H → C) | Reference sample code | MW (Da) |
|---|---|---|---|---|---|---|---|
| **Nucleosides** | | | | | | | |
| 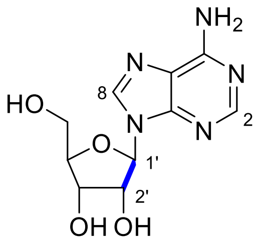 adenosine (**Ado**) | 2 | LB | ND | | | | |
| | 8 | LB | ND | | | | |
| | 1' | 6.06 (d, $J = 6.0$ Hz) | ND | 2' | | | |
| | 2' | 4.79 | ND | | | | |
| | 3' | LB | ND | | | **PWB** | 267.24 |
| | 4' | LB | ND | | | | |
| | 5' | LB | ND | | | | |
| 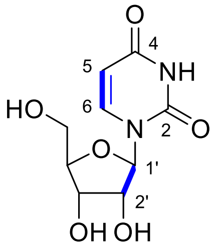 uridine (**Urd**) | 5 | 5.89, d (8.0) | ND | 6 | | | |
| | 6 | 7.86, d (8.1) | ND | 5 | | | |
| | 1' | 5.90, d (4.5) | ND | 2' | | | |
| | 2' | 4.34[a] | ND | 1' | | | |
| | 3' | LB | ND | | | **PWB** | 244.20 |
| | 4' | LB | ND | | | | |
| | 5' | LB | ND | | | | |

[a]Multiplicity was not determined due to signal overlap.

(**Supplementary Table 1.** continued)

| metabolite (ID) | position | $\delta_H$, mult (*J* in Hz) | $\delta_C$ | COSY (**blue bold**) | HMBC (H → C) | Reference sample code | MW (Da) |
|---|---|---|---|---|---|---|---|
| **Organic acids** | | | | | | | |
| 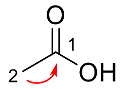 acetate (**AcA**) | 1 | | 184.0 | | | **PWBr19** | 60.05 |
| | 2 | 1.91, s | 25.8 | | 1 | | |
| 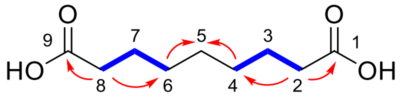 azelate (**AzA**) | 1 | | 186.5 | | | **PWB** | 188.22 |
| | 2 | 2.18, t (7.5) | 40.0 | 3 | 1, 3, 4 | | |
| | 3 | 1.54[a] | 28.4 | 2, 4 | 4 | | |
| | 4 | 1.30[a] | 31.3 | 3 | 5 | | |
| | 5 | 1.30[a] | 31.0 | | | | |
| | 6 | 1.30[a] | 31.3 | 7 | | | |
| | 7 | 1.54[a] | 28.4 | 6, 8 | | | |
| | 8 | 2.18, t (7.5) | 40.0 | 7 | 6, 7, 9 | | |
| | 9 | | 186.5 | | | | |
| 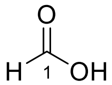 formate (**FA**) | 1 | 8.44, s | 173.8 | | | **PWB** | 46.03 |
| 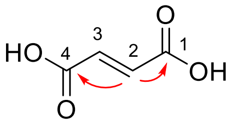 fumarate (**FmA**) | 1 | | 177.4 | | | **PWB** | 116.07 |
| | 2 | 6.51, s | 138.0 | 1, 4 | | | |
| | 3 | 6.51, s | 138.0 | 1, 4 | | | |
| | 4 | | 177.4 | | | | |

[a]Multiplicity was not determined due to signal overlap.

(**Supplementary Table 1.** continued)

| metabolite (ID) | position | $\delta_H$, mult ($J$ in Hz) | $\delta_C$ | COSY (**blue bold**) | HMBC (H → C) | Reference sample code | MW (Da) |
|---|---|---|---|---|---|---|---|
| **Organic acids** | | | | | | | |
| 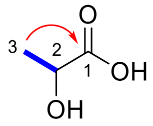 | 1 | | 185.3[b] | | | | |
| | 2 | 4.10[a] | 71.2 | 3 | | | |
| lactate (**LA**) | 3 | 1.32, d (7.0) | 22.8 | 2 | 1 | **PWB** | 90.08 |
| 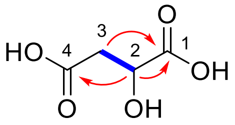 | 1 | | 182.2 | | | | |
| | 2 | 4.29, dd (10.0, 3.1) | 73.0 | 3 | 1, 3, 4 | | |
| | 3 | 2.67, dd (15.5, 3.1), Ha<br>2.38, dd (15.5. 10.0), Hb | 45.1 | 2, Ha/Hb | 1, 2 | **PWB** | 134.09 |
| malate (**MA**) | 4 | | 183.6 | | | | |
| 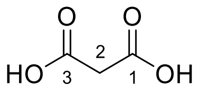 | 1 | | ND | | | | |
| | 2 | 3.13, s | 51.0[b] | | | **PWB** | 104.06 |
| malonate (**MnA**) | 3 | | ND | | | | |
| 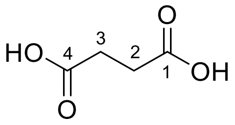 | 1 | | ND | | | | |
| | 2 | 2.42, s | 36.3 | | | | |
| | 3 | 2.42, s | 36.3 | | | **PWBr19** | 118.09 |
| succinate (**SA**) | 4 | | ND | | | | |

[a]Multiplicity was not determined due to signal overlap. [b]chemical shift was extracted from HMBC correlation.

(**Supplementary Table 1.** continued)

| metabolite (ID) | position | $\delta_H$, mult ($J$ in Hz) | $\delta_C$ | COSY (**blue bold**) | HMBC (H → C) | Reference sample code | MW (Da) |
|---|---|---|---|---|---|---|---|
| **Amino acids** | | | | | | | |
| 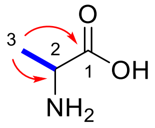 | 1 | | 178.5 | | | | |
| | 2 | 3.77[a] | 53.2 | 3 | | | |
| | 3 | 1.47, d (7.3) | 18.9 | 2 | 1, 2 | **PWB** | 89.09 |
| alanine (**Ala**) | | | | | | | |
| 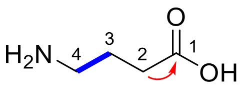 | 1 | | 184.2[b] | | | | |
| | 2 | 2.29, t (7.4) | 37.0 | 3 | 1 | **PWB** | 103.12 |
| | 3 | 1.89[a] | 26.3 | 2, 4 | | | |
| γ-aminobutyrate (**GABA**) | 4 | 3.00, br t (7.6) | 42.0 | 3 | | | |
| 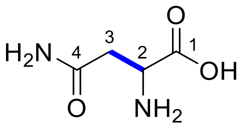 | 1 | | ND | | | | |
| | 2 | 4.00[a] | ND | 3 | | | |
| | 3 | 2.94, dd (16.9, 4.2), Ha<br>2.85, dd (16.9, 7.8), Hb | ND | 2 | | **PWB** | 132.12 |
| | 4 | | ND | | | | |
| asparagine (**Asn**) | | | | | | | |
| 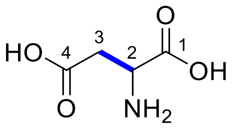 | 1 | | ND | | | | |
| | 2 | 3.90[a] | ND | 3 | | | |
| | 3 | 2.80, dd (17.5, 3.8), Ha<br>2.67[a], Hb | ND | 2, Ha/Hb | | **PWB** | 133.10 |
| | 4 | | ND | | | | |
| aspartate (**Asp**) | | | | | | | |

[a]Multiplicity was not determined due to signal overlap. [b]chemical shift was extracted from HMBC correlation.

(**Supplementary Table 1.** continued)

| metabolite (ID) | position | $\delta_H$, mult (*J* in Hz) | $\delta_C$ | COSY (**blue bold**) | HMBC (H → C) | Reference sample code | MW (Da) |
|---|---|---|---|---|---|---|---|
| **Amino acids** | | | | | | | |
| 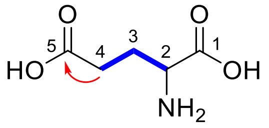 glutamate (**Glu**) | 1 | | ND | | | | |
| | 2 | 3.75[a] | 57.3 | 3 | | | |
| | 3 | 2.11, 2.06[a] | 29.6 | 2, 4 | | **PWB** | 147.13 |
| | 4 | 2.34[a] | 36.1 | 3 | 5 | | |
| | 5 | | 183.8[b] | | | | |
| 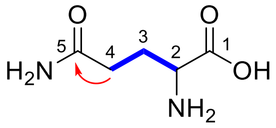 glutamine (**Gln**) | 1 | | ND | | | | |
| | 2 | 3.77[a] | 56.8 | 3 | | | |
| | 3 | 2.13[a] | 28.9 | 2, 4 | | **PWB** | 146.14 |
| | 4 | 2.45[a] | 33.5 | 3 | 5 | | |
| | 5 | | 180.4[b] | | | | |
| 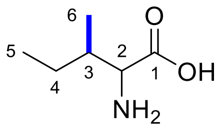 isoleucine (**Ile**) | 1 | | ND | | | | |
| | 2 | LB | ND | | | | |
| | 3 | 1.97[a] | ND | 6 | | **PWB** | 131.17 |
| | 4 | LB | ND | | | | |
| | 5 | LB | ND | | | | |
| | 6 | 1.00, d (7.0) | ND | 3 | | | |
| 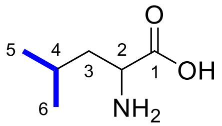 leucine (**Leu**) | 1 | | ND | | | | |
| | 2 | LB | ND | | | | |
| | 3 | LB | ND | | | **PWB** | 131.17 |
| | 4 | 1.71[a] | ND | 5, 6 | | | |
| | 5, 6 | 0.95[a] | ND | 4 | | | |

[a]Multiplicity was not determined due to signal overlap. [b]chemical shift was extracted from HMBC correlation.

(**Supplementary Table 1.** continued)

| metabolite (ID) | position | $\delta_H$, mult (*J* in Hz) | $\delta_C$ | COSY (**blue bold**) | HMBC (H → C) | Reference sample code | MW (Da) |
|---|---|---|---|---|---|---|---|
| **Amino acids** | | | | | | | |
| 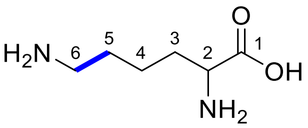 lysine (**Lys**) | 1 | LB | ND | | | **PWB** | 146.19 |
| | 2 | LB | ND | | | | |
| | 3 | LB | ND | | | | |
| | 4 | LB | ND | | | | |
| | 5 | 1.71[a] | ND | 6 | | | |
| | 6 | 3.01[a] | ND | 5 | | | |
| 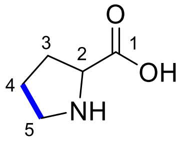 proline (**Pro**) | 1 | | ND | | | **PWB** | 115.13 |
| | 2 | 4.12, d (9.1, 6.7) | 63.9 | | | | |
| | 3 | LB | 31.7 | | | | |
| | 4 | 1.99[a] | 26.5 | 5 | | | |
| | 5 | 3.41, 3.33[a] | 48.8 | 4 | | | |
| 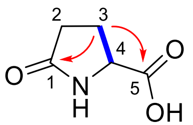 pyroglutamate (**Glp**) | 1 | | 184.5 | | | **PWB** | 129.11 |
| | 2 | 2.39[a] | 32.4 | | | | |
| | 3 | 2.50, m, Ha<br>2.02, m, Hb | 28.1 | 4 | 1, 5 | | |
| | 4 | 4.16, dd (9.0, 5.8) | 61.1 | 3 | | | |
| | 5 | | 183.1 | | | | |
| 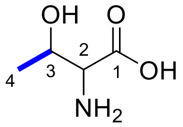 threonine (**Thr**) | 1 | | ND | | | **PWB** | 119.12 |
| | 2 | LB | ND | | | | |
| | 3 | 4.24[a] | ND | 4 | | | |
| | 4 | 1.32[a] | ND | 3 | | | |
| 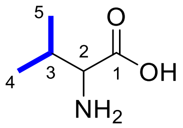 valine (**Val**) | 1 | | ND | | | **PWB** | 117.15 |
| | 2 | LB | ND | | | | |
| | 3 | 2.27[a] | ND | 4, 5 | | | |
| | 4 | 1.03, d (7.0) | 20.7 | 3 | | | |
| | 5 | 0.98, d (7.0) | 19.4 | 3 | | | |

[a]Multiplicity was not determined due to signal overlap.

**Supplementary Table 2.** BMRD codes of the identified metabolites

| metabolite name | BMRD code | metabolite name | BMRD code |
|---|---|---|---|
| **Sugar alcohols** | | **Organic acids** | |
| arabinitol (Ara) | bmse000068 | acetate (AcA) | bmse000857 |
| glycerol (Glo) | bmse000184 | azelate (AzA) | not available |
| mannitol (Man) | bmse000099 | formate (FA) | bmse000203 |
| **Sugars** | | fumarate (FmA) | bmse000083 |
| fructose (Frc) | bmse000010 | lactate (LA) | bmse000208 |
| glucose (Glc) | bmse000015 | malate (MA) | bmse000046 |
| sucrose (Suc) | bmse000119 | malonate (MnA) | bmse000386 |
| trehalose (Tre) | bmse000125 | succinate (SA) | bmse000183 |
| **Choline derivatives** | | **Amino acids** | |
| betaine (Bt) | bmse000069 | alanine (Ala) | bmse000028 |
| choline (Cho) | bmse000285 | $\gamma$-aminobutyrate (GABA) | bmse000340 |
| choline sulfate (CS) | - | asparagine (Asn) | bmse000030 |
| **Nucleosides** | | aspartate (Asp) | bmse000875 |
| adenosine (Ado) | bmse000061 | glutamate (Glu) | bmse000037 |
| uridine (Urd) | bmse000158 | glutamine (Gln) | bmse000038 |
| | | isoleucine (Ile) | bmse000041 |
| | | leucine (Leu) | bmse000042 |
| | | lysine (Lys) | bmse000043 |
| | | proline (Pro) | bmse000047 |
| | | pyroglutamate (Glp) | not available |
| | | threonine (Thr) | bmse000049 |
| | | valine (Val) | bmse000052 |

## Arabinitol (Ara)

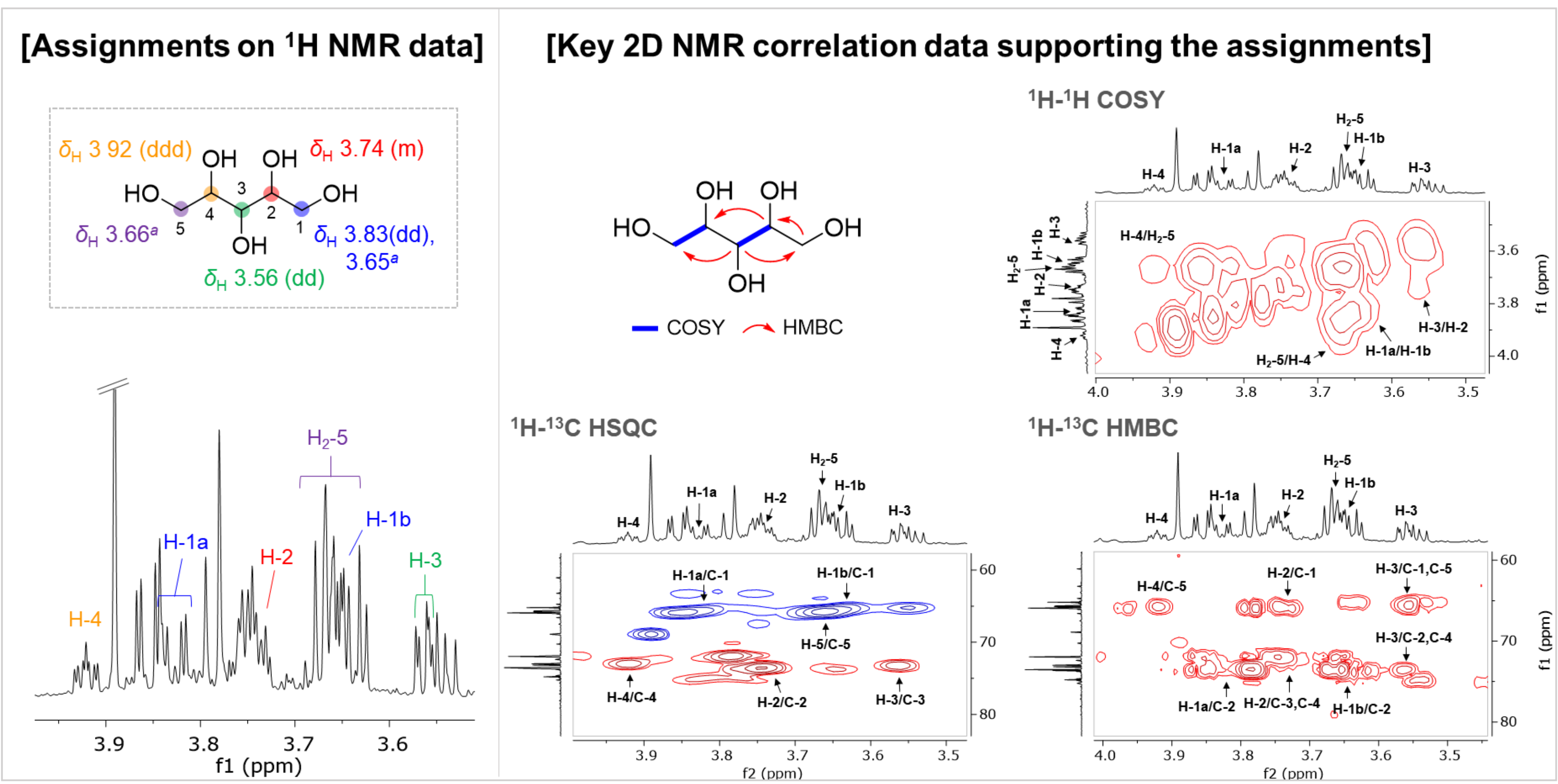


aMultiplicity was not determined due to signal overlapping.

Biological Magnetic Resonance Data Bank (BMRD) code: bmse000068

**Supplementary Figure 3.** Detailed 2D NMR (600 MHz, $D_2O$) data analysis of arabinitol (Ara) in **PWB**

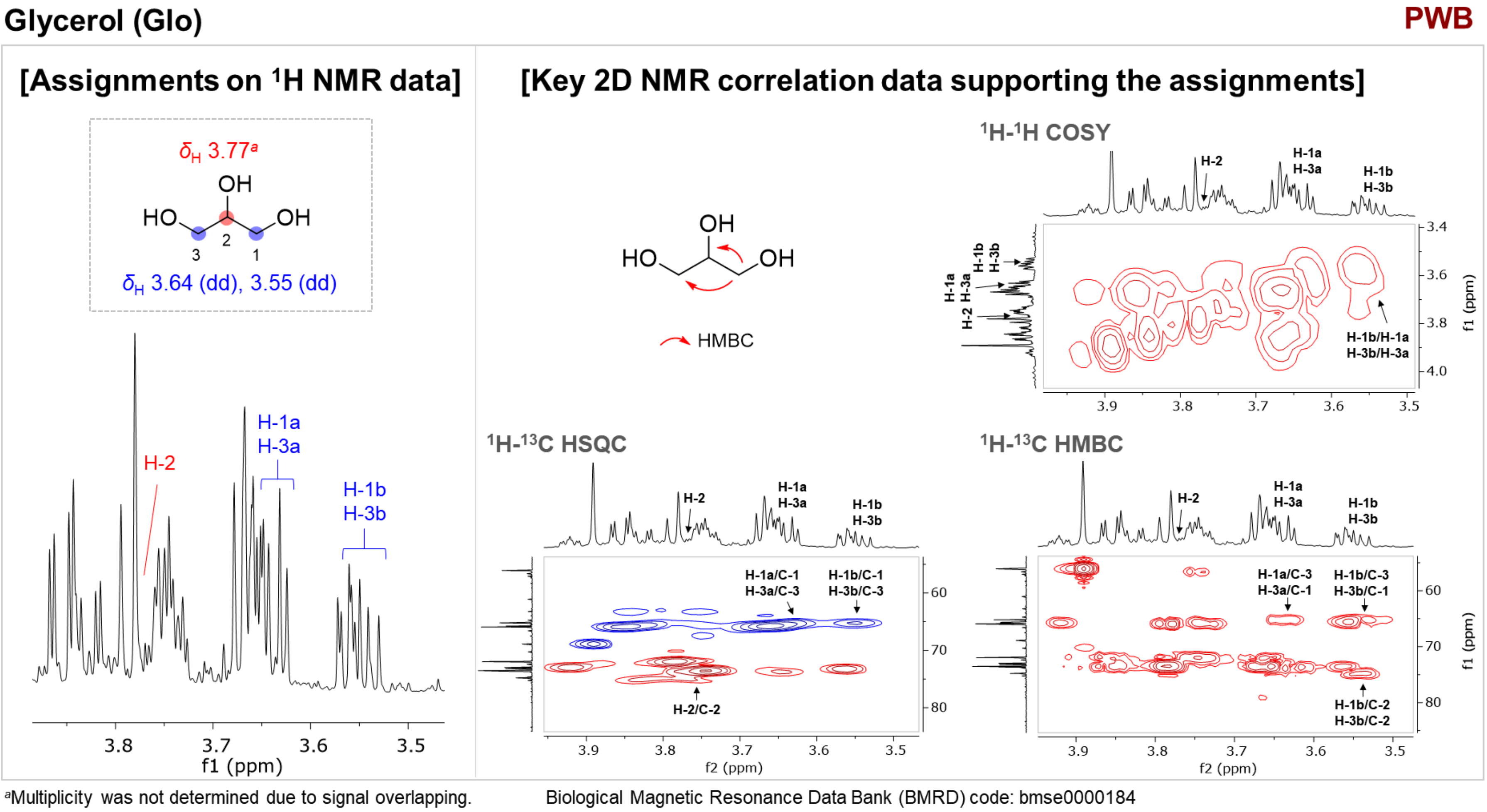


**Supplementary Figure 4.** Detailed 2D NMR (600 MHz, $D_2O$) data analysis of glycerol (Glo) in **PWB**

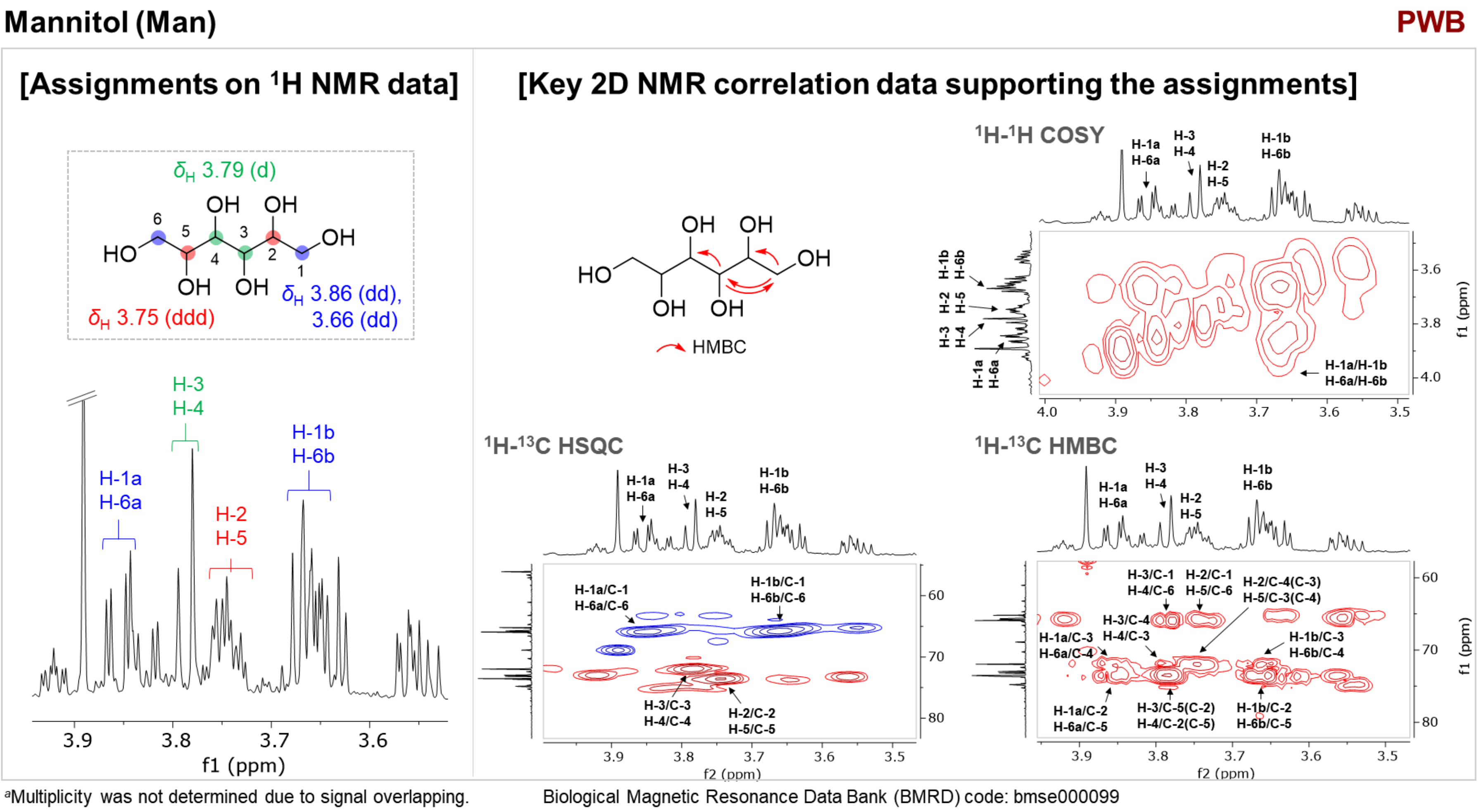


**Supplementary Figure 5.** Detailed 2D NMR (600 MHz, $D_2O$) data analysis of mannitol (Man) in **PWB**

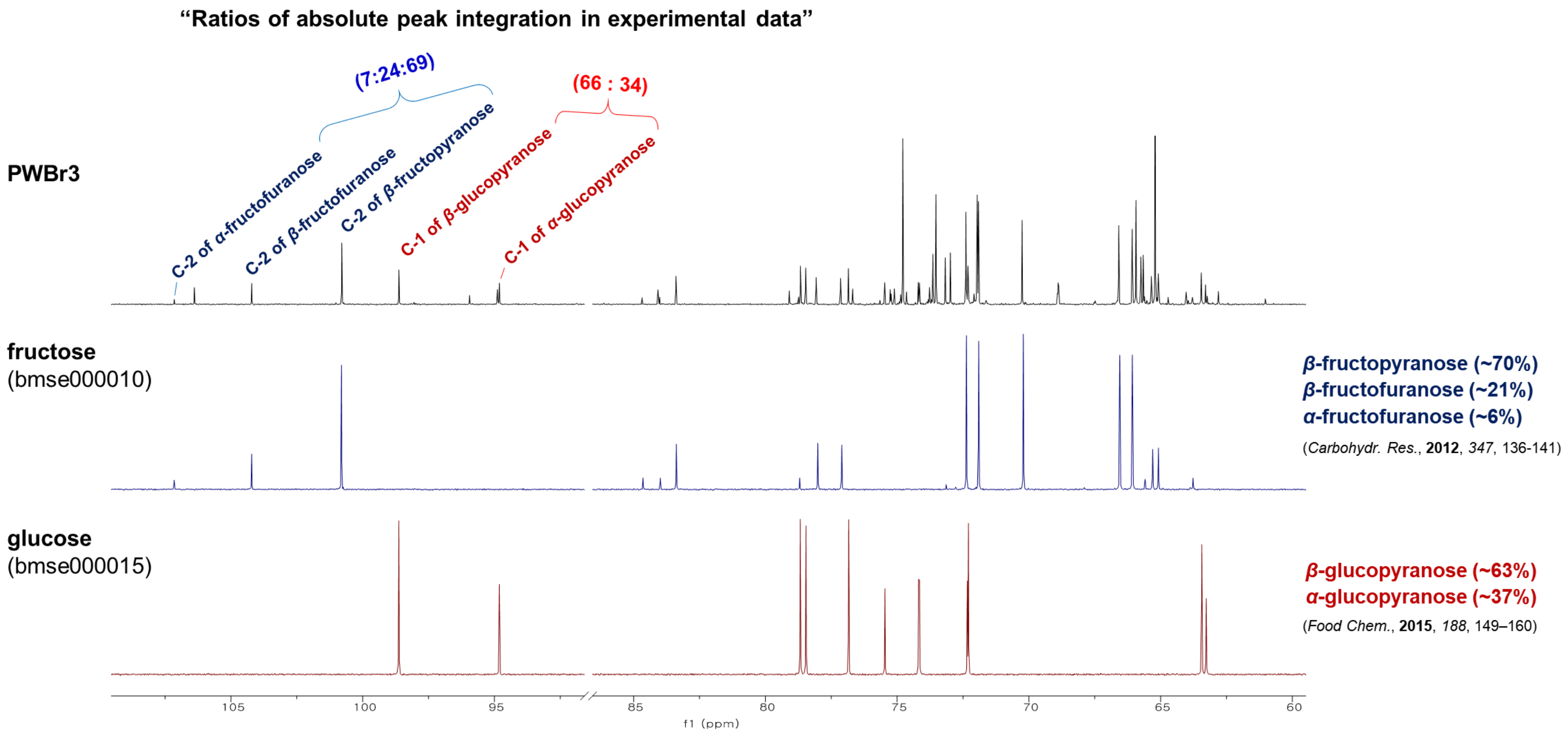


**Supplementary Figure 6.** $^{13}C$ NMR data (150 MHz, $D_2O$) of **PWBr3** with BMRD reference data of fructose (bmse000010) and glucose (bmse000015)

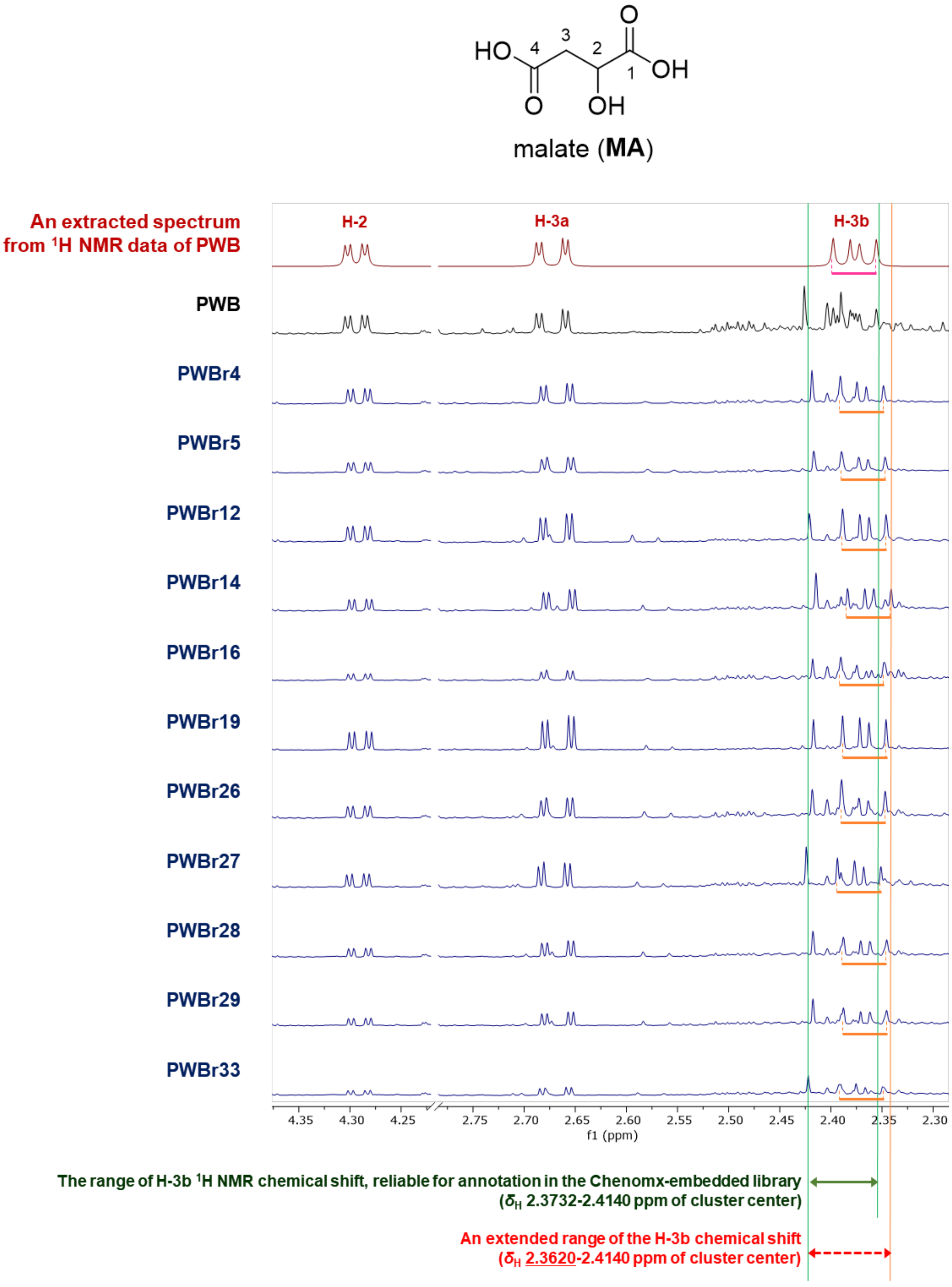


**Supplementary Figure 7.** Stacked $^1$H NMR (600 MHz, $D_2O$) spectra in an expansion for malate (MA) signals

## Malate (MA) PWB

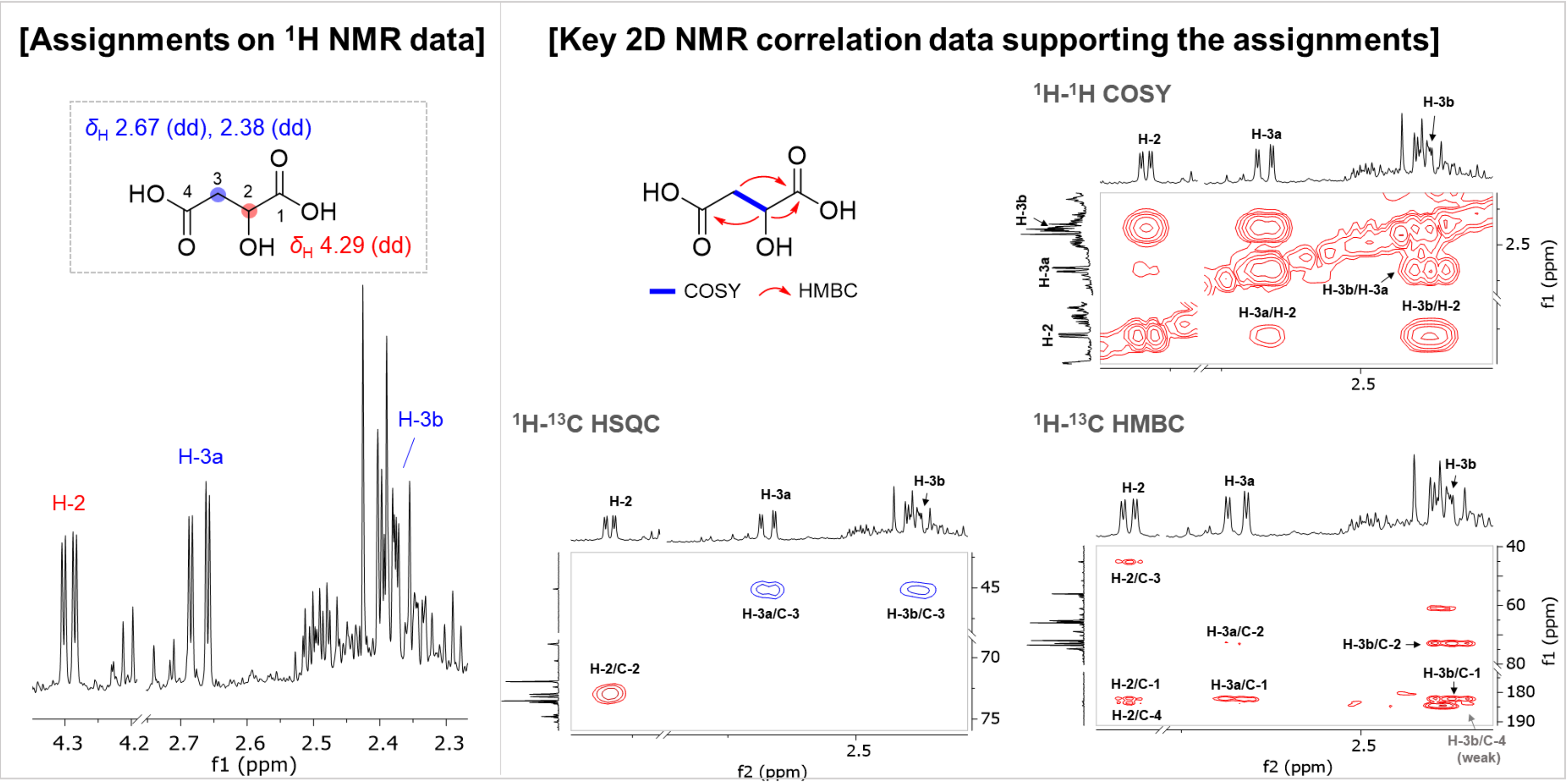


aMultiplicity was not determined due to signal overlapping. Biological Magnetic Resonance Data Bank (BMRD) code: bmse000046

**Supplementary Figure 8.** Detailed 2D NMR (600 MHz, $D_2O$) data analysis of malate (MA) in **PWB**

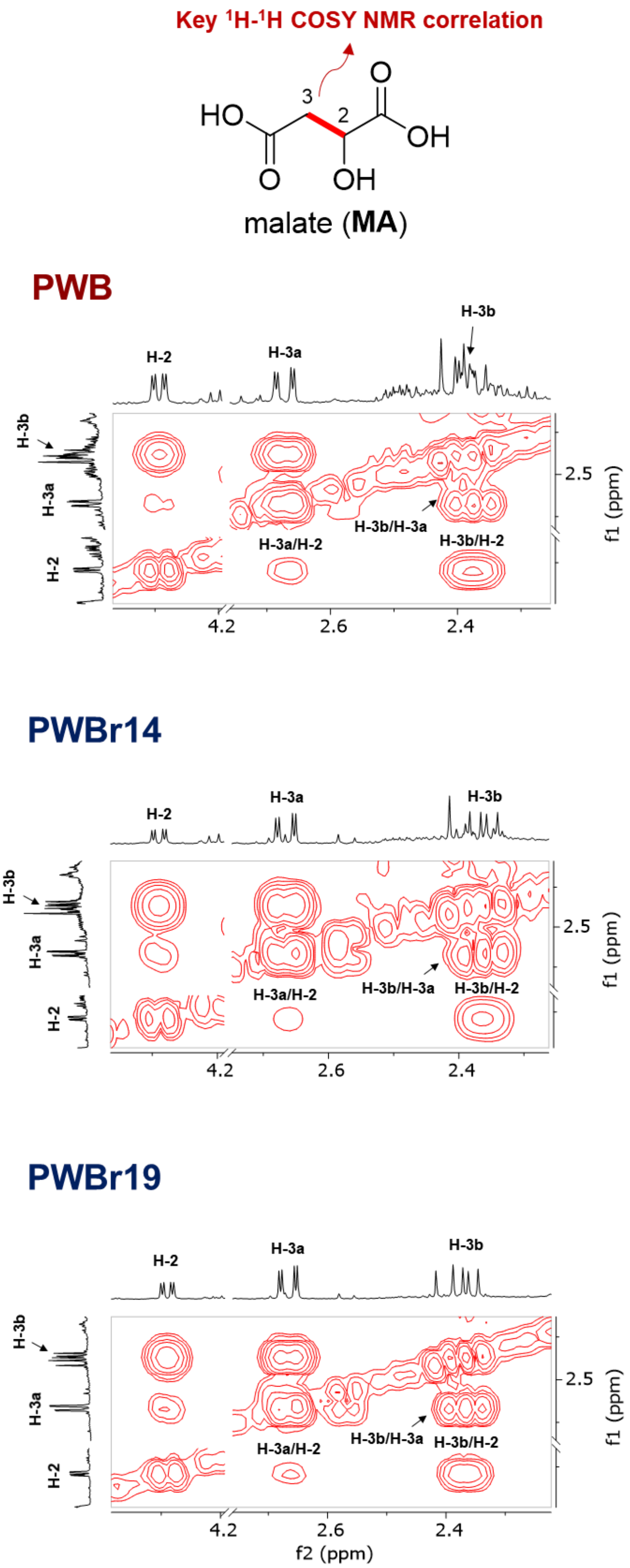


**Supplementary Figure 9.** Key $^1$H-$^1$H COSY (600 MHz, $D_2O$) correlations of malate in **PWB**, **PWBr14**, and **PWBr19**

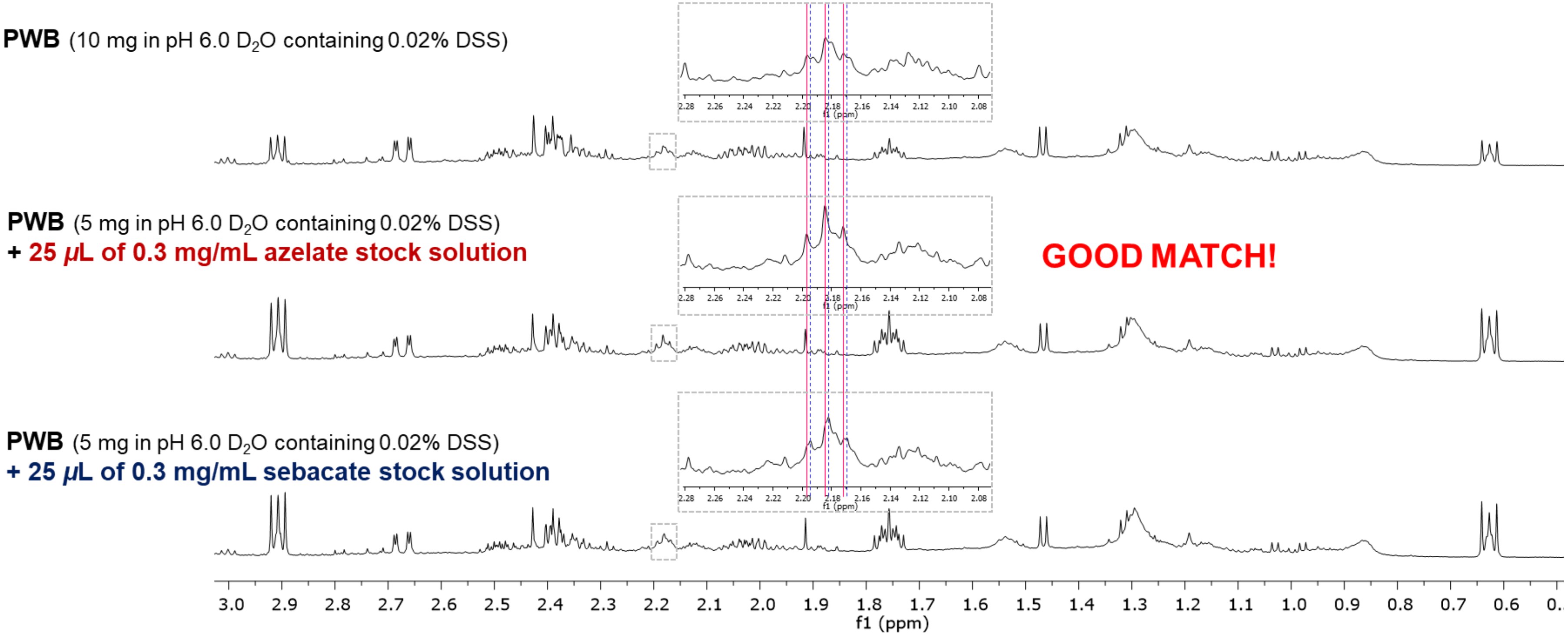


**Supplementary Figure 10.** Spiking $^{1}H$ NMR experiments (600 MHz, $D_2O$) with commercial standards of azelate and sebacate

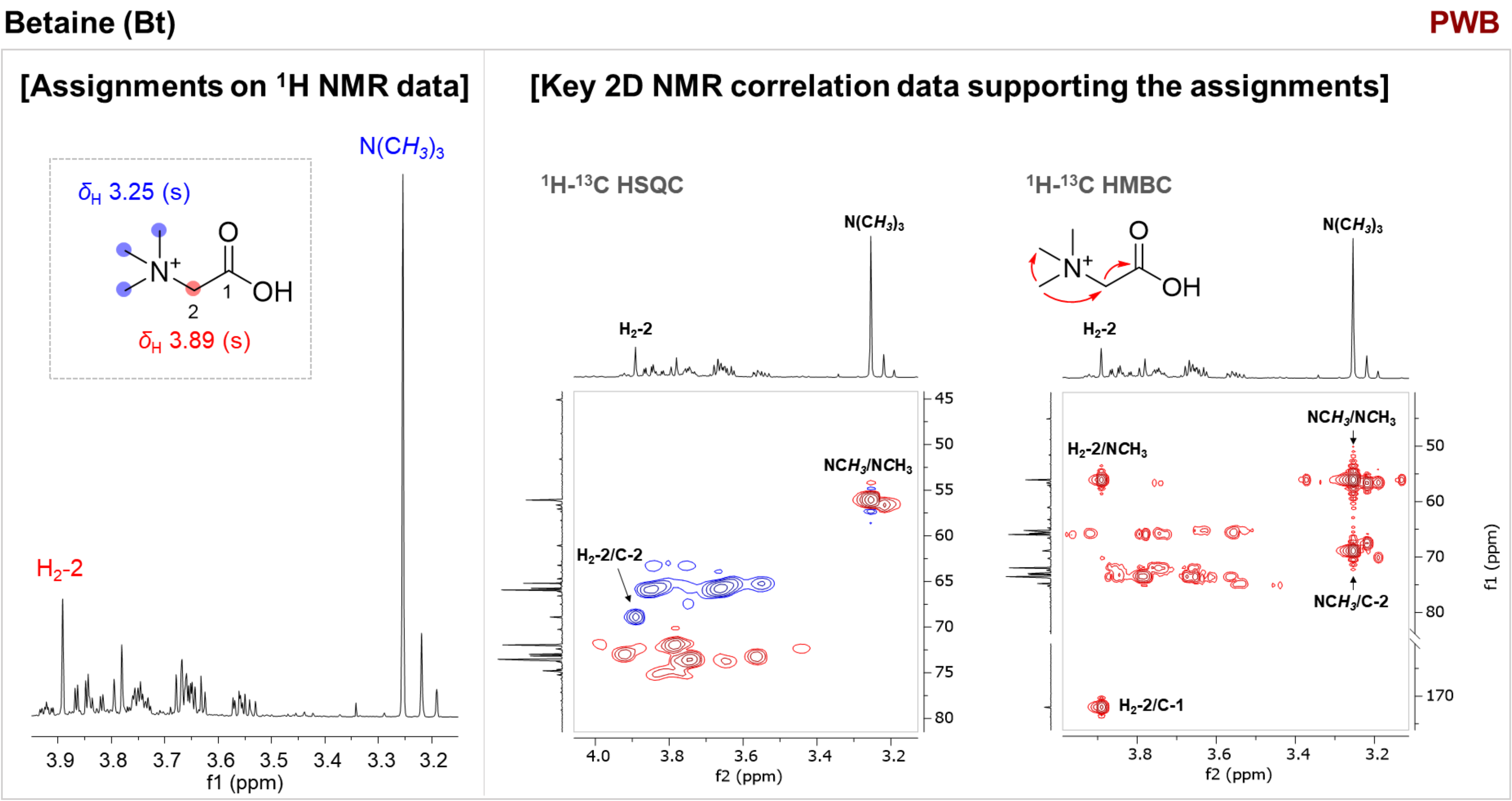


Biological Magnetic Resonance Data Bank (BMRD) code: bmse000069

**Supplementary Figure 11.** Detailed 2D NMR (600 MHz, $D_2O$) data analysis of betaine (Bt) in **PWB**

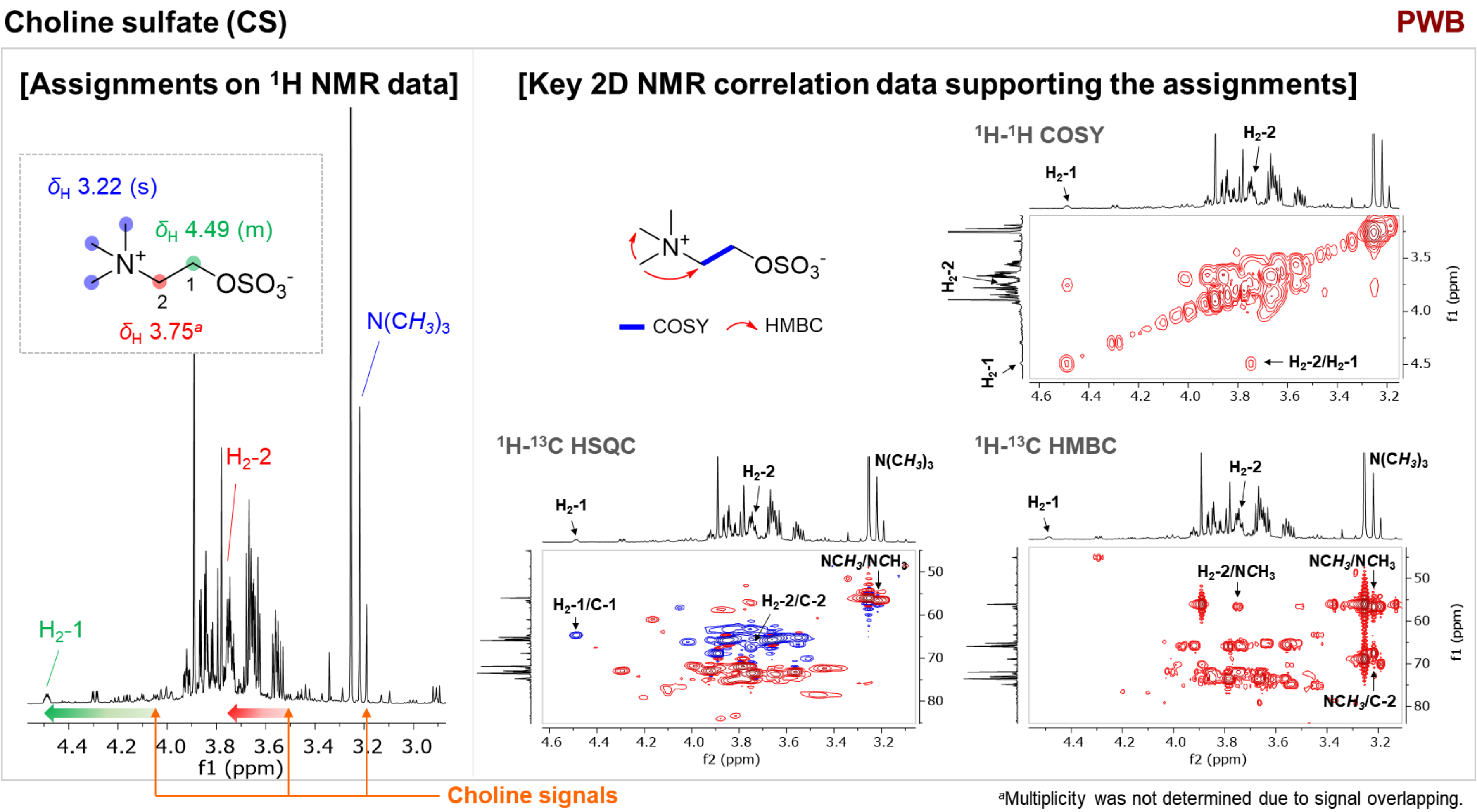


**Supplementary Figure 12.** Detailed 2D NMR (600 MHz, $D_2O$) data analysis of choline sulfate (CS) in **PWB**

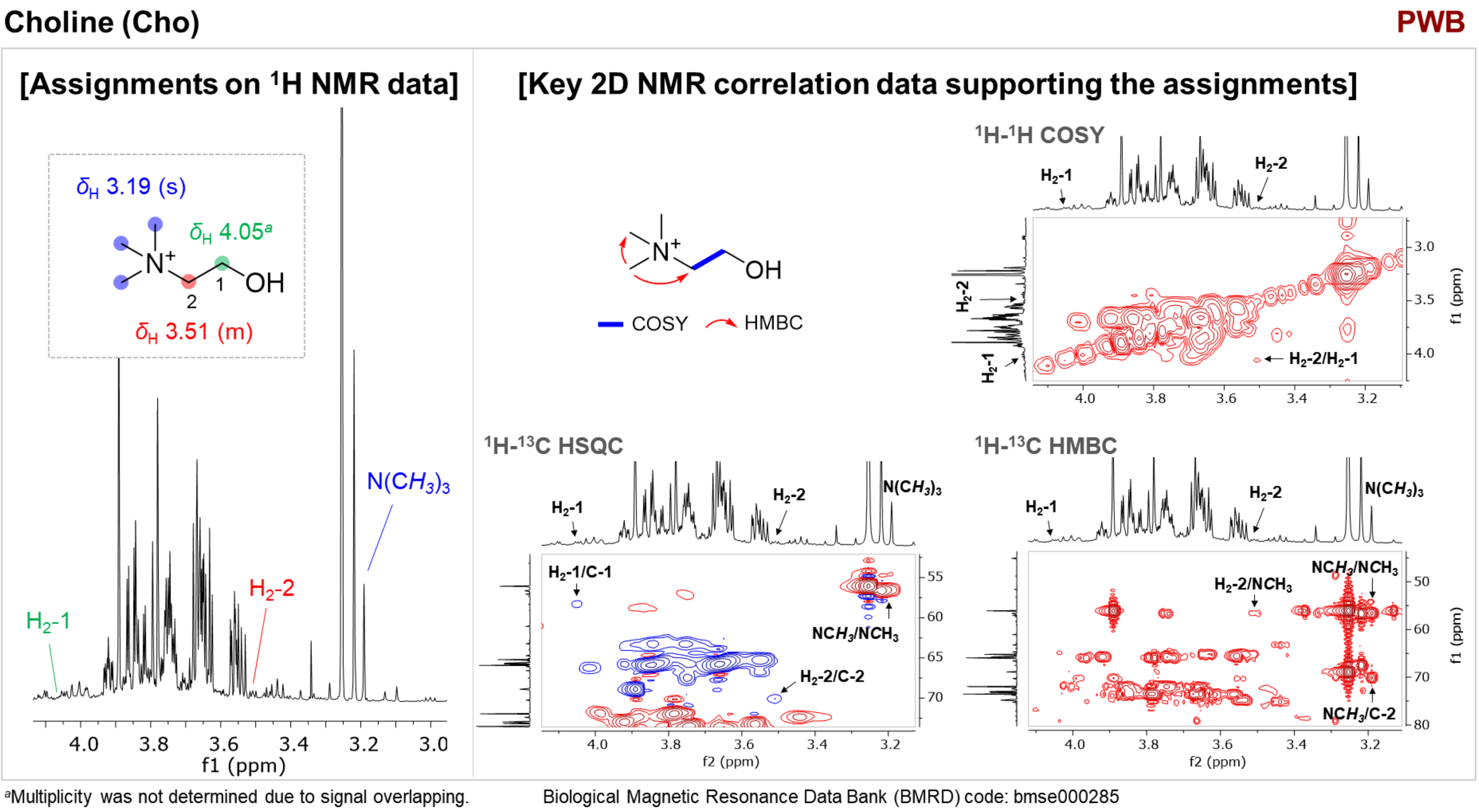


aMultiplicity was not determined due to signal overlapping.

Biological Magnetic Resonance Data Bank (BMRD) code: bmse000285

**Supplementary Figure 13.** Detailed 2D NMR (600 MHz, $D_2O$) data analysis of choline (Cho) in **PWB**

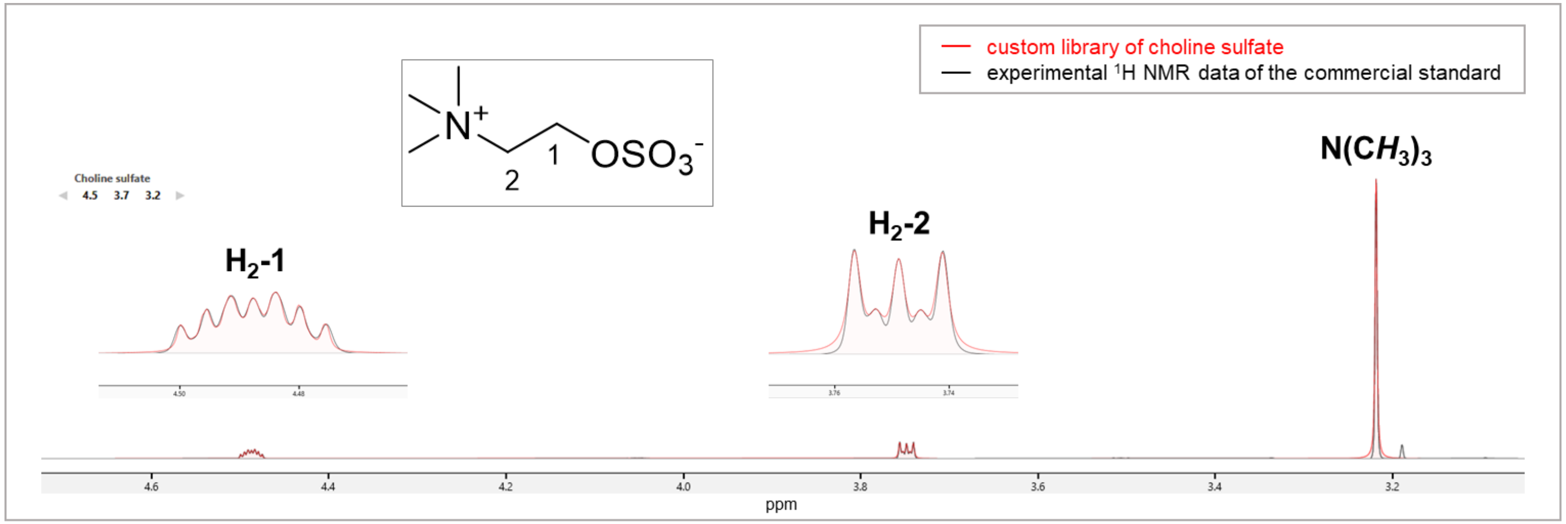


- **Sample information**: 2.00 mg/mL $D_2O$ solution (10.7 mM of choline sulfate of 98% standard sample purity)
- **$^1H$ NMR data acquisition parameter:** 1D NOESY presat pulse sequence (noesypr1d; presaturation frequency, $\delta_H$ 4.70 ppm) calibrated 90 degrees pulse (P1), D1 2 s, AQ 4 s, RG 64; NS 64; DS 2
- **$^1H$ NMR data processing parameter:** Fifth-order polynomial fitting, Lorentzian-to-Gaussian apodization (exponential factor -0.3, Gaussian factor 0.05)

<u>* The prepared custom Chenomx compound library is provided as xcpd file. Please cite this paper for use</u>.

**Supplementary Figure 14.** Preparation of the custom compound library for choline sulfate

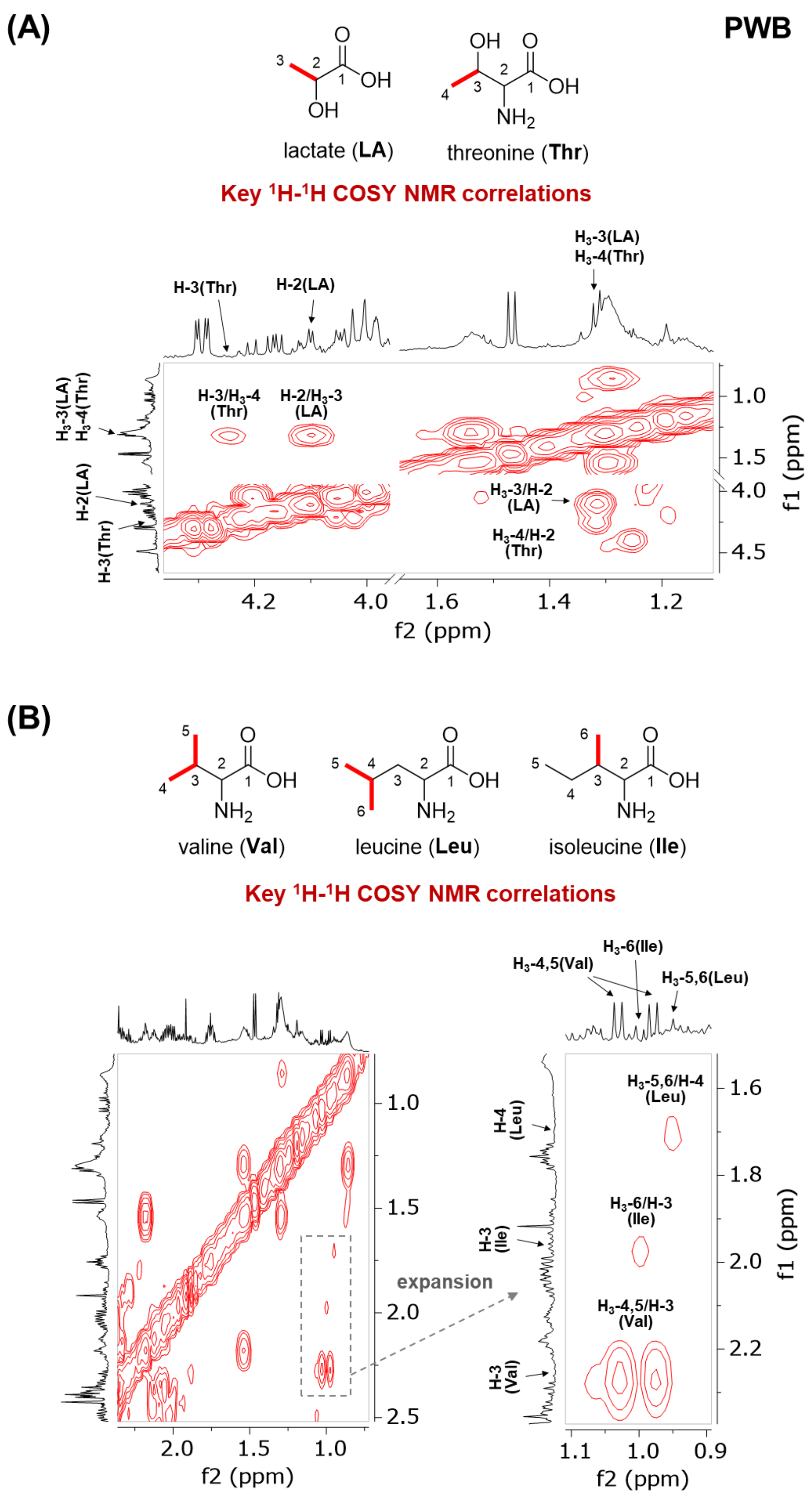


**Supplementary Figure 15.** Key $^1$H-$^1$H COSY (600 MHz, $D_2O$) correlations for identification of (A) lactate (LA) and threonine (Thr), and (B) valine (Val), leucine (Leu), and isoleucine (Ile) in **PWB**

**Supplementary Table 3.** Concentrations (mg/g dried extract) of the identified metabolites of the wheat bran samples

| Samples | GABA | AcA | Ado | Ala | Ara | Asn | Asp | AzA | Bt | Cho | CS | FA | Frc | FmA | Glc | Gln | Glu | Glo | Ile | LA | Leu | Lys | MA | MnA | Man | Pro | Glp | SA | Suc | Thr | Tre | Urd | Val |
|---|---|---|---|---|---|---|---|---|---|---|---|---|---|---|---|---|---|---|---|---|---|---|---|---|---|---|---|---|---|---|---|---|---|
| YWB1 | 1.671 | 0.080 | 0.451 | 2.078 | 35.578 | 0.318 | 0.665 | 2.300 | 39.136 | 2.945 | 5.718 | 0.100 | 27.330 | 0.032 | 11.086 | 1.454 | 3.250 | 59.023 | 0.440 | 0.845 | 0.436 | 1.266 | 7.483 | 0.962 | 60.054 | 2.650 | 7.349 | 0.234 | 5.673 | 0.378 | 9.276 | 0.835 | 0.646 |
| YWB2 | 0.314 | 3.655 | 0.247 | 0.983 | 37.223 | 0.373 | 0.541 | 1.114 | 19.346 | 2.080 | 5.611 | 0.748 | 8.280 | 0.147 | 0.968 | 1.906 | 1.363 | 26.917 | 0.302 | 0.943 | 0.349 | 0.221 | 5.712 | 0.429 | 57.148 | 0.930 | 2.010 | 0.603 | 2.331 | 0.465 | 13.787 | 0.527 | 0.382 |
| PWB | 1.475 | 0.926 | 0.478 | 2.566 | 46.353 | 1.113 | 1.468 | 2.424 | 58.195 | 2.754 | 4.783 | 0.989 | 14.403 | 0.458 | 1.397 | 4.635 | 4.021 | 17.450 | 0.596 | 0.940 | 0.665 | 0.559 | 14.130 | 1.240 | 62.304 | 3.003 | 9.682 | 1.475 | 5.760 | 1.130 | 16.046 | 0.925 | 1.175 |
| PWBr1 | 0.567 | 6.063 | 0.397 | 1.355 | 45.787 | 0.217 | 0.825 | 2.334 | 47.096 | 2.159 | 5.812 | 1.166 | 7.626 | 0.227 | 0.835 | 1.775 | 3.047 | 20.364 | 0.351 | 1.097 | 0.442 | 0.232 | 7.504 | 0.904 | 64.978 | 2.014 | 4.118 | 0.861 | 1.427 | 0.294 | 15.457 | 0.686 | 0.504 |
| PWBr2 | 0.248 | 0.540 | 0.209 | 1.162 | 30.856 | 0.169 | 0.420 | 3.481 | 41.697 | 1.964 | 3.697 | 0.745 | 14.173 | 0.196 | 4.353 | 1.975 | 2.235 | 21.002 | 0.405 | 1.212 | 0.405 | 0.099 | 13.044 | 0.893 | 36.659 | 2.232 | 3.993 | 1.047 | 8.252 | 1.027 | 6.428 | 0.439 | 0.455 |
| PWBr3 | 0.797 | 0.078 | 0.386 | 1.628 | 28.616 | 0.396 | 0.540 | 2.567 | 41.445 | 2.488 | 2.945 | 0.193 | 68.011 | 0.054 | 44.217 | 2.378 | 2.673 | 91.647 | 0.359 | 0.854 | 0.430 | 0.378 | 6.841 | 1.013 | 40.559 | 3.184 | 3.855 | 1.235 | 29.192 | 0.559 | 10.544 | 0.696 | 0.378 |
| PWBr4 | 0.109 | 2.545 | 0.188 | 0.805 | 34.677 | 0.106 | 0.503 | 2.302 | 38.319 | 1.608 | 4.466 | 1.378 | 8.307 | 0.473 | 1.937 | 1.492 | 1.596 | 23.963 | 0.232 | 1.122 | 0.333 | 0.023 | 13.013 | 0.713 | 41.605 | 1.166 | 3.100 | 1.123 | 2.043 | 0.479 | 9.998 | 0.426 | 0.294 |
| PWBr5 | 0.108 | 6.281 | 0.223 | 0.878 | 30.287 | 0.066 | 0.635 | 2.632 | 27.336 | 1.952 | 4.268 | 1.342 | 4.201 | 0.233 | 0.719 | 1.659 | 1.645 | 10.778 | 0.265 | 0.873 | 0.409 | 0.067 | 11.241 | 0.550 | 46.284 | 1.951 | 2.542 | 0.887 | 1.279 | 0.869 | 8.013 | 0.458 | 0.340 |
| PWBr6 | 1.964 | 0.088 | 0.484 | 2.999 | 41.377 | 1.477 | 1.522 | 1.609 | 47.635 | 2.458 | 3.420 | 0.292 | 7.963 | 0.187 | 0.202 | 4.895 | 3.452 | 33.791 | 0.441 | 0.872 | 0.507 | 1.112 | 6.352 | 1.014 | 90.649 | 3.540 | 11.410 | 0.820 | 2.618 | 1.161 | 21.479 | 0.784 | 0.888 |
| PWBr7 | 0.505 | 0.306 | 0.402 | 1.675 | 34.478 | 0.324 | 0.422 | 3.018 | 51.547 | 2.476 | 3.944 | 0.518 | 39.015 | 0.156 | 12.042 | 2.053 | 2.448 | 30.318 | 0.447 | 1.301 | 0.445 | 0.263 | 24.330 | 1.077 | 48.289 | 2.628 | 5.663 | 1.558 | 21.766 | 0.261 | 9.023 | 0.679 | 0.640 |
| PWBr8 | 0.486 | 0.159 | 0.197 | 1.031 | 45.209 | 0.203 | 0.459 | 1.463 | 40.145 | 2.244 | 4.593 | 0.128 | 23.021 | 0.030 | 24.821 | 1.548 | 2.380 | 128.754 | 0.333 | 0.905 | 0.337 | 0.238 | 4.414 | 0.901 | 71.347 | 2.236 | 3.755 | 1.132 | 3.388 | 0.350 | 14.074 | 0.746 | 0.333 |
| PWBr9 | 0.202 | 1.305 | 0.299 | 0.784 | 43.407 | 0.094 | 0.462 | 2.091 | 33.441 | 1.781 | 3.658 | 0.662 | 6.839 | 0.098 | 3.028 | 1.895 | 1.656 | 22.834 | 0.258 | 1.042 | 0.292 | 0.104 | 5.194 | 0.688 | 58.021 | 1.431 | 2.743 | 0.906 | 1.263 | 0.501 | 16.221 | 0.529 | 0.247 |
| PWBr10 | 0.835 | 0.080 | 0.282 | 1.453 | 42.343 | 0.300 | 0.635 | 1.712 | 40.605 | 2.368 | 4.815 | 0.123 | 10.795 | 0.043 | 5.736 | 2.121 | 2.076 | 25.024 | 0.338 | 0.717 | 0.364 | 0.544 | 3.344 | 0.852 | 71.857 | 2.188 | 3.520 | 0.802 | 7.569 | 0.513 | 10.596 | 0.587 | 0.437 |
| PWBr11 | 0.504 | 0.213 | 0.208 | 1.045 | 36.451 | 0.222 | 0.796 | 1.714 | 33.087 | 1.803 | 3.883 | 0.275 | 24.752 | 0.011 | 25.314 | 1.659 | 2.410 | 71.504 | 0.307 | 0.894 | 0.341 | 0.279 | 6.739 | 0.813 | 48.454 | 2.274 | 3.644 | 0.960 | 8.612 | 0.407 | 10.791 | 0.551 | 0.331 |
| PWBr12 | 0.200 | 0.404 | 0.091 | 0.704 | 24.332 | 0.073 | 0.348 | 1.417 | 22.952 | 1.045 | 2.457 | 0.597 | 20.456 | 0.140 | 10.182 | 1.877 | 2.173 | 23.363 | 0.214 | 0.811 | 0.208 | 0.083 | 18.342 | 0.502 | 31.793 | 1.436 | 2.002 | 1.024 | 6.979 | 0.204 | 8.530 | 0.366 | 0.228 |
| PWBr13 | 0.586 | 1.699 | 0.368 | 1.725 | 45.402 | 0.503 | 1.039 | 2.178 | 33.997 | 2.281 | 4.924 | 0.969 | 6.208 | 0.246 | 0.217 | 3.669 | 2.339 | 21.210 | 0.339 | 0.752 | 0.422 | 0.371 | 7.059 | 0.723 | 76.180 | 1.517 | 4.977 | 0.747 | 1.597 | 1.077 | 15.252 | 0.632 | 0.573 |
| PWBr14 | 0.153 | 8.060 | 0.143 | 0.825 | 37.127 | 0.149 | 0.624 | 2.177 | 27.712 | 1.787 | 4.196 | 0.966 | 5.666 | 0.142 | 1.589 | 2.893 | 2.332 | 13.413 | 0.244 | 1.007 | 0.367 | 0.071 | 12.507 | 0.574 | 44.009 | 1.977 | 2.916 | 1.199 | 4.229 | 0.356 | 11.181 | 0.470 | 0.321 |
| PWBr15 | 1.046 | 0.478 | 0.408 | 2.320 | 37.618 | 0.683 | 0.909 | 1.847 | 39.272 | 2.760 | 3.737 | 0.382 | 32.744 | 0.118 | 9.200 | 1.939 | 3.250 | 42.118 | 0.450 | 1.035 | 0.480 | 0.484 | 12.391 | 0.937 | 50.206 | 5.336 | 7.007 | 0.923 | 24.325 | 0.278 | 14.542 | 0.828 | 0.720 |
| PWBr16 | 0.267 | 19.150 | 0.365 | 1.405 | 33.640 | 0.331 | 0.762 | 1.222 | 29.146 | 2.127 | 4.438 | 0.943 | 3.585 | 0.238 | 0.061 | 4.847 | 2.462 | 14.055 | 0.290 | 0.991 | 0.393 | 0.037 | 6.703 | 0.551 | 61.059 | 1.080 | 4.497 | 0.731 | 1.837 | 0.620 | 18.013 | 0.612 | 0.536 |
| PWBr17 | 0.341 | 0.330 | 0.142 | 1.182 | 26.128 | 0.196 | 0.543 | 2.878 | 42.570 | 1.753 | 2.577 | 0.432 | 15.780 | 0.150 | 3.963 | 1.486 | 1.724 | 23.529 | 0.231 | 0.886 | 0.368 | 0.144 | 13.272 | 0.845 | 27.922 | 2.714 | 4.556 | 1.058 | 7.030 | 0.584 | 4.834 | 0.394 | 0.453 |
| PWBr18 | 0.291 | 1.235 | 0.065 | 0.769 | 25.166 | 0.163 | 0.509 | 1.193 | 23.174 | 1.488 | 2.575 | 0.351 | 46.332 | 0.035 | 49.102 | 1.190 | 1.696 | 82.814 | 0.213 | 0.554 | 0.308 | 0.113 | 6.378 | 0.540 | 45.517 | 2.831 | 2.821 | 0.735 | 18.406 | 0.277 | 10.704 | 0.464 | 0.261 |
| PWBr19 | 0.026 | 17.552 | 0.162 | 0.384 | 29.978 | 0.103 | 0.136 | 1.133 | 24.697 | 1.289 | 3.717 | 1.166 | 1.888 | 0.313 | 0.452 | 1.212 | 0.919 | 6.591 | 0.138 | 0.437 | 0.200 | 0.009 | 19.768 | 0.447 | 33.677 | 0.791 | 1.179 | 0.981 | 1.649 | 0.254 | 5.568 | 0.359 | 0.161 |
| PWBr20 | 0.493 | 0.198 | 0.262 | 1.138 | 46.716 | 0.271 | 0.641 | 1.699 | 31.009 | 2.062 | 2.931 | 0.374 | 58.016 | 0.117 | 20.476 | 1.639 | 2.129 | 81.132 | 0.274 | 1.175 | 0.463 | 0.256 | 7.094 | 0.735 | 58.113 | 2.886 | 2.736 | 0.868 | 8.553 | 0.279 | 15.515 | 0.596 | 0.407 |
| PWBr21 | 0.176 | 0.125 | 0.155 | 0.879 | 50.303 | 0.160 | 0.455 | 0.991 | 15.786 | 1.887 | 3.642 | 0.282 | 22.187 | 0.076 | 8.758 | 1.240 | 1.819 | 84.216 | 0.324 | 1.041 | 0.325 | 0.159 | 3.006 | 0.369 | 62.813 | 1.323 | 1.732 | 0.636 | 16.257 | 0.236 | 12.792 | 0.456 | 0.303 |
| PWBr22 | 0.499 | 0.140 | 0.212 | 1.385 | 33.562 | 0.081 | 0.394 | 2.579 | 29.406 | 2.681 | 1.993 | 0.302 | 89.769 | 0.058 | 50.026 | 1.773 | 2.258 | 57.311 | 0.349 | 1.095 | 0.391 | 0.373 | 7.221 | 0.670 | 25.560 | 3.867 | 2.844 | 0.923 | 35.113 | 0.149 | 5.381 | 0.523 | 0.510 |
| PWBr23 | 0.146 | 0.162 | 0.050 | 0.645 | 38.926 | 0.124 | 0.407 | 1.995 | 19.205 | 1.574 | 2.488 | 0.829 | 76.267 | 0.225 | 40.217 | 1.713 | 1.773 | 92.037 | 0.196 | 1.085 | 0.293 | 0.092 | 8.368 | 0.448 | 42.515 | 2.675 | 1.978 | 1.074 | 7.454 | 0.162 | 8.939 | 0.471 | 0.232 |
| PWBr24 | 0.707 | 0.139 | 0.269 | 1.468 | 50.894 | 0.288 | 0.552 | 1.670 | 30.185 | 2.185 | 3.201 | 0.138 | 45.344 | 0.071 | 12.467 | 1.853 | 2.226 | 83.989 | 0.383 | 0.885 | 0.367 | 0.665 | 6.898 | 0.698 | 65.487 | 2.224 | 3.970 | 0.928 | 4.720 | 0.190 | 10.688 | 0.600 | 0.458 |
| PWBr25 | 0.840 | 0.230 | 0.363 | 1.932 | 29.805 | 0.625 | 0.612 | 1.982 | 39.510 | 2.863 | 2.093 | 0.248 | 78.831 | 0.104 | 34.820 | 1.313 | 3.142 | 53.963 | 0.296 | 0.962 | 0.383 | 0.388 | 18.161 | 0.944 | 39.709 | 5.407 | 5.414 | 1.477 | 29.499 | 0.234 | 7.899 | 0.667 | 0.550 |
| PWBr26 | 0.473 | 3.789 | 0.298 | 1.608 | 47.439 | 0.572 | 0.993 | 2.562 | 42.138 | 2.358 | 5.861 | 1.311 | 7.832 | 0.385 | 0.549 | 3.385 | 2.494 | 21.367 | 0.348 | 1.103 | 0.432 | 0.320 | 13.484 | 0.880 | 70.601 | 1.625 | 5.607 | 1.034 | 3.733 | 1.016 | 13.601 | 0.558 | 0.590 |
| PWBr27 | 0.335 | 0.830 | 0.266 | 1.205 | 33.798 | 0.266 | 0.754 | 2.522 | 48.649 | 2.037 | 3.584 | 0.897 | 20.636 | 0.167 | 6.841 | 1.493 | 2.068 | 37.308 | 0.286 | 1.228 | 0.345 | 0.152 | 14.878 | 0.975 | 42.525 | 2.310 | 2.938 | 1.245 | 7.921 | 0.154 | 7.454 | 0.545 | 0.392 |
| PWBr28 | 0.181 | 6.346 | 0.330 | 0.986 | 40.253 | 0.152 | 0.519 | 1.768 | 30.044 | 2.015 | 5.338 | 1.185 | 7.920 | 0.213 | 1.055 | 2.390 | 2.239 | 22.634 | 0.250 | 1.071 | 0.316 | 0.089 | 10.540 | 0.599 | 64.175 | 1.319 | 1.983 | 0.896 | 2.222 | 0.368 | 12.741 | 0.489 | 0.321 |
| PWBr29 | 0.140 | 7.594 | 0.253 | 0.891 | 43.944 | 0.144 | 0.587 | 1.837 | 26.339 | 1.822 | 4.706 | 1.382 | 13.238 | 0.203 | 2.423 | 1.855 | 2.236 | 35.011 | 0.308 | 1.211 | 0.364 | 0.097 | 8.692 | 0.540 | 65.402 | 1.370 | 2.098 | 0.880 | 2.716 | 0.178 | 13.041 | 0.383 | 0.327 |
| PWBr30 | 0.360 | 0.111 | 0.228 | 1.100 | 41.688 | 0.184 | 0.298 | 1.187 | 24.308 | 1.639 | 2.874 | 0.265 | 91.741 | 0.164 | 62.779 | 1.895 | 1.658 | 183.155 | 0.196 | 0.869 | 0.250 | 0.219 | 6.784 | 0.576 | 56.751 | 3.100 | 2.528 | 1.022 | 13.535 | 0.160 | 12.539 | 0.553 | 0.203 |
| PWBr31 | 0.518 | 0.181 | 0.320 | 1.587 | 41.749 | 0.348 | 0.608 | 1.913 | 41.593 | 2.218 | 3.388 | 0.357 | 27.357 | 0.140 | 7.608 | 1.872 | 3.086 | 48.318 | 0.362 | 1.244 | 0.434 | 0.321 | 11.583 | 0.882 | 60.228 | 2.389 | 4.752 | 1.342 | 13.326 | 0.341 | 10.628 | 0.565 | 0.592 |
| PWBr32 | 0.233 | 1.425 | 0.288 | 0.905 | 40.007 | 0.085 | 0.500 | 1.367 | 28.094 | 2.216 | 5.957 | 1.049 | 14.050 | 0.105 | 4.179 | 1.687 | 1.897 | 27.894 | 0.312 | 1.128 | 0.293 | 0.145 | 4.503 | 0.578 | 80.762 | 1.138 | 2.725 | 0.808 | 6.158 | 0.147 | 10.456 | 0.544 | 0.323 |
| PWBr33 | 0.140 | 0.332 | 0.249 | 0.778 | 41.197 | 0.084 | 0.301 | 2.033 | 25.493 | 1.636 | 3.277 | 0.679 | 12.773 | 0.107 | 3.543 | 1.310 | 1.547 | 33.621 | 0.214 | 1.214 | 0.222 | 0.049 | 5.435 | 0.499 | 48.199 | 0.985 | 1.984 | 0.701 | 2.786 | 0.329 | 8.624 | 0.498 | 0.249 |
| PWBr34 | 0.353 | 0.110 | 0.282 | 1.197 | 50.270 | 0.190 | 0.364 | 1.828 | 28.506 | 1.994 | 3.298 | 0.287 | 47.938 | 0.085 | 19.306 | 1.340 | 2.053 | 101.679 | 0.265 | 1.093 | 0.303 | 0.189 | 4.147 | 0.616 | 69.984 | 2.361 | 2.659 | 0.600 | 5.510 | 0.175 | 15.021 | 0.550 | 0.324 |
| PWBr35 | 0.231 | 0.103 | 0.181 | 0.958 | 50.368 | 0.070 | 0.273 | 2.436 | 18.610 | 1.719 | 3.251 | 0.138 | 41.922 | 0.018 | 27.738 | 1.501 | 1.654 | 94.144 | 0.249 | 1.000 | 0.276 | 0.281 | 3.155 | 0.399 | 65.426 | 2.008 | 2.604 | 0.515 | 13.908 | 0.235 | 10.837 | 0.384 | 0.226 |
| PWBr36 | 0.216 | 0.087 | 0.196 | 0.698 | 42.931 | 0.161 | 0.336 | 1.256 | 17.858 | 1.330 | 2.016 | 0.297 | 97.292 | 0.128 | 60.665 | 1.472 | 1.087 | 145.706 | 0.204 | 0.912 | 0.233 | 0.155 | 4.518 | 0.431 | 52.231 | 1.662 | 1.429 | 0.760 | 9.738 | 0.052 | 11.768 | 0.439 | 0.181 |
| PWBr37 | 0.210 | 0.090 | 0.155 | 0.917 | 39.922 | 0.113 | 0.205 | 2.335 | 27.633 | 1.594 | 2.571 | 0.222 | 87.765 | 0.086 | 51.495 | 1.487 | 1.479 | 80.592 | 0.221 | 1.007 | 0.247 | 0.204 | 8.806 | 0.577 | 44.112 | 1.644 | 1.903 | 1.191 | 23.583 | 0.210 | 7.605 | 0.474 | 0.218 |

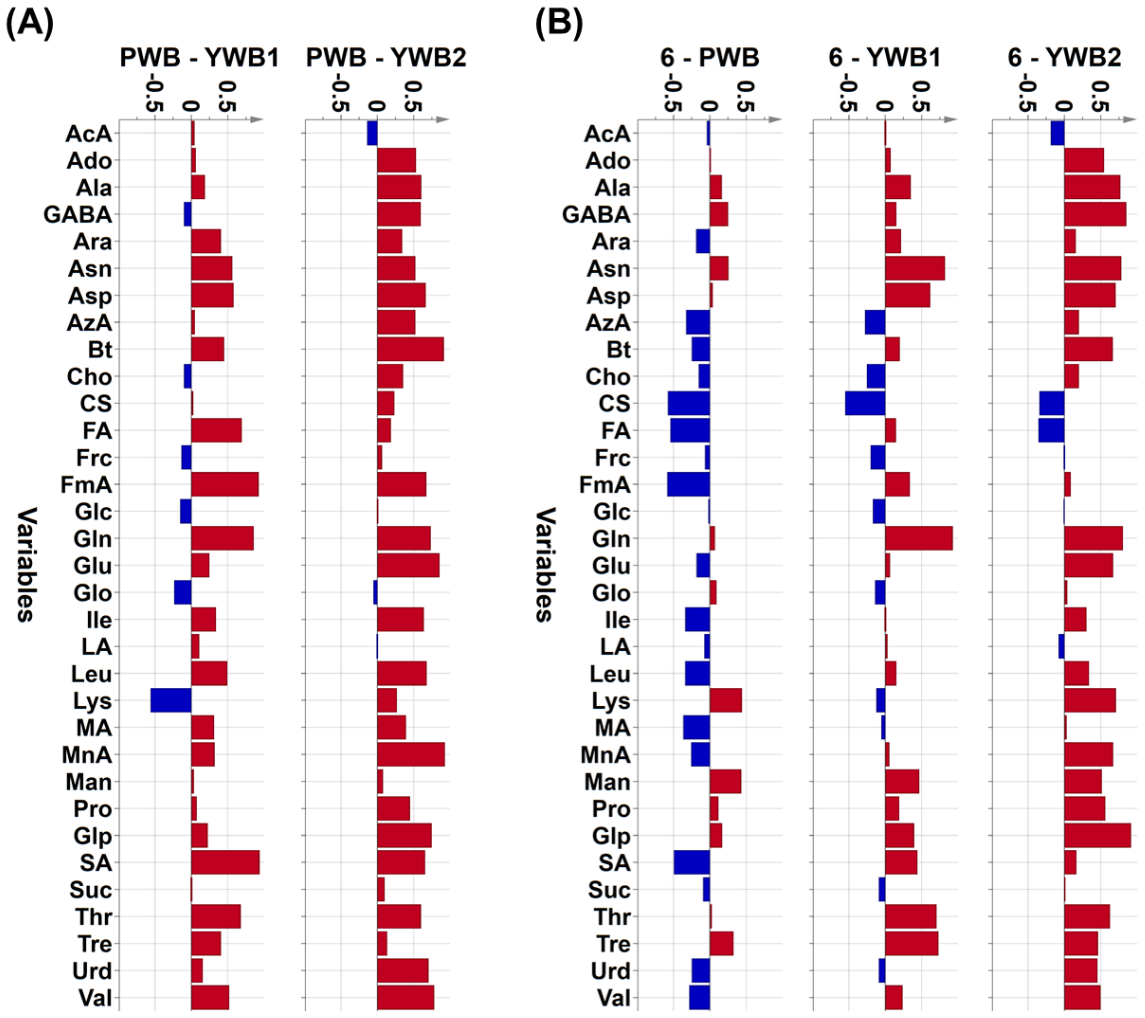


**Supplementary Figure 16.** Subtraction plots of (A) **PWB** with **YWB1** (left) or **YWB2** (right), and (B) **PWBr6** with **PWB**, **YWB1**, or **YWB2** (left to right) for 33 variables (quantification data were rescaled by min-max normalization to [0, 1] for plotting; positive or negative values were colored red or blue, respectively)

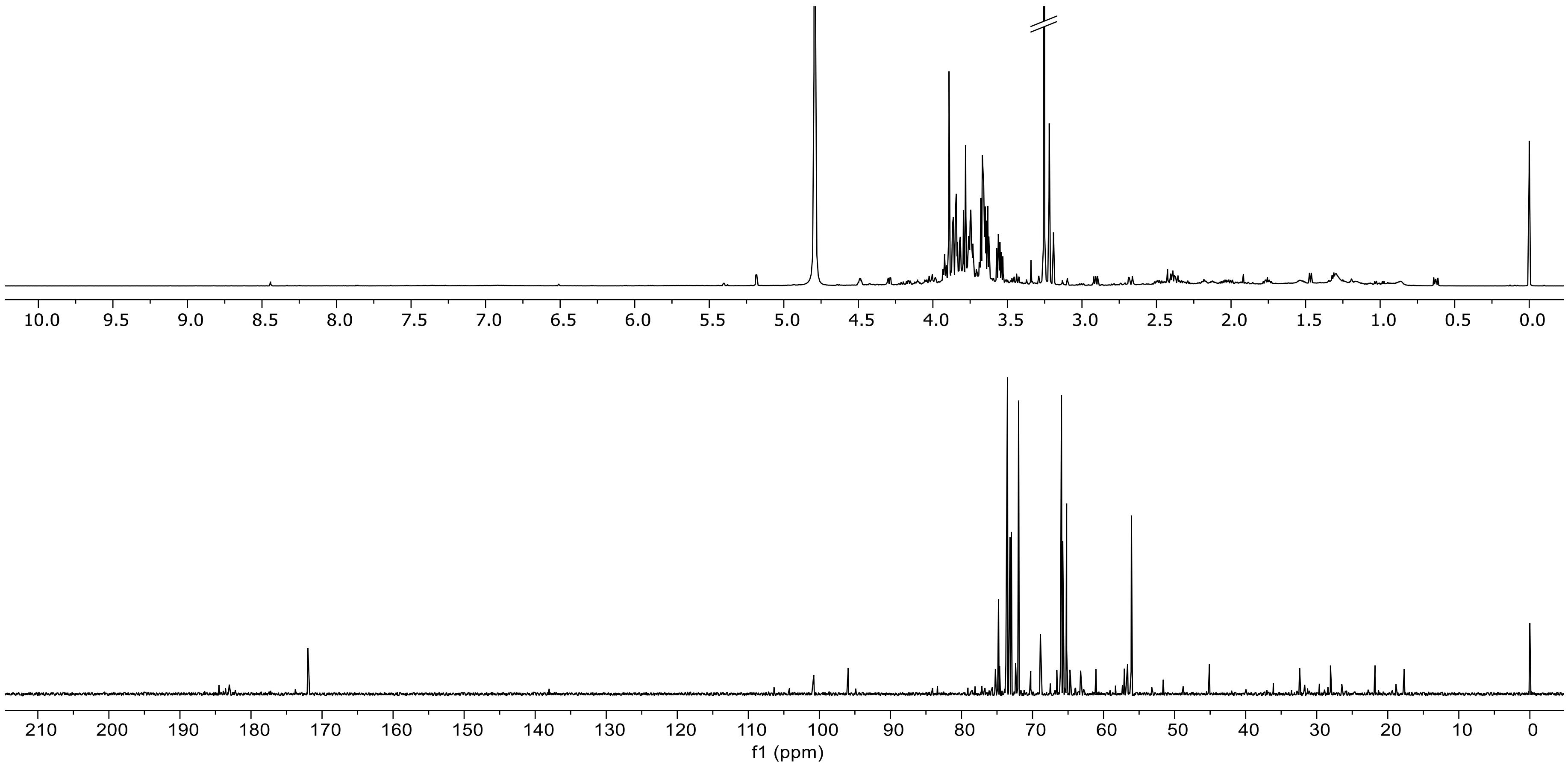


**Supplementary Figure 17.** $^{1}H$ (top, 600 MHz) and $^{13}C$ (bottom, 150 MHz) NMR spectra ($D_2O$) of **PWB**

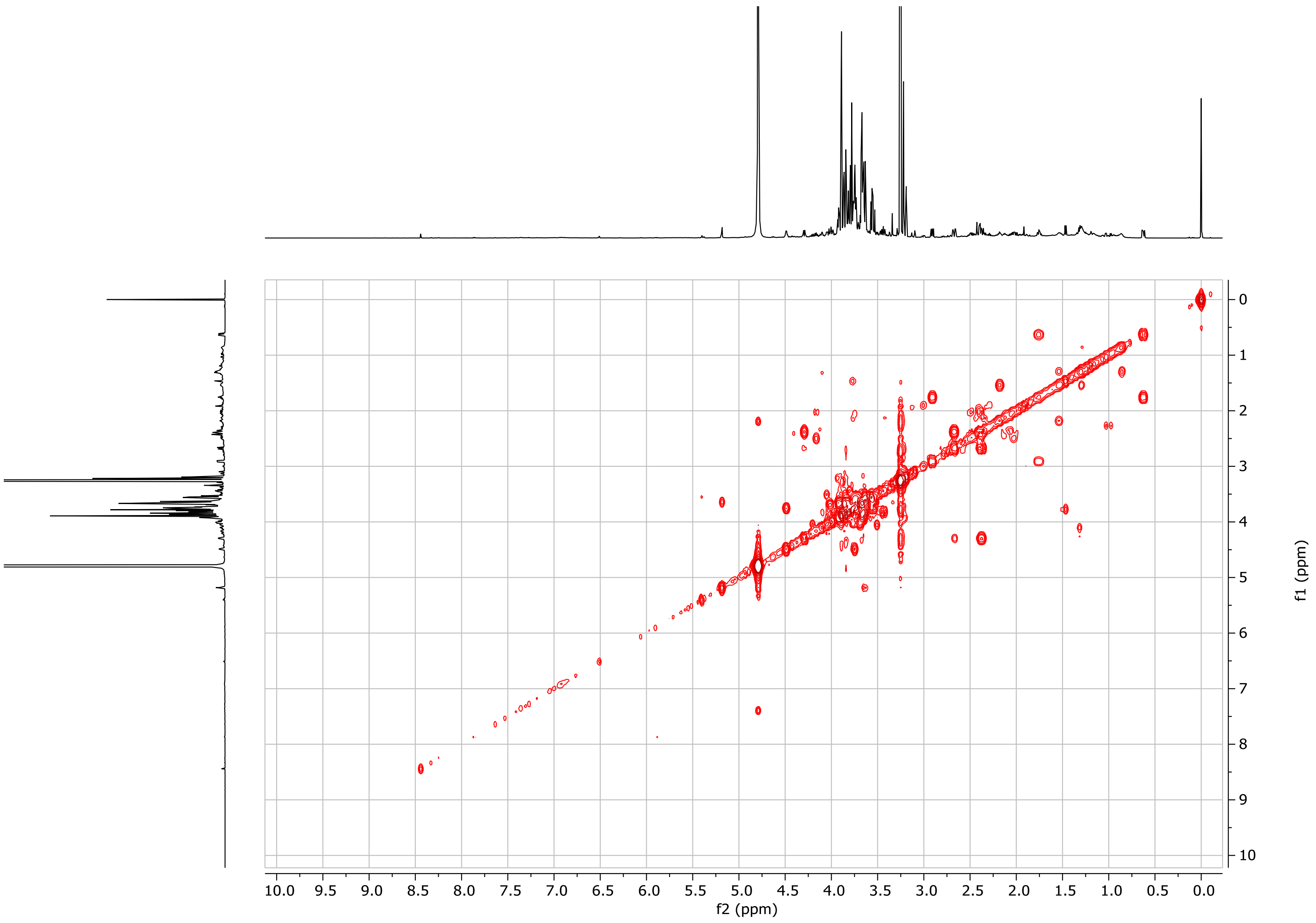


**Supplementary Figure 18.** $^{1}H$-$^{1}H$ COSY spectrum ($D_2O$) of **PWB**

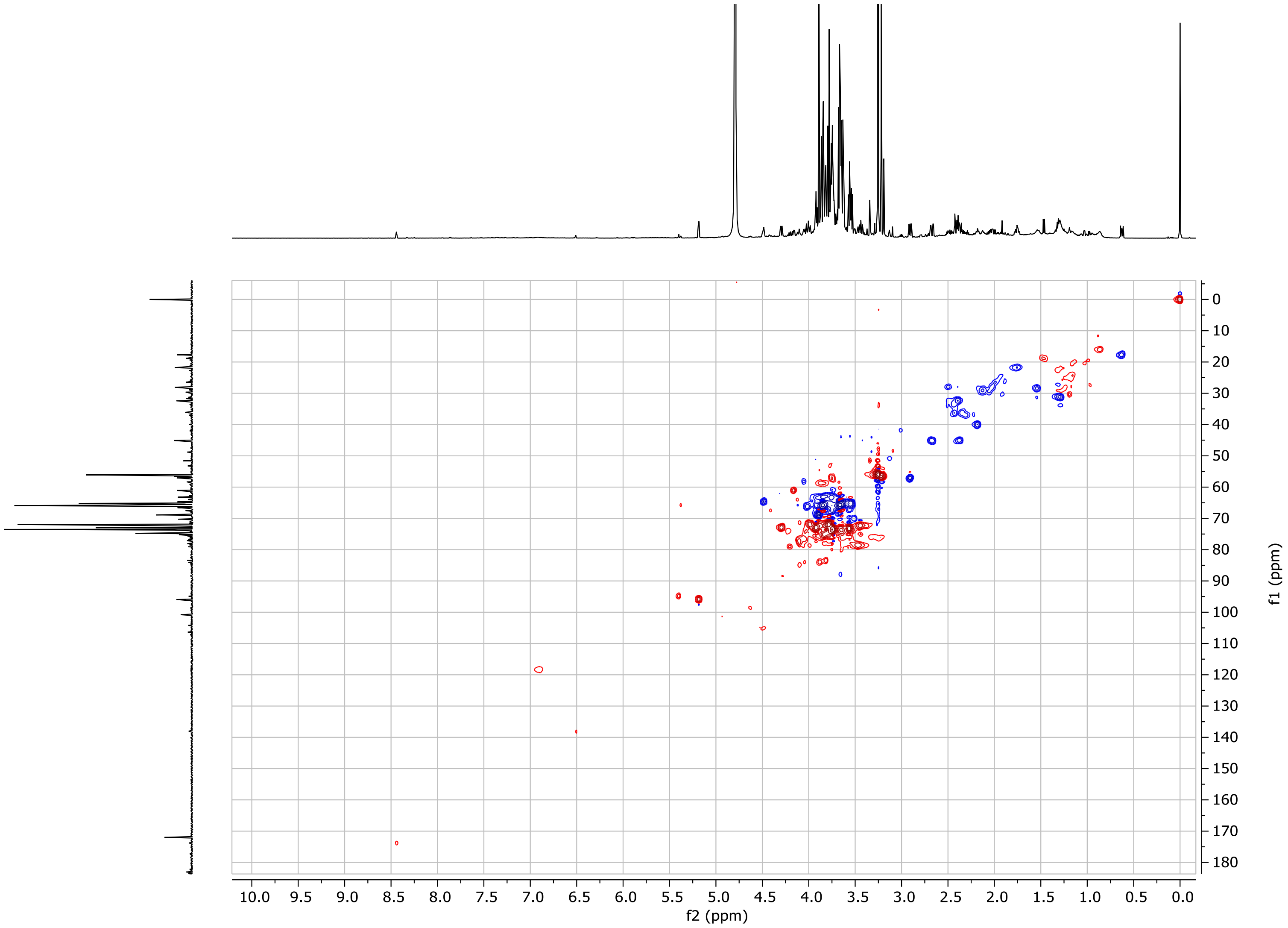


**Supplementary Figure 19.** $^{1}H$-$^{13}C$ HSQC spectrum ($D_2O$) of **PWB**

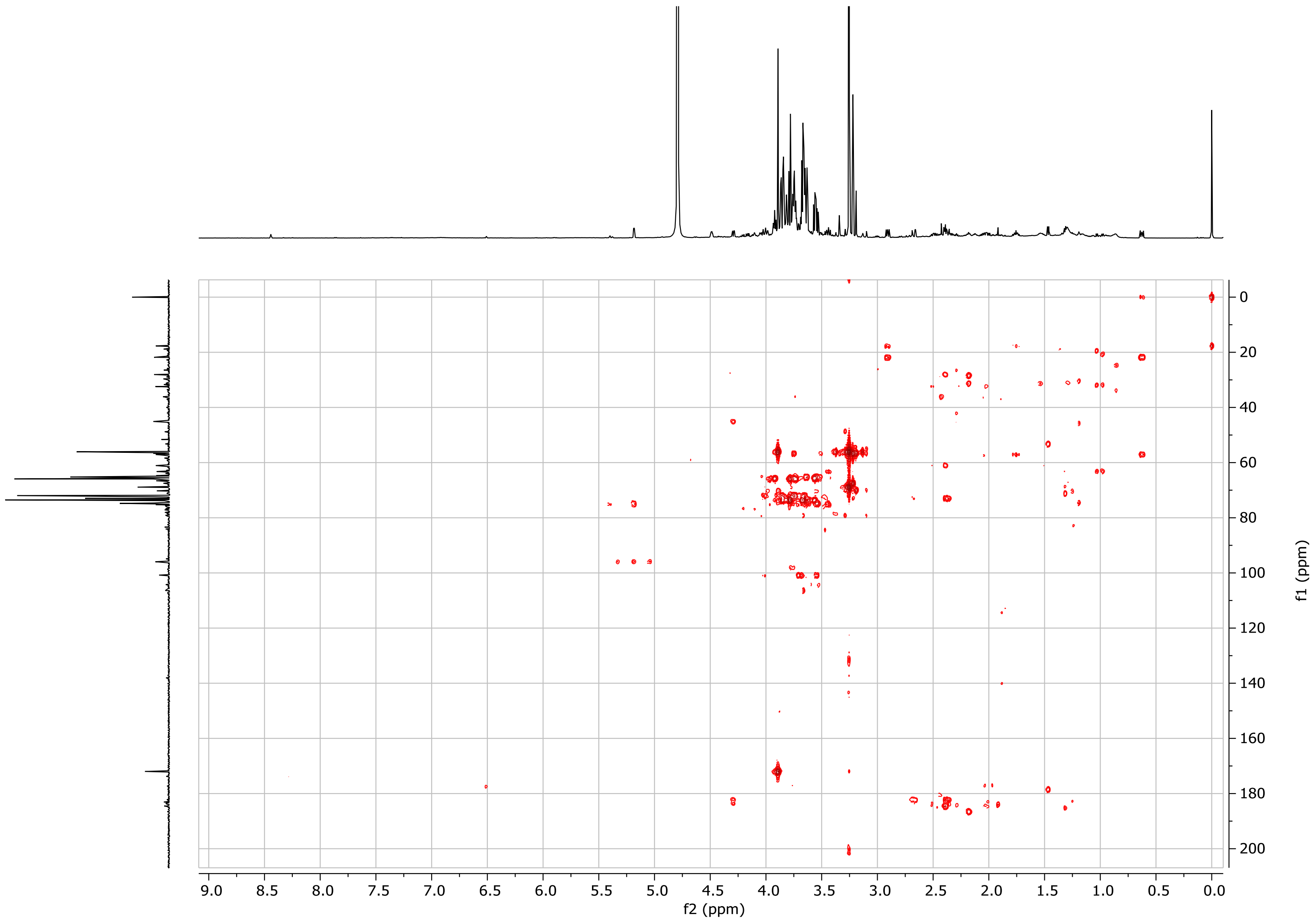


**Supplementary Figure 20.** $^{1}H$-$^{13}C$ HMBC spectrum ($D_2O$) of **PWB**

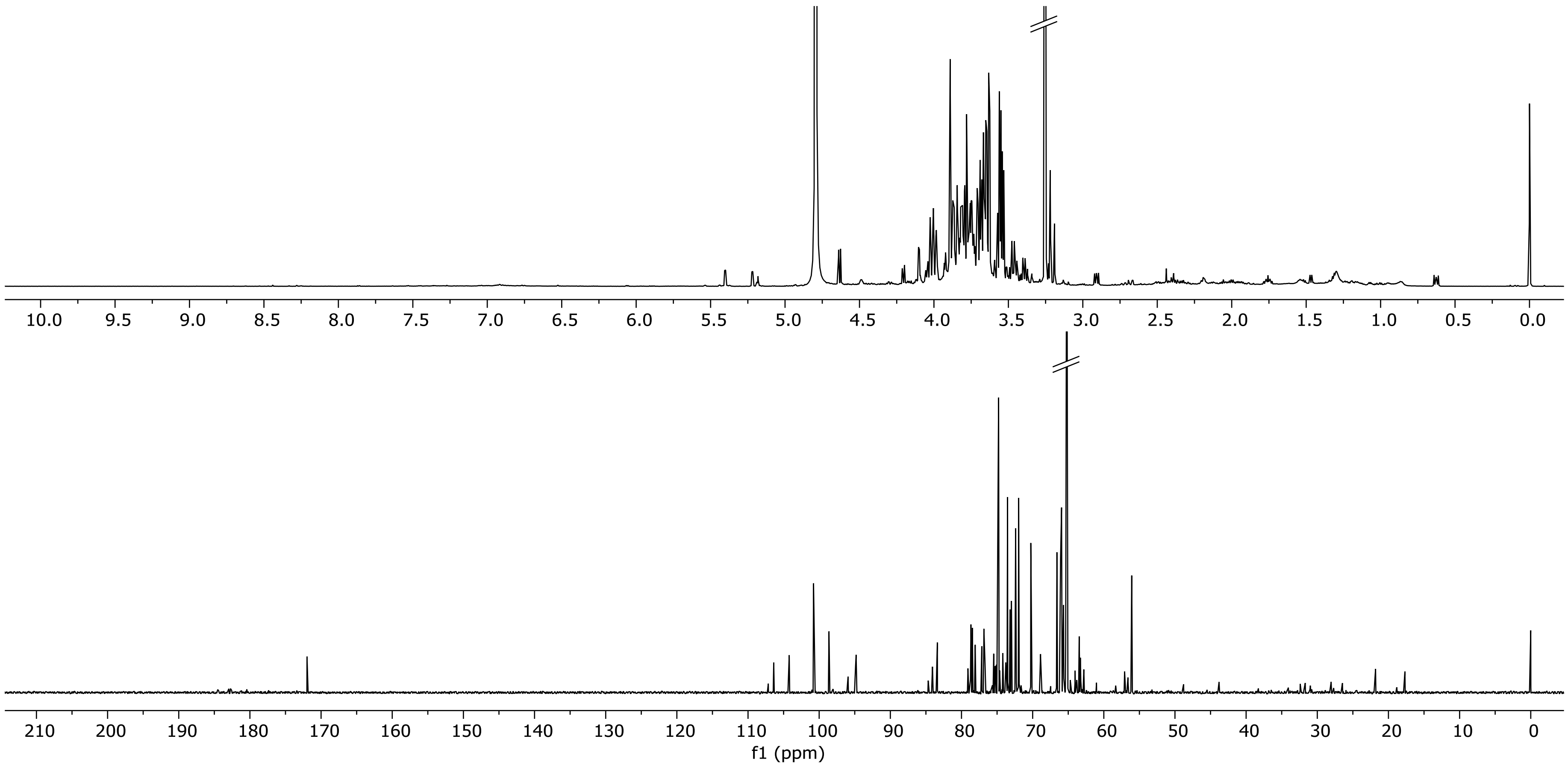


**Supplementary Figure 21.** $^{1}H$ (top, 600 MHz) and $^{13}C$ (bottom, 150 MHz) NMR spectra ($D_2O$) of **PWBr3**

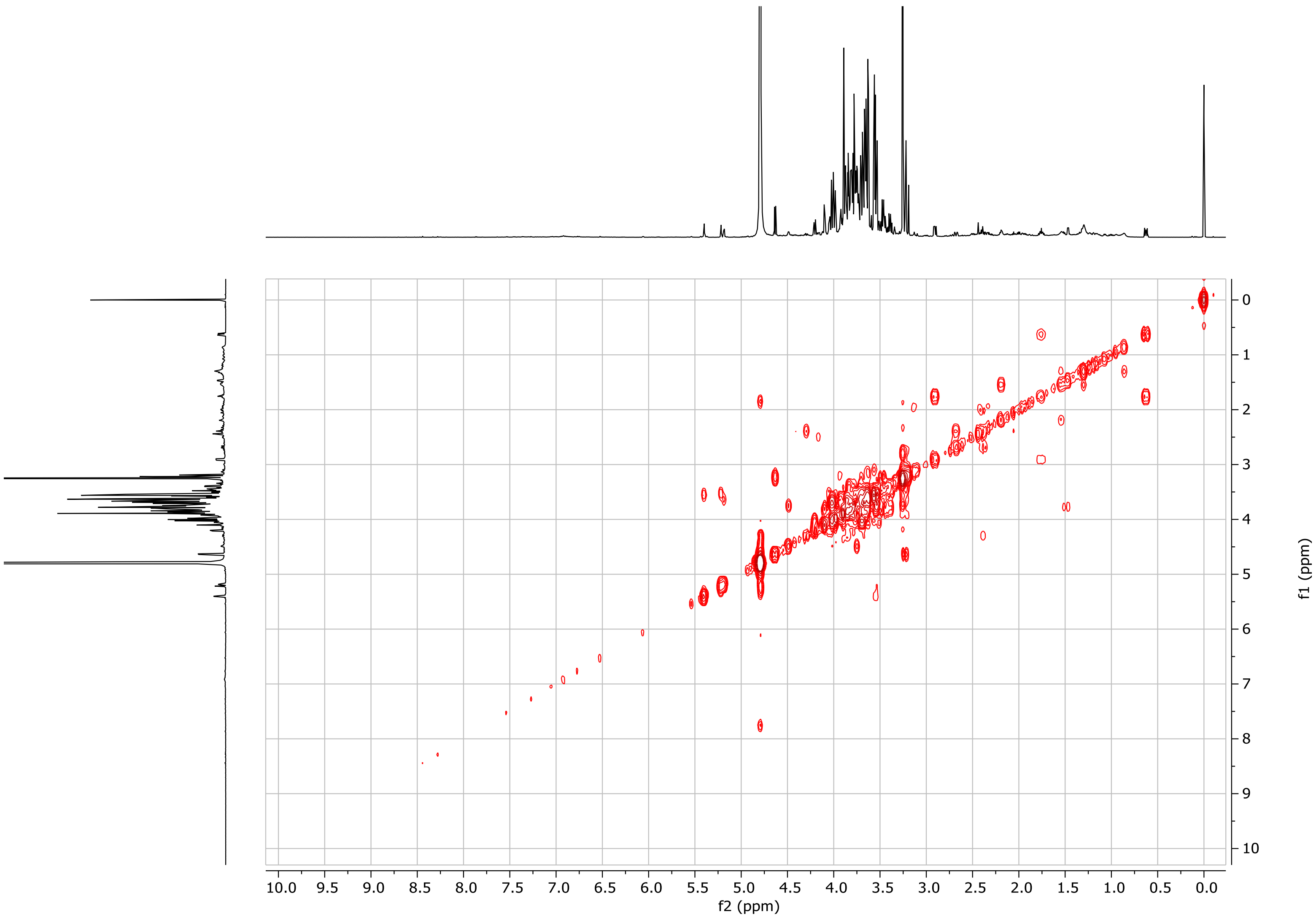


**Supplementary Figure 22.** $^1H$-$^1H$ COSY spectrum ($D_2O$) of **PWBr3**

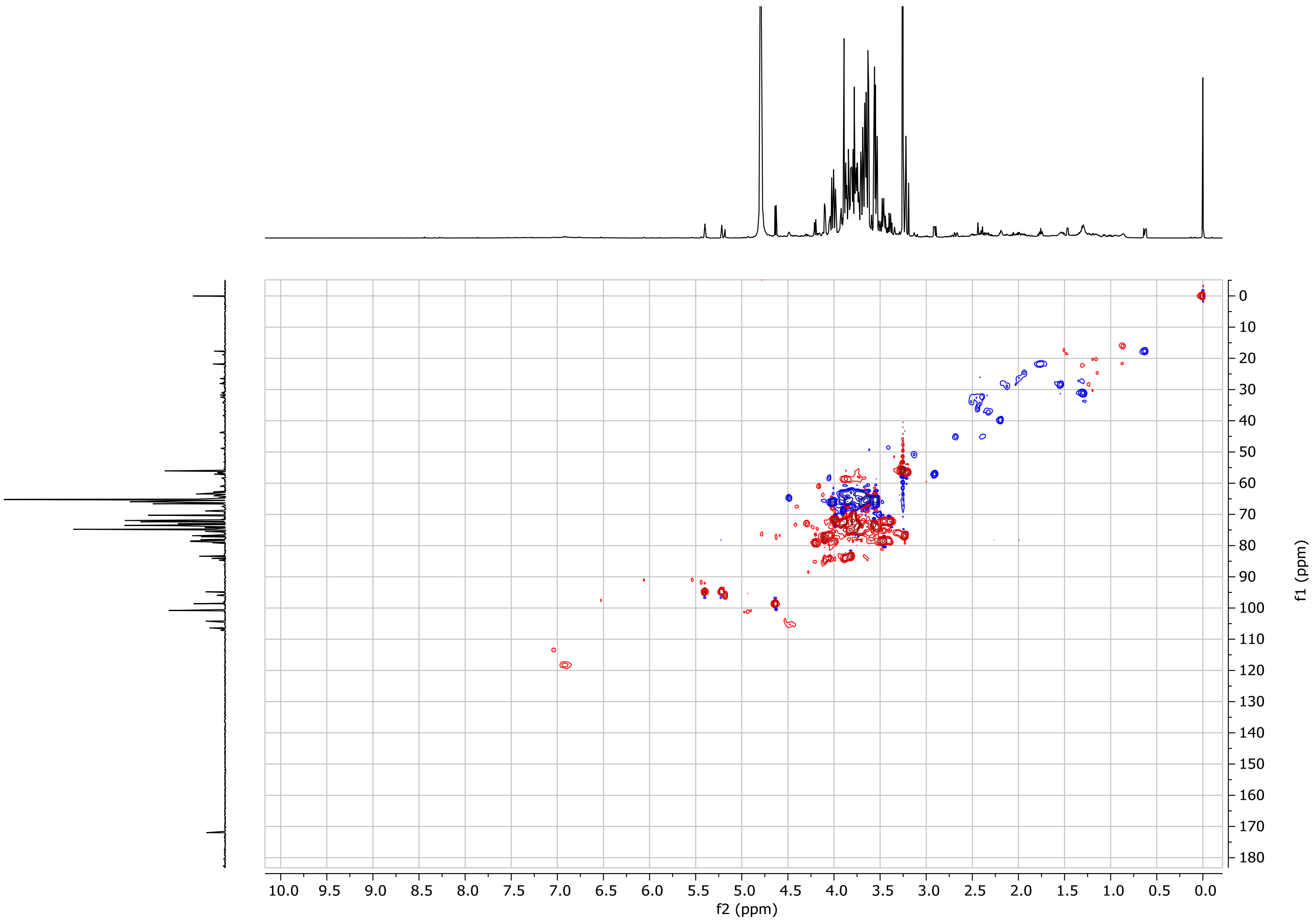


**Supplementary Figure 23.** $^{1}H$-$^{13}C$ HSQC spectrum ($D_2O$) of **PWBr3**

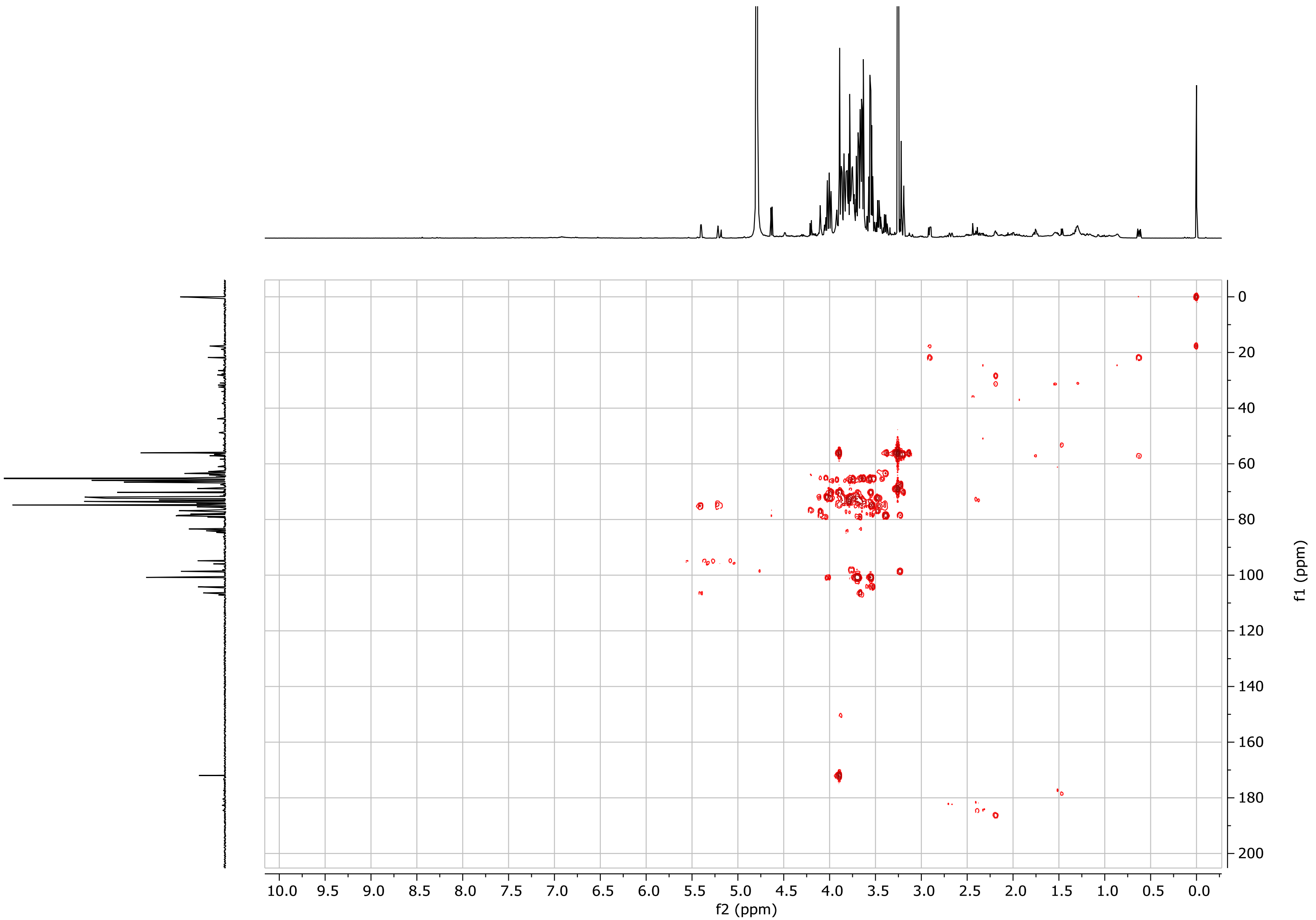


**Supplementary Figure 24.** $^{1}H$-$^{13}C$ HMBC spectrum ($D_2O$) of **PWBr3**

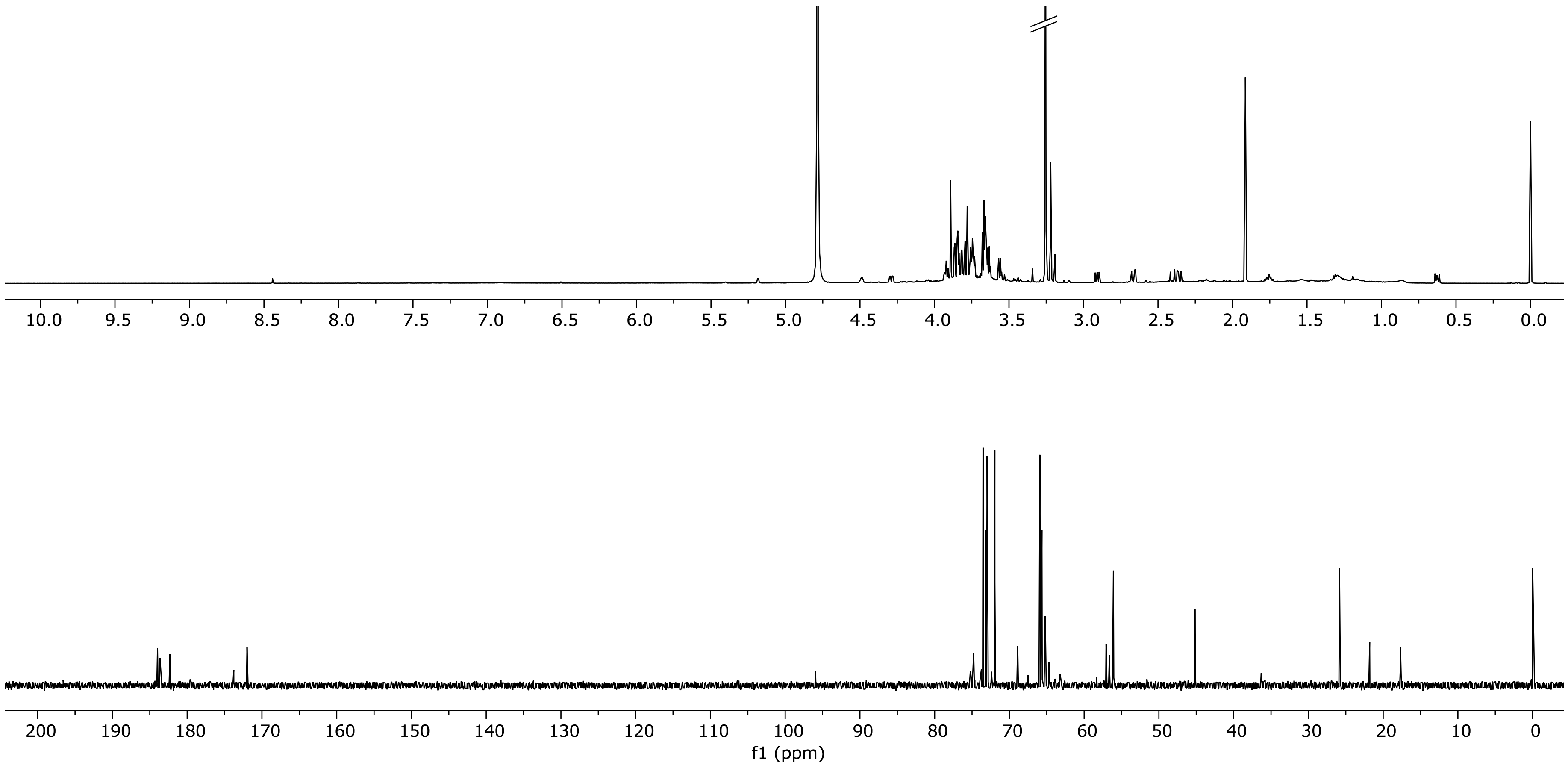


**Supplementary Figure 25.** $^{1}H$ (top, 600 MHz) and $^{13}C$ (bottom, 150 MHz) NMR spectra ($D_2O$) of **PWBr19**

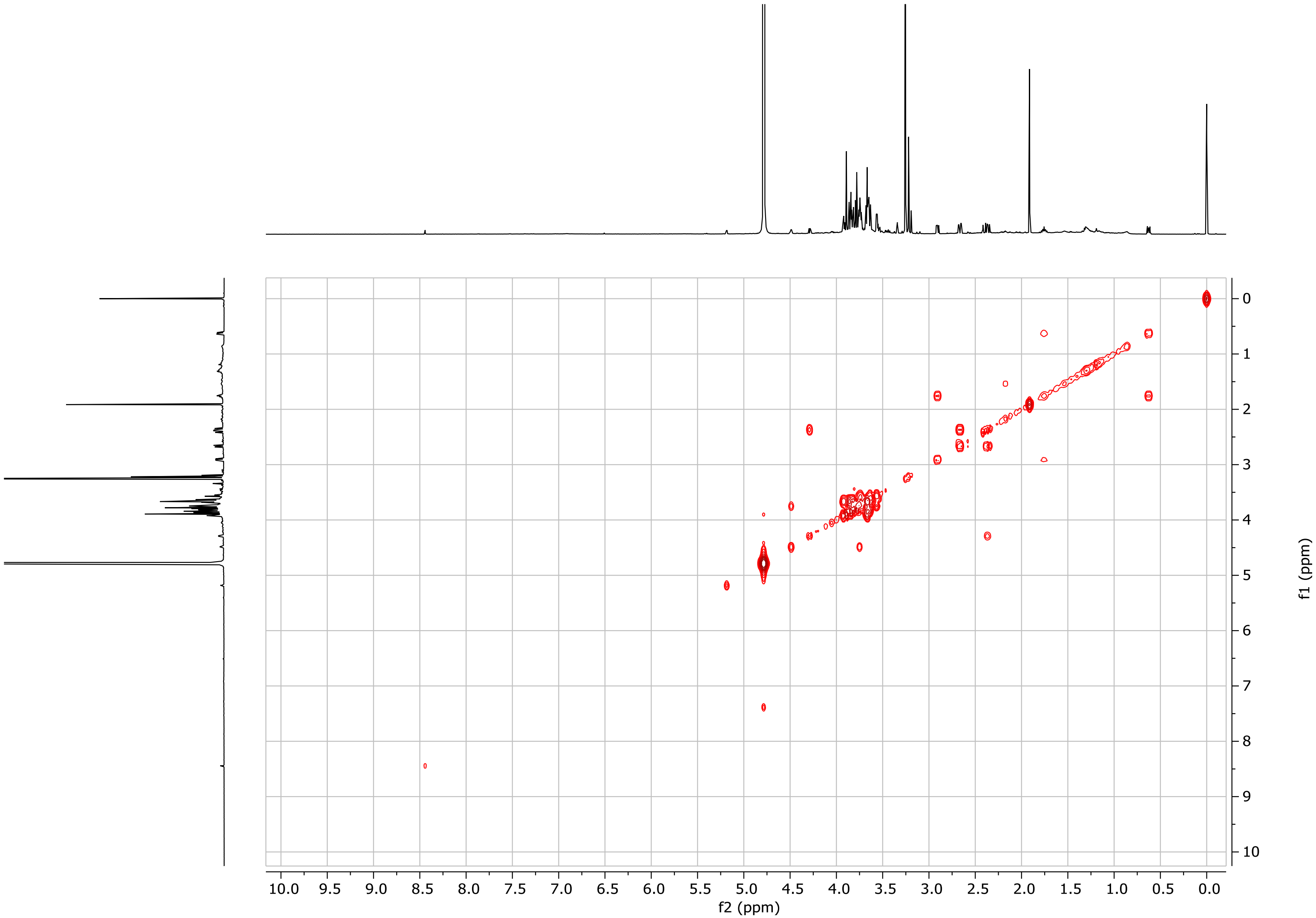


**Supplementary Figure 26.** $^{1}H$-$^{1}H$ COSY spectrum ($D_2O$) of **PWBr19**

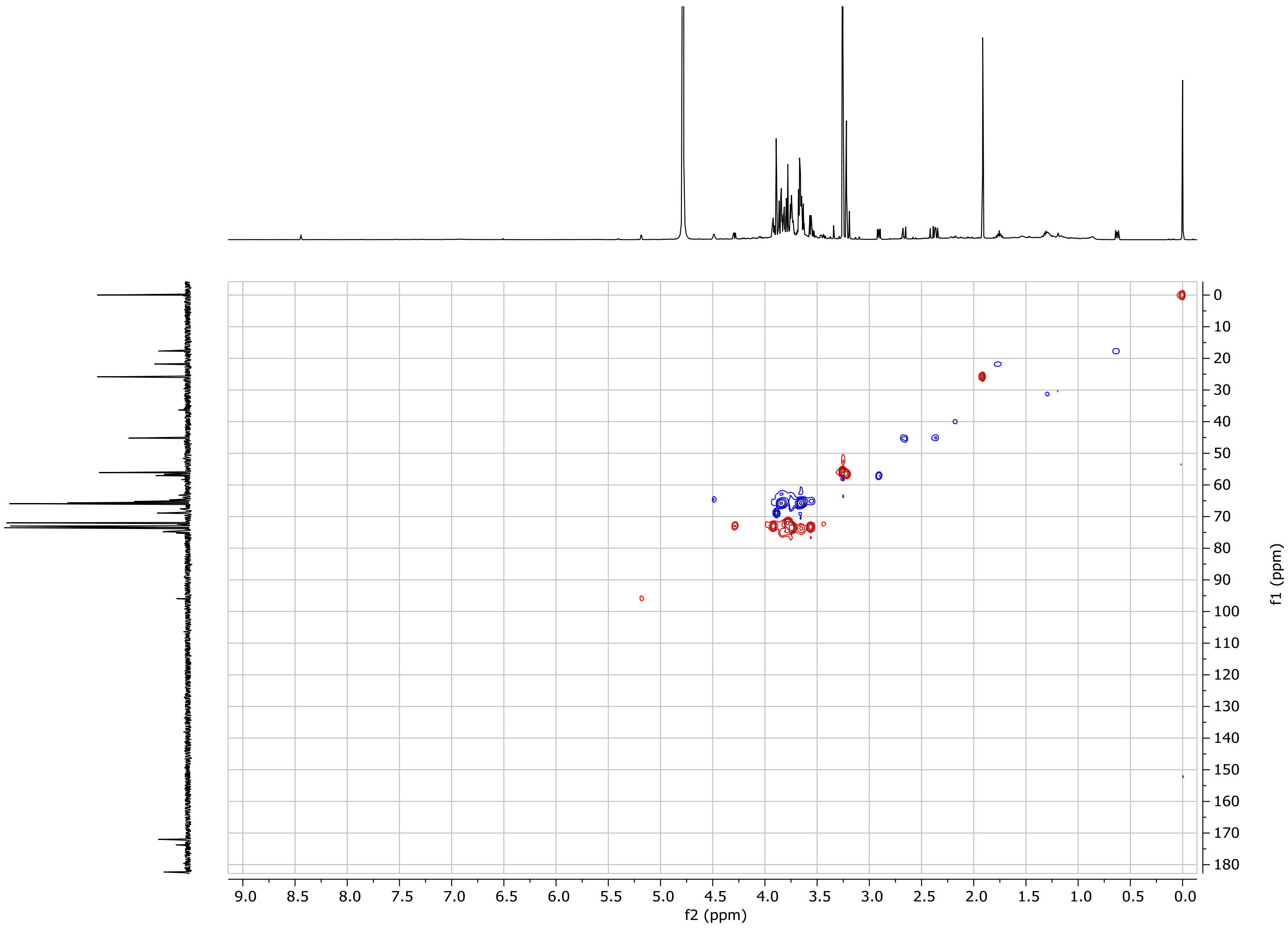


**Supplementary Figure 27.** $^{1}H$-$^{13}C$ HSQC spectrum ($D_2O$) of **PWBr19**

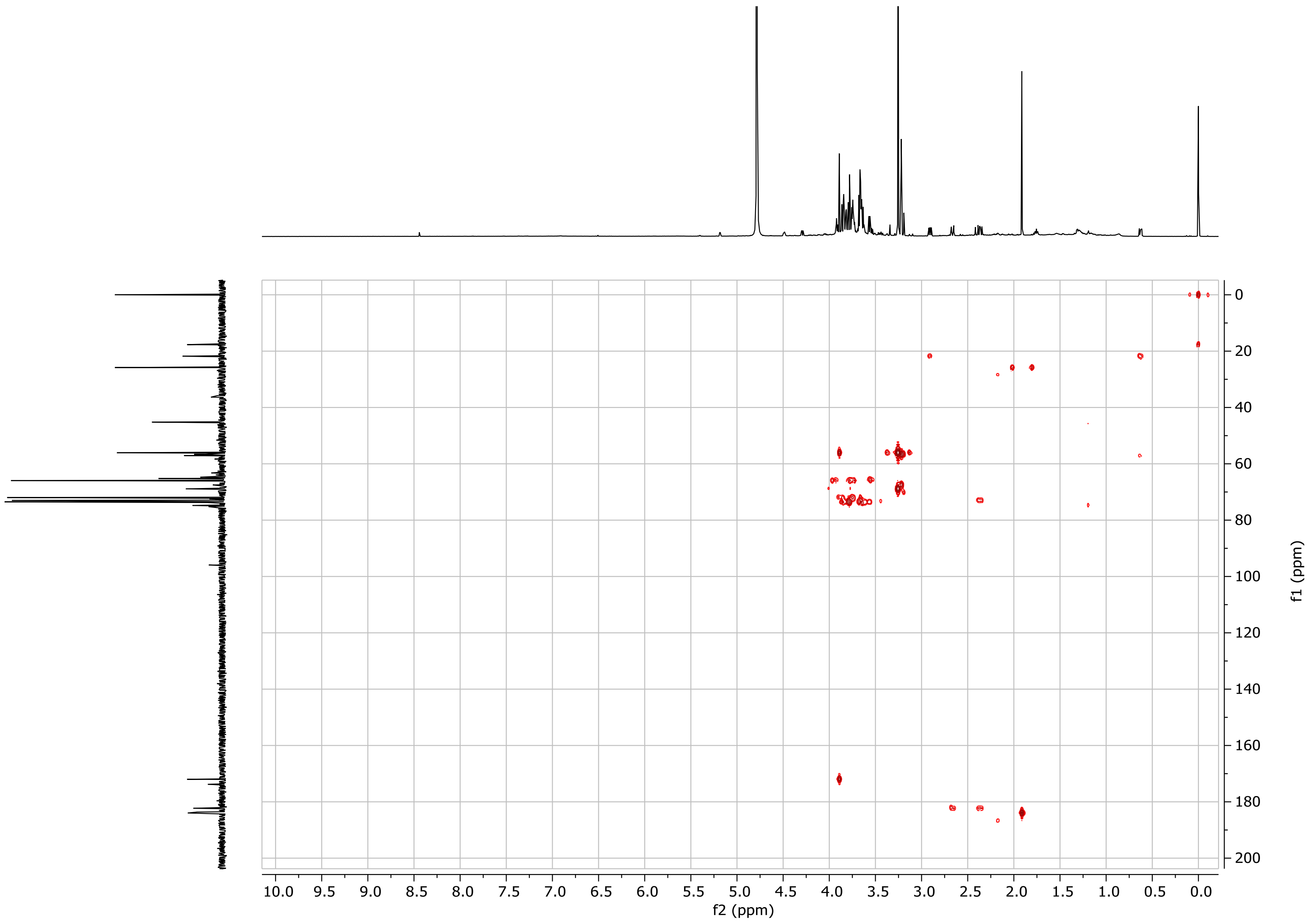


**Supplementary Figure 28.** $^{1}H$-$^{13}C$ HMBC spectrum ($D_2O$) of **PWBr19**

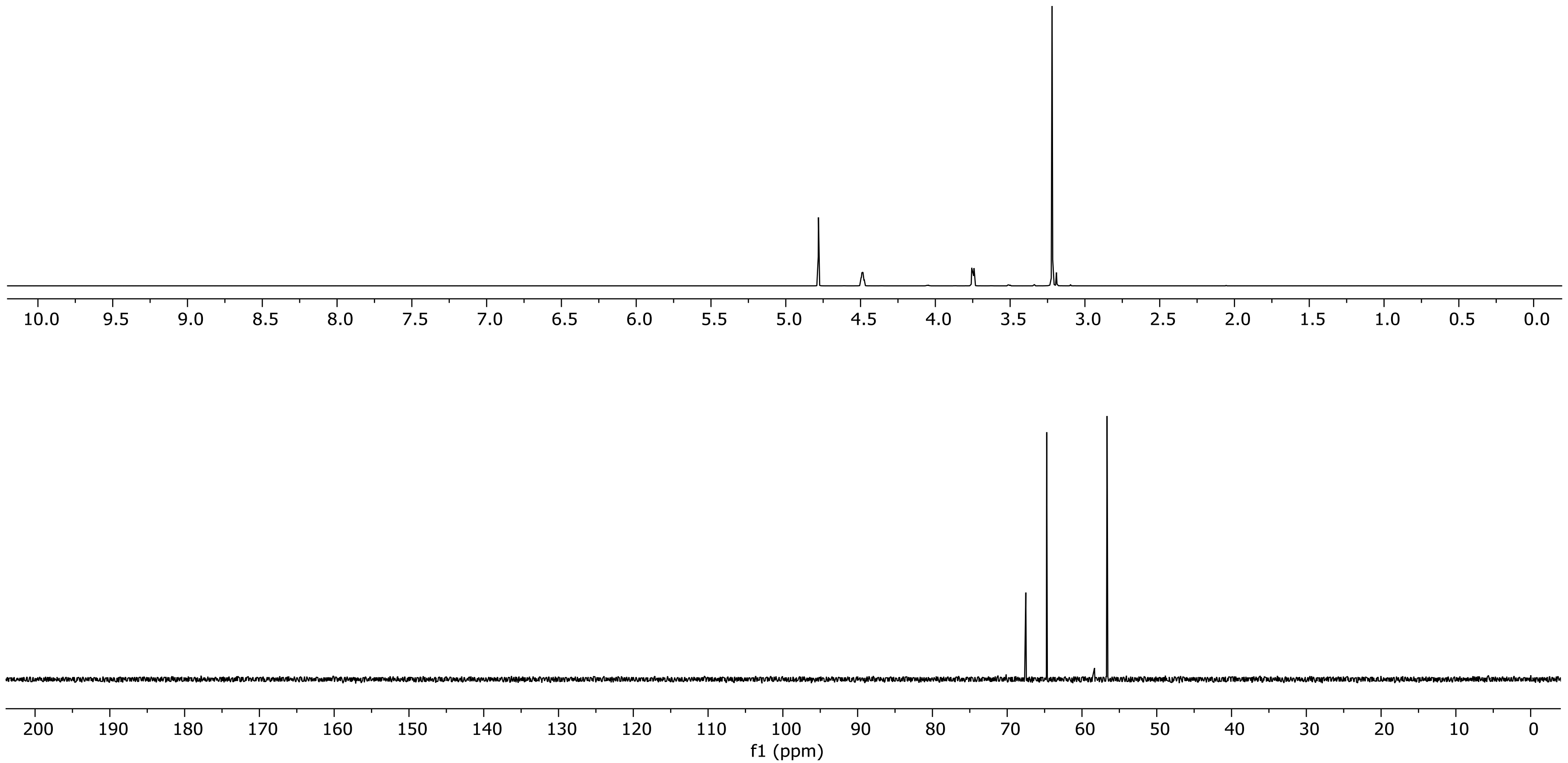


**Supplementary Figure 29.** $^{1}H$ (top, 600 MHz) and $^{13}C$ (bottom, 150 MHz) NMR spectra ($D_2O$) of choline sulfate (CS)

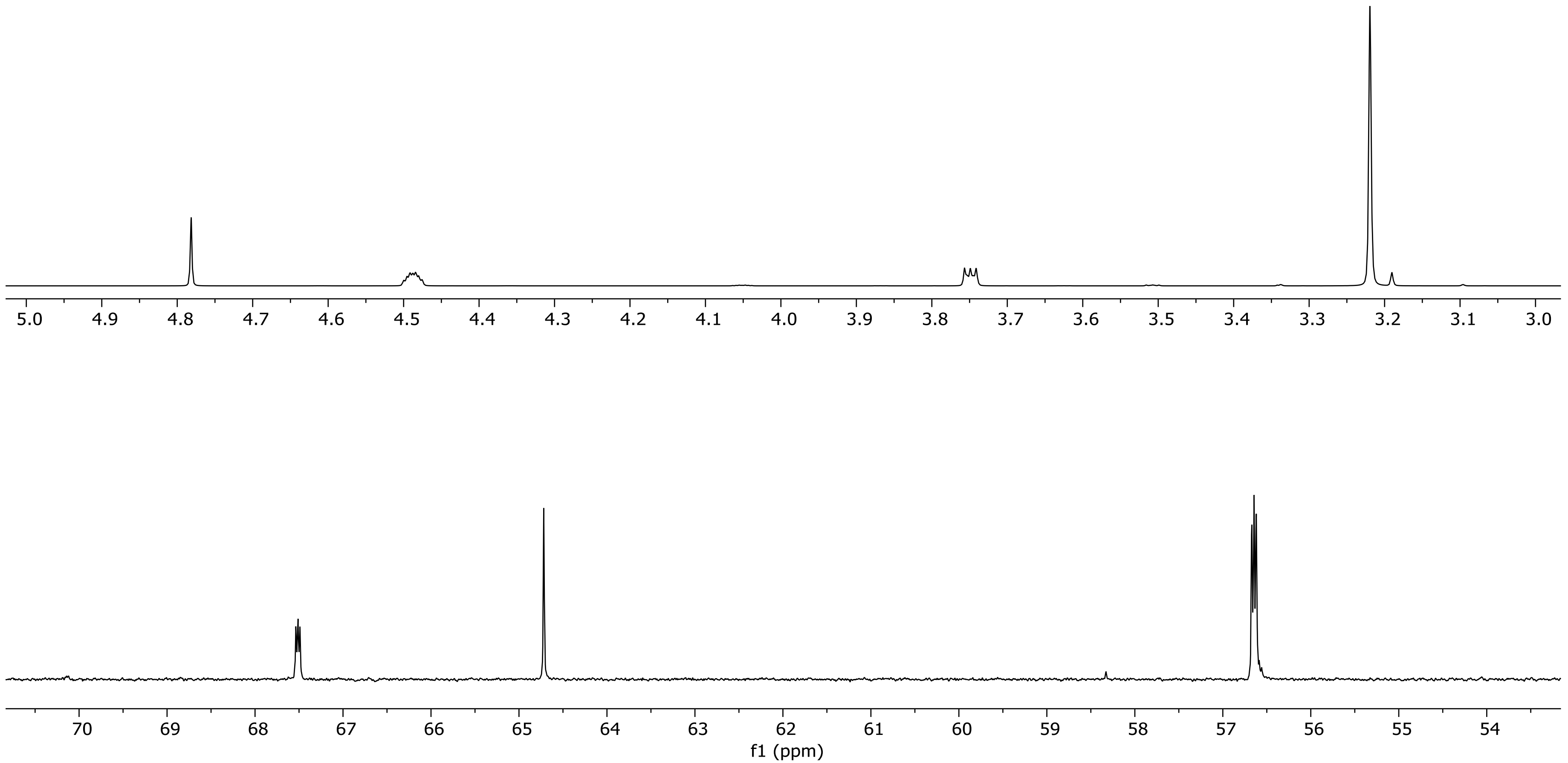


**Supplementary Figure 30.** Expanded $^{1}H$ (top, 600 MHz) and $^{13}C$ (bottom, 150 MHz) NMR spectra ($D_2O$) of choline sulfate (CS)

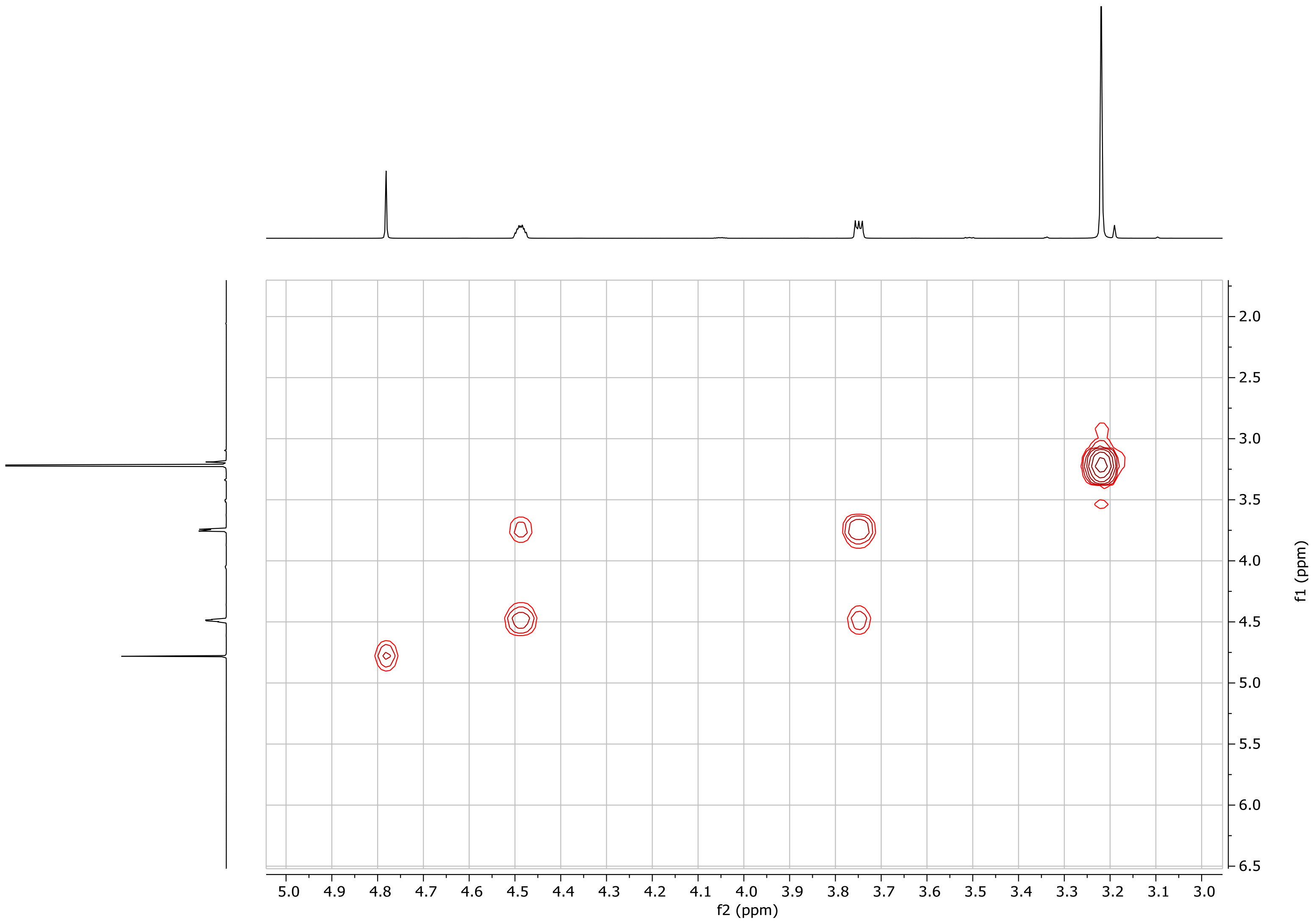


**Supplementary Figure 31.** $^{1}H$-$^{1}H$ COSY spectrum ($D_2O$) of choline sulfate (CS)

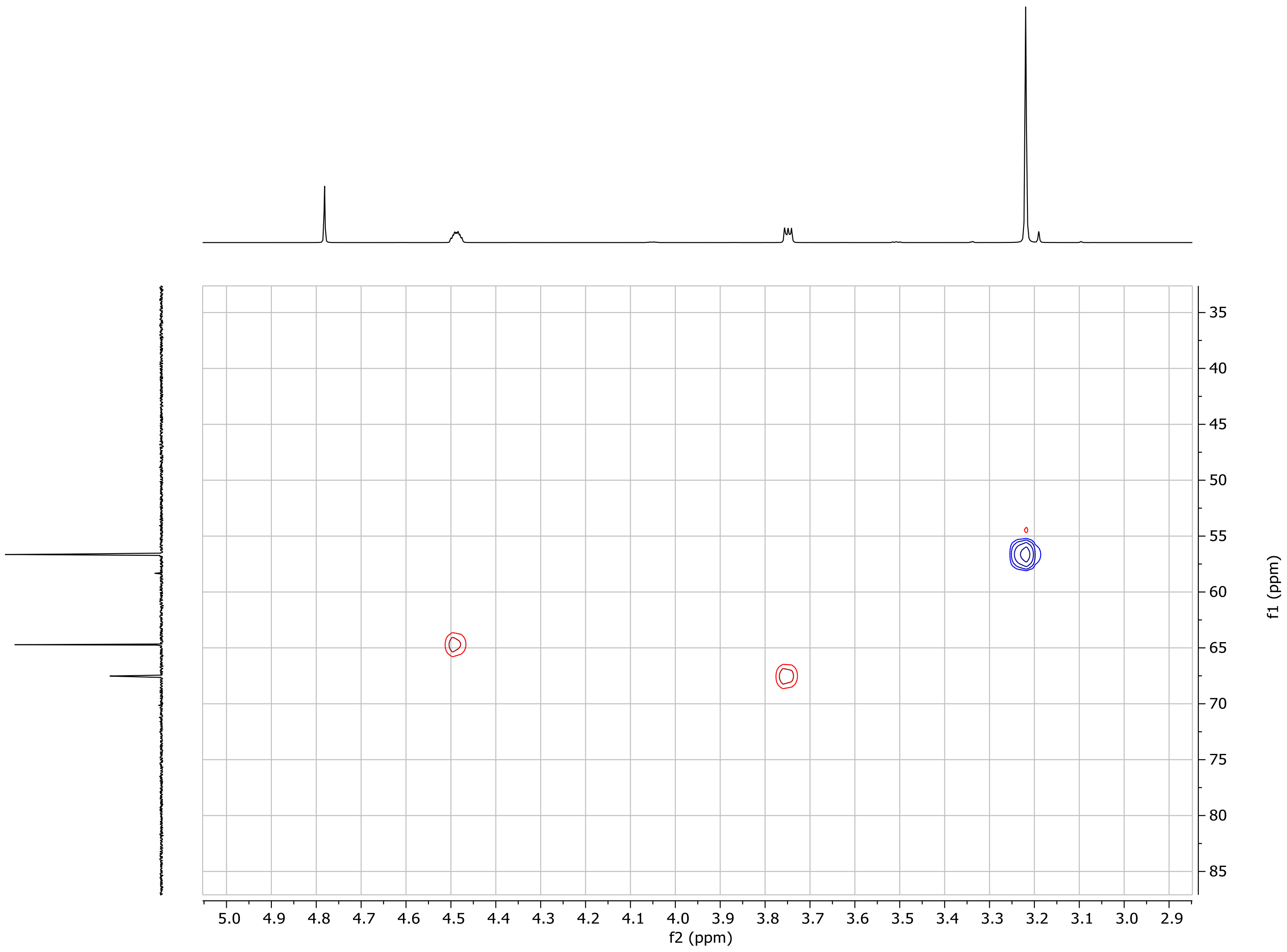


**Supplementary Figure 32.** $^1H$-$^{13}C$ HSQC spectrum ($D_2O$) of choline sulfate (CS)

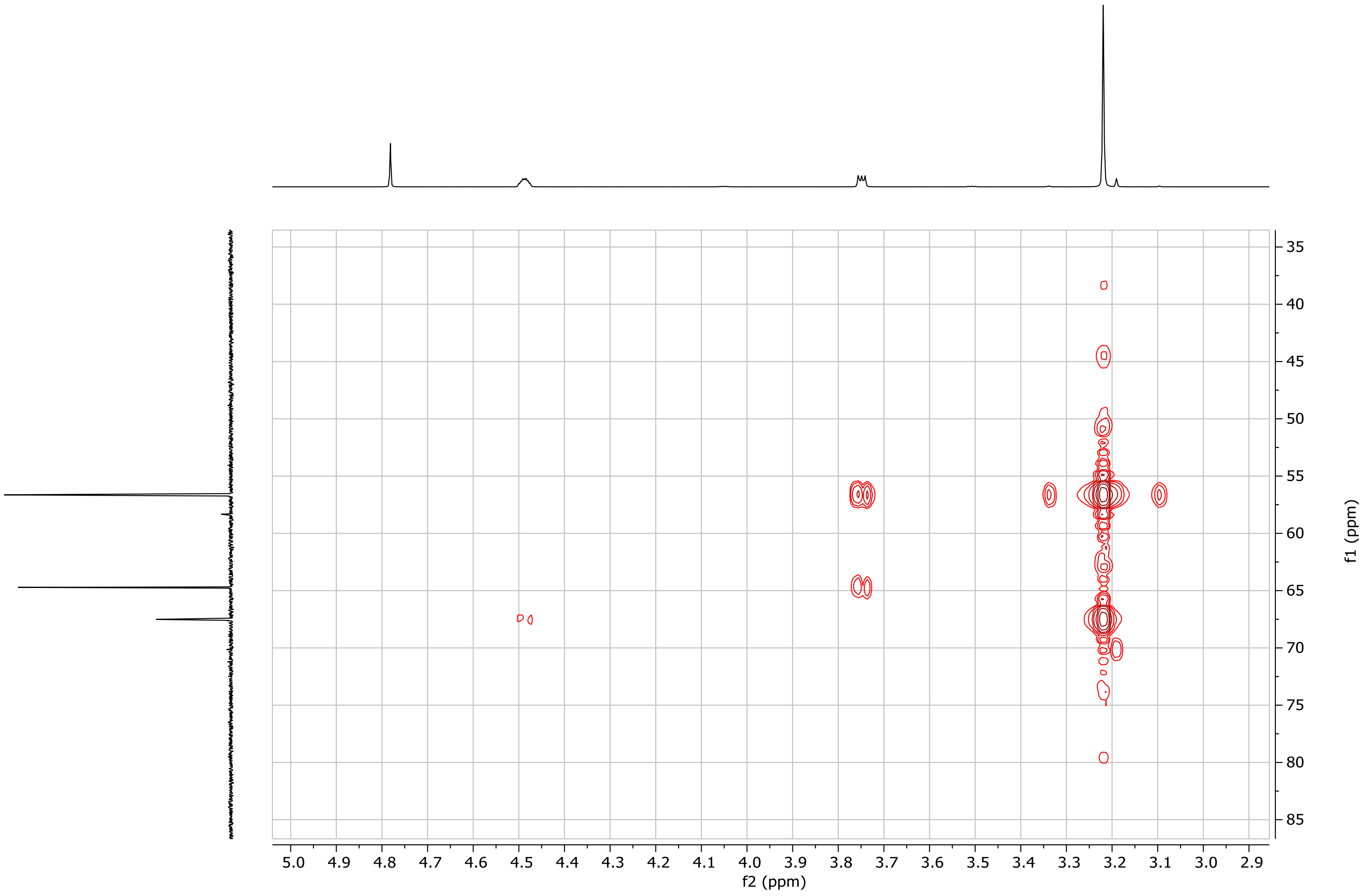


**Supplementary Figure 33.** $^{1}H$-$^{13}C$ HMBC spectrum ($D_2O$) of choline sulfate (CS)
